# Whole‐Genome Sequencing Pilot of the Central Asian Genomic Diversity Project Reveals Distinct Histories, Adaptation, and Introgression

**DOI:** 10.1002/advs.76320

**Published:** 2026-07-07

**Authors:** Mengge Wang, Shuhan Duan, Qiuxia Sun, Renkuan Tang, Lintao Luo, Jie Zhong, Zhaxylyk Sabitov, CAGDP Consortium, Lan‐Hai Wei, Chao Liu, Maxat Zhabagin, Guanglin He

**Affiliations:** ^1^ Department of Forensic Medicine College of Basic Medicine Chongqing Medical University Chongqing China; ^2^ Institute of Rare Diseases West China Hospital of Sichuan University Sichuan University Chengdu China; ^3^ Center For Archaeological Science Sichuan University Chengdu China; ^4^ Western China Forensic Medicine Center College of Basic Medicine Chongqing Medical University Chongqing China; ^5^ Chongqing Key Laboratory of Forensic Science & Chongqing Engineering Research Center of Criminal Investigation Technology College of Basic Medicine Chongqing Medical University Chongqing China; ^6^ Research Institute for Jochi Ulus Studies Astana Kazakhstan; ^7^ Kazakh Historical Society Astana Kazakhstan; ^8^ School of Ethnology and Anthropology Inner Mongolia Normal University Hohhot China; ^9^ State Key Laboratory of Bioactive Molecules and Druggability Assessment Guangdong Basic Research Center of Excellence for Natural Bioactive Molecules and Discovery of Innovative Drugs Jinan University Guangzhou China; ^10^ National Center for Biotechnology Astana Republic of Kazakhstan; ^11^ Nazarbayev University Astana Republic of Kazakhstan

**Keywords:** archaic introgression, Central Asian Genomic Diversity Project, east–west admixture, long‐range migration, post‐admixture adaptation

## Abstract

Central Asians are underrepresented in genomic research, limiting insights into their genetic history and disease risk. We established the Central Asian Genomic Diversity Project and sequenced whole genomes from 166 individuals across 20 Central Asian and Afghan Hazara groups. We identify marked differentiation driven by varying West/East Eurasian ancestry; Tajiks align with West Eurasians, Dungans with East Asians, and we report four geographically structured Turkic‐related clusters, two Indo‐European clines, and long‐range migration events, including Siberian links in Hazaras and Sino‐Tibetan ties in Dungans. Admixture dates cluster ∼650–1000 years ago, coinciding with the Song–Yuan era and Mongol expansion. We characterize distinct distributions of medically relevant variants and population‐specific adaptation signatures across metabolic, immune, and neurological pathways, and illuminate shifts in subsistence practices correlated with trait‐associated variation. We also detect Neanderthal‐like and Denisovan‐like segments that show group‐specific associations with immunity, psychiatric risk, drug metabolism, and diabetes, underscoring the scientific imperative for a broader characterization of Central Asian evolutionary history and informing precision medicine.

## Graphical abstract

As a pilot phase of the Central Asian Genomic Diversity Project, whole‐genome sequencing of 166 individuals from 20 Central Asian and Afghan Hazara populations reveals fine‐scale substructure shaped by repeated trans‐Eurasian migration and admixture. Integrated analyses uncover post‐admixture adaptation, archaic introgression, and medically relevant variation, offering new insight into how demographic history continues to influence health‐related traits across Central Asia.

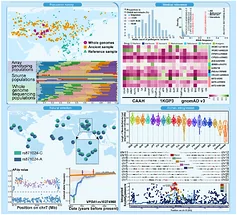

## 1 Introduction

Central Asia lies at the geographic center of Eurasia and has long served as a crossroads for the movement of people, languages, goods, and ideas between East and West Eurasia [1, 2, 3]. Ancient DNA work has revealed that Paleolithic and early Holocene Central Asia hosted distinctive populations whose ancestries traced to sources related to Ancient North Eurasians (ANE) and West Siberian hunter‐gatherers [2, 3, 4]. Later waves of gene flow from Western Steppe herders and Iranian or Anatolian farmers reshaped these ancestries and coincided with the spread of Indo‐European languages [3, 4, 5]. From the Iron Age onward, additional movements from southern Siberia and East Asia, including those associated with proto‐Mongolic and proto‐Turkic expansions, as well as the rise of the Xiongnu and Hunnic confederations, further transformed Central Asian gene pools [2, 6, 7]. Subsequent trans‐Eurasian cultural communications and political formations added further layers of complexity [2, 4, 5, 6, 8]. From the fifth century Common Era onward, the Turkic, Uyghur, Khitan, and Mongol empires succeeded the Xiongnu/Hunnic confederations and propelled large‐scale mobility across Eurasia; genetic, archaeological, and historical evidence indicates a profound impact of Mongol westward expansion on present‐day Central Asian populations [6, 9, 10].

Traditional SNP‐array‐based studies of population genetics and large medical cohorts have identified broad patterns of human genetic variation and uncovered shared genetic architectures underlying many traits and diseases [11, 12]. Meanwhile, anthropologically focused resources like the Simons Genome Diversity Project (SGDP) [13], which typically sequenced only two or three individuals per population, discovered rare and population‐specific variants and enabled reconstructions of deep demographic history, as well as valuable annotations of clinical variation. More recently, large‐scale whole‐genome efforts have reshaped human genomics by enabling finer‐scale resolution of demographic dynamic change, adaptive history, archaic introgression, and the genetic basis of complex traits and disease [13, 14, 15, 16, 17]. A clear illustration of the value of deep whole‐genome sequencing (WGS) comes from comparisons between SNP‐based surveys and sequencing‐based analyses in the Human Genome Diversity Project (HGDP), which highlighted how comprehensive sequencing in ethnolinguistically diverse populations accelerates variant discovery and strengthens inference of evolutionary mechanisms and biological function [11, 18]. Advances in third‐generation sequencing and computational methods, along with telomere‐to‐telomere and pangenome references, have enabled comprehensive analyses of complex genomic structural variation across ethnolinguistically diverse populations [19, 20, 21, 22]. However, most genomic research, such as the UK Biobank and the TOPMed Program, still focuses on individuals of European descent, which limits representation, hampers mechanistic understanding, and exacerbates health disparities [23, 24, 25, 26, 27, 28, 29]. In response, large‐scale initiatives across‐continent populations have prioritized non‐European cohorts, including the H3Africa [30], SGDP [13], GenomeAsia 100K [31], and Mexican Biobank [32], in addition to regional resources from Oceania, Pakistan, Japan, and China, such as BioBank Japan [33], China Kadoorie Biobank [34], Yanhuang cohort [35, 36, 37, 38], Westlake BioBank for Chinese [39], NyuWa [40], ChinaMAP [41], and the 10K Chinese People Genomic Diversity Project (10K_CPGDP) [8, 20, 42]. Collectively, these efforts expand the global catalog of human variation and refine models of demographic history and the evolutionary origins of disease. Present‐day Central Asian populations remain underrepresented in human genomic sequencing efforts and medical genome research despite their rich history [24].

Contemporary Central Asia is home to ethnolinguistically diverse Altaic‐speaking populations, including Turkic and Mongolic speakers (e.g., Kyrgyz, Kazakh, Uyghur, Uzbek, Karakalpak, Turkmen, Mongolian, and Kalmyk), Indo‐European speakers (e.g., Tajik, Russian, Hazara, Armenian, and Ukrainian), and groups with distinct linguistic affiliations such as Dungan, Korean, and Chechen. Turkic groups constitute most of the population in almost all Central Asian countries, except Tajikistan. Ancient DNA studies indicated that early medieval and historic Central Steppe Türk groups carried a mixture of West Eurasian and Ancient Northeast Asian (ANA) ancestries [43]. Genome‐wide SNP analyses showed that most modern Turkic groups retain shared ancestry with populations in southern Siberia and Mongolia [10], and a recent study of Tajik and Kyrgyz populations found that Historical‐Era gene flow from the Eastern Steppe made substantial contributions to Central Asian Turkic speakers [1]. Early work based on low‐density markers further suggested exceptionally high diversity in Central Asia [44]. More recent genome‐wide single‐nucleotide polymorphism (SNP) studies reported extensive admixture among European, West Asian, East Asian, and South Asian sources [4, 10, 45, 46]. Ancient DNA has further clarified the layered contributions of ANE, West Siberian hunter‐gatherers, Western Steppe herders, and Iranian‐ and Anatolian farmer‐related ancestries to Central Asia over time. These findings suggest that Central Asia has acted as both a recipient and a source of population movements across Eurasia, preserving genomic signals that connect Northern China, the Mongolian Plateau, Siberia, Chinese Xinjiang, and Central Asia [24]. As shown by populations such as various Chinese Hui and Turkic‐speaking groups [47, 48], understanding the population history of East Asia and Eurasia more generally requires explicit consideration of Central Asian genomic variation, especially at the whole‐genome level. Nevertheless, key questions remain unresolved. First, we still lack a fine‐scale, whole‐genome perspective on how these evolutionary processes shaped the genetic structure and demographic history of contemporary Central Asian populations. Second, the genetic consequences of evolutionary forces and events for local adaptation and the distribution of medically relevant variants remain poorly understood. Third, the extent and functional impact of Neanderthal and Denisovan introgression in Central Asia have not been systematically characterized.

To address these gaps, we established the Central Asian Genomic Diversity Project (CAGDP) and generated high‐quality WGS data from 166 individuals across 20 Central Asian and Afghan Hazara (CAAH) populations in the pilot work, sampled in five Central Asian countries and Afghanistan. We then integrated these data with dense modern and ancient genomic resources from across Eurasia (Figure 1A,B and Table S1) to investigate how trans‐Eurasian population movements and selection shaped the genetic structure, medically relevant variation, and archaic introgression landscape of present‐day Central Asian populations. By jointly analyzing population structure, admixture, and divergence times, medically relevant and pharmacogenomic variants, and signatures of natural selection and archaic introgression, we aim to provide a comprehensive genomic portrait of Central Asia that refines models of Eurasian evolutionary history and lays a foundation for future precision medicine efforts in this historically understudied region.

**FIGURE 1:**
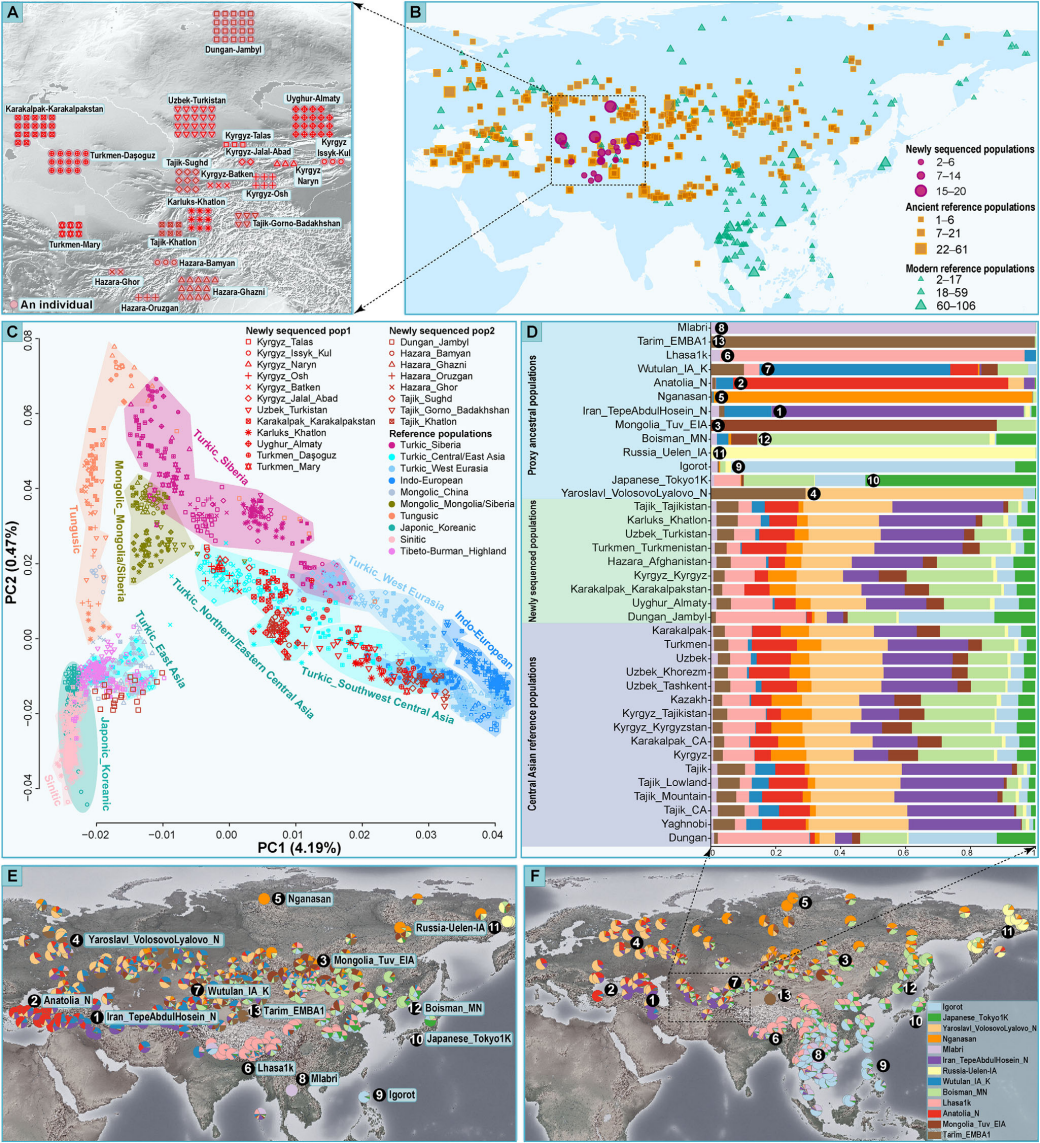
Geographic distribution and population structure of newly sequenced Central Asian and Afghan Hazara (CAAH) populations and reference populations based on the merged Human Origins (HO) dataset. (A), Sampling locations of 20 newly collected populations. Each circle represents a single individual. (B), Geographic distribution of newly sequenced populations alongside modern and ancient Eurasian reference populations from the extended HO dataset. The purple circles indicate newly sequenced populations, the orange squares represent ancient references, and the turquoise triangles denote modern references. (C), Patterns of genetic affinity between CAAH and 132 linguistically related Eurasian populations from the Altaic, Indo‐European, and Sino‐Tibetan language families. Genetic clusters or clines are indicated, with background and label colors corresponding to the groupings shown in Figure S3. Reference populations are presented by language family, with the same square colors preceding each group as in Figure S3. The specific populations comprising each group are detailed in Figure S3. (D), Model‐based ADMIXTURE analysis at K = 13, displaying the admixture profiles of Central Asian populations and proxy ancestral groups. Groups 1–13 correspond to proxy ancestral populations, with group numbers assigned arbitrarily. The colors denote distinct ancestral components. (E), Detailed admixture profiles of ancient Eurasian populations at K = 13. (F), Admixture profiles of modern Eurasian populations at K = 13.

## 2 Results

### 2.1 Variant Discovery, Genetic Structure, and Population History of CAAH Populations

We generated whole‐genome sequences for 166 individuals from 20 CAAH populations at a mean depth of 13.66×, ranging from 6.66× to 16.67× (Figure S1A). Call rates ranged from 97.9% to 99.9%, and 95.8% of CAAH individuals achieved a call rate of at least 99.0% (Figure S1B). After stringent quality control, the dataset comprised 22,994,949 SNPs and 2,952,662 small insertions or deletions (InDels) across the autosomes and X chromosome. Of these, 10.0% of SNPs (2,300,814) and 28.3% of InDels (834,296) were absent from dbSNP v156; among these novel variants, 89.4% were singletons and 9.1% had a minor allele frequency (MAF) below 0.01. Among the 22,538,640 biallelic SNPs, 40.2% were singletons, 15.1% had a MAF below 0.01 after excluding singletons, and 14.7% had a MAF between 0.01 and 0.05. We observed no significant correlation between sequencing depth and singleton count (*R* = −0.138, *p* = 0.0755), indicating that variation in depth did not substantially bias singleton discovery across samples (Figure S1C). Based on 21,608,632 biallelic autosomal SNPs, we estimated a transition‐to‐transversion ratio (Ti/Tv) of 2.05. Ti/Tv ratios remained within the expected range across allele frequency bins, ranging from 2.01 to 2.13 (Figure S1D), consistent with the quality of Tibetan genomes with similar depth [49], supporting high‐quality genotypes for subsequent population genetic analyses.

We conducted principal component analysis (PCA) on the WGS dataset to characterize the genetic structure of CAAH populations. CAAH populations fell between the West and East Eurasian clines. Within this continuum, Tajiks clustered toward West Eurasians, whereas Dungans clustered with East Eurasians (Figure S2). After integrating additional modern and ancient data [4, 10, 50], non‐Dungan CAAH populations aligned along a West Eurasian‐related cline. Dungans occupied the northern branch of the East Eurasian‐related cline (Figure S3). Tajiks and Karluks showed strong affinities to Indo‐European and Turkic‐speaking populations from the Caucasus, Uzbeks and Turkmens aligned with southwest Central Asian Turkic groups, and Kyrgyz, Uyghur, Karakalpak, and Hazara people clustered with Turkic groups from northern and eastern Central Asia and China's Xinjiang. Dungans showed close genetic connections to Mongolic, Tibeto‐Burman, and Turkic‐speaking Chinese populations. When projected onto ancient genomes (Figure S3), most non‐Dungan CAAH individuals overlapped or clustered with Iron Age Xinjiang and historical Kazakhstan populations, whereas Dungans aligned more closely with ancient Yellow River and Qinghai‒Xizang Plateau groups. Kyrgyz, Uyghur, Karakalpak, and Hazara people shifted toward ancient Mongolian Plateau populations with reduced West Eurasian ancestry, indicating enhanced East Eurasian‐related drift. In contrast, Tajiks and some Uzbek and Turkmen individuals lie closer to Iranian or Anatolian farmer‐related groups, Russian steppe populations, and ancient populations from Turkmenistan, Uzbekistan, Tajikistan, Afghanistan, and middle‐to‐late Bronze Age Kazakhstan. This pattern was consistent with stronger West Eurasian ancestry. Together, these patterns show that present‐day CAAH populations integrate long‐term contributions from West Asian farmers, steppe pastoralists, and multiple East Eurasian sources. Further analysis of genetic coordinates for CAAH and linguistically similar groups revealed a clear east–west genetic gradient among Turkic‐speaking groups across Eurasia (Figure 1C). Indo‐European speakers followed two main genetic gradients: one comprising Armenian, Kurd, Tajik, and Yaghnobi individuals from southern Central Asia, the Caucasus, and West Asia, and the other comprising Russian, Ukrainian, and German individuals from Eastern Europe (Figure 1C).

Model‐based ADMIXTURE analysis corroborated this structure and revealed stratified ancestry profiles across CAAH populations (Figures S4 and S5). At K = 13, where cross‐validation error plateaued (Figure S4), Kyrgyz and Karakalpak groups derived substantial ancestry (> 10% each) from ANA (Boisman_MN), Eastern European hunter‐gatherer (EEHG; Yaroslavl_VolosovoLyalovo_N), and Iranian farmer‐related (TepeAbdulHosein_N) sources, with smaller contributions (2%–10% each) from ancient northern East Asian (ANEA; Lhasa1k), Neo‐Siberian (Nganasan), Japanese, southern East Asian (SEA; Igorot), Anatolian farmer (Anatolia_N), and ANE‐related (Tarim_EMBA1) ancestries (Figure 1D–F). Uyghur, Hazara, Uzbek, and Turkmen populations shared these components but differed in their proportions. Uyghur and Hazara were enriched for Iranian farmer, ANA, EEHG, and ANEA ancestries, whereas Uzbek and Turkmen groups harbored higher levels of Iranian farmer, EEHG, ANA, and Anatolian farmer ancestries. Karluk's ancestry was dominated by Iranian farmer‐ and EEHG‐related components, with minor contributions from ANA, Anatolian farmer, ANEA, ANE, Japanese, Neo‐Siberian, SEA, and ancient Xinjiang populations (Wutulan). Tajiks' ancestry was mainly composed of Iranian farmer‐, EEHG‐, and Anatolian farmer‐related ancestries, with smaller proportions from ANE‐, ANA‐, ANEA‐, Wutulan‐, and Japanese‐related ancestries. Dungans carried predominantly East Asian (ANEA, SEA, Japanese) and ANA ancestries with minor West Eurasian input. We then used outgroup *f*_3_‐statistics and TreeMix analyses to test these affinities. These analyses supported the inferred relationships and highlighted differential allele sharing with both modern and ancient Eurasian populations (Figures S6–S8).

### 2.2 Geography‐Related Genetic Differentiation Among Eurasian Turkic Groups

We further investigated how geography shapes genetic variation across Eurasia using genome‐wide data from 54 Turkic‐speaking populations. PCA and ADMIXTURE analyses revealed four major clusters aligned with geography (Figure 1C,F and Figures S3 and S5). The East Asian cluster was dominated by Salar and Yugur populations from Northwest China, excluding Xinjiang; the Siberian cluster included Altaian, Khakass, Shor, Tubalar, Tofalar, Tuvinian, and Yakut populations from southern Siberia and the Russian Far East; the Central Asian cluster included Kazakh, Kyrgyz, Karakalpak, and Uyghur populations from northern and eastern Central Asia and Chinese Xinjiang; and the West Eurasian cluster included Turkmen, Uzbek, Nogai, Balkar, Bashkir, Kumyk, and Tatar populations from the Caucasus, Eastern Europe, West Asia, western Siberia, and southwestern Central Asia (Figure 1C). Pairwise Welch's t‐tests confirmed significant geography‐related substructure within Turkic groups (Figure 2A). TreeMix‐derived topology supported distinct population structures and revealed complex gene flow among groups (Figure S9). To explore the factors underlying these differences, we examined correlations between geographic variables (latitude and longitude) and genetic features (PC scores and ancestry proportions). The first two PCs correlated strongly with longitude and latitude, demonstrating that genetic differentiation among Turkic groups followed east–west and north–south geographic gradients (Figure 2B,C). Neo‐Siberian‐, ANA‐, and ANE‐related ancestries increased from south to north (*r* < 0.30, *p* < 0.05), whereas East Asian‐ and Iranian farmer‐related ancestries showed the opposite trend (0.31 ≤ |*r*| ≤ 0.52, *p* < 0.05). East Asian and Neo‐Siberian‐related ancestries increased from West to East (0.40< *r* < 0.60, 0.001< *p* < 0.01), whereas Anatolian farmer, Iranian farmer, and EEHG‐related components showed the opposite trend (|*r*| ≥ 0.67, *p* < 0.001). Interestingly, the relationships between geographic distance and genetic differences among the Altaic groups differed slightly from those within the Turkic groups (Figure S10). Among Altaic speakers, ANE‐related ancestry showed a weak negative correlation with longitude (*r* = −0.37, *p* < 0.001), whereas no substantial correlation with latitude was observed within Turkic groups for this component.

**FIGURE 2:**
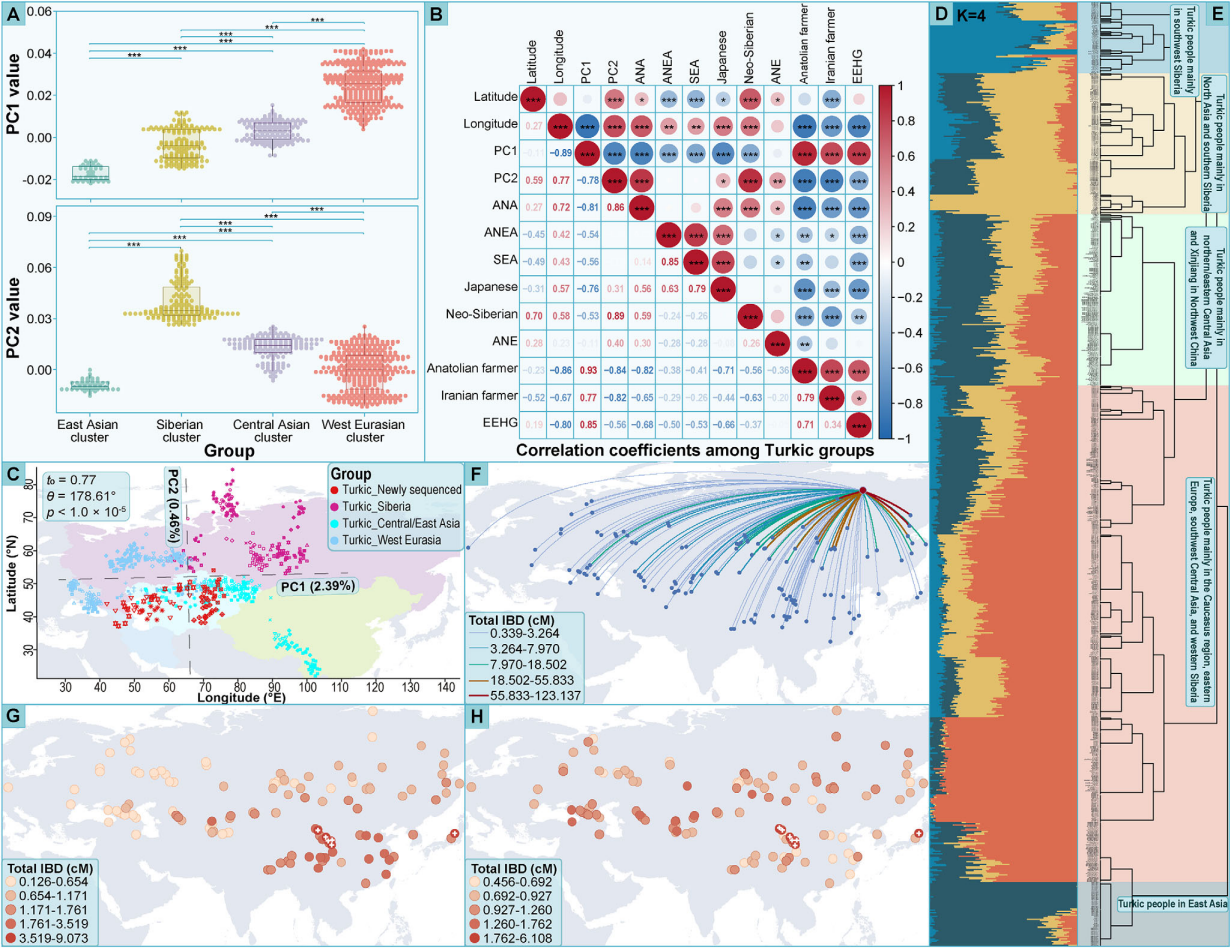
Geographic and genetic correlations, fine‐scale structure of Turkic groups, and identity‐by‐descent (IBD) sharing among Eurasian populations. (A), Pairwise Welch's t‐tests of the first two PCs among four Turkic‐related clusters, as defined in Figure 1C. Turkic‐speaking populations from southwestern Central Asia and western Siberia were assigned to the West Eurasian cluster. "***" indicates *p* < 0.001. (B), Correlations between geographic coordinates (latitude, longitude) and genetic features (PC1, PC2, and ancestral components) among 54 Turkic groups. The lower left panel displays correlation coefficients (r), with negative (blue), positive (red), and neutral (0) values. The upper right panel presents *p*‐values, with circle color (blue to red) and size reflecting the r‐value. Significance levels: 0.01 ≤ *p* < 0.05 (*), 0.001 ≤ *p* < 0.01 (**), and *p* < 0.001 (***). ANA: Ancient Northeast Asian ancestry; ANEA: Ancient northern East Asian ancestry; SEA: Southern East Asian ancestry; ANE: Ancient North Eurasian ancestry; EEHG: Eastern European hunter‐gatherers. (C), Procrustes‐transformed PCA demonstrates a strong correlation between genetic differentiation and geography among 54 Turkic groups (*t*_0_ = 0.77, *p* < 1.0 × 10^−5^, rotation angle *θ* = 178.61°). Populations are grouped by geography with colors and shapes consistent with Figure 1C and Figure S3. Shaded backgrounds denote the country origins of the samples. (D), ADMIXTURE results for Turkic‐speaking populations at K = 4. (E), FineSTRUCTURE‐based topology reveals fine‐scale substructures among Turkic groups. (F–H), Total IBD sharing between the easternmost Turkic group (F), Jambyl Dungan (G), and Afghan Hazara (H) with Eurasian reference populations in the low‐density HO dataset. References include publicly available Altaic, Sino‐Tibetan (including Sinitic and Tibeto‐Burman), and Indo‐European groups. Circle positions follow the geographic distributions in Figure S14A; colors denote IBD length ranges shared with the integrated easternmost Turkic group (Dolgan and Yakut).

Admixture profile estimation on Turkic groups revealed four ancestral sources maximized in Gagauz from Eastern Europe, Tofalar in Siberia, Tubalar in southwestern Siberia, and Xunhua Salar in East Asia, respectively (Figure 2D). Haplotype‐based analyses refined this picture (Figure 2E–H and Figures S11–S15). FineSTRUCTURE clustering recovered five major groupings that closely mirrored the geographic clusters identified by PCA (Figures 1C and 2E). Within the Siberian cluster, southwestern Siberian populations (Tubalar and Shor) were distinct from southern Siberian and Russian Far Eastern Turkic groups. Identity‐by‐descent (IBD) sharing showed extensive gene flow over the past 1500 years among Turkic, Mongolic, and Tungusic groups in Siberia, revealing ancient links among distant Turkic groups (Figures S12 and S13). Longer IBD segments revealed gene flow between the easternmost Turkic groups (Dolgan and Yakut) and neighboring Turkic, Tungusic, and Mongolic groups (Figure 2F and Figure S14A–D). The overall sharing pattern still followed geographic distance decay. We detected excess IBD sharing between West Eurasian Turkic groups and geographically proximate Indo‐European groups, extending to Indo‐European groups in Central Asia and to Turkic and Tungusic groups in Siberia, a pattern primarily driven by deeper ancestral links (Figure S14E–H).

### 2.3 Long‐Distance Migration of Dungans and Hazaras Shaped Their Distinct Genetic Makeup

Dungan and Hazara deviate from the local isolation‐by‐distance pattern and provide clear examples of long‐distance migration into Central Asia. Our clustering analyses showed that neither group clustered with neighboring populations. Instead, both shared excess alleles with East Eurasian groups (Figure 1C,F and Figures S2,S3, S5–S8, and S15). Dungan shared more alleles with East Asians, especially Sino‐Tibetan speakers. Hazara displayed an admixture profile like that of Uyghurs, with additional affinity to Altaic groups in northern East Asia and Siberia. In Dungan‐related Sino‐Tibetan groups, the proportions of inferred ancestries, excluding ANEA‐, SEA‐, and Neo‐Siberian‐related, increased with latitude (0.44 ≤ *r* ≤ 0.85, *p* < 0.01), whereas SEA‐related ancestry showed a weak negative correlation (*r* = −0.31, *p* < 0.05) (Figure S16A). These ancestry components, excluding ANA‐, SEA‐, and Japanese‐related, were negatively correlated with longitude (0.42 ≤ |*r*| ≤ 0.63, *p* < 0.01), whereas SEA‐ (*r* = 0.52, *p* < 0.001) and Japanese‐related (*r* = 0.54, *p* < 0.001) components increased from West to East. In Hazara‐related Indo‐European groups, ancestries linked to East Asian, ANA, ANE, and Iranian farmer decreased with latitude (0.49 ≤ |r| ≤ 0.86, *p* < 0.05) but increased with longitude, except for ANAs (0.53 ≤ *r* ≤ 0.87, *p* < 0.05) (Figure S16B). IBD analyses showed extensive ancient connections between Dungan and East Asian populations, particularly Sino‐Tibetan groups (Figure 2G and Figure S16C,D). Hazara shared elevated IBD segments with Mongolic and Turkic groups in northern East Asia, as well as with Central Asian Turkic and Siberian Altaic groups (Figure 2H and Figure S16E,F). Together, these results show that some CAAH populations, especially Dungan and Hazara, retain genetic signatures of long‐range migration superimposed on the broader longitudinal gradient in Central Asia.

### 2.4 Admixture Modeling and Admixture Time Estimation of CAAH

We initially applied admixture *f*_3_‐statistics to identify potential source populations for CAAH populations. For most populations, *f*_3_‐statistics supported one West Eurasian and one East Eurasian source as the best‐fitting pair, consistent with admixture between deeply divergent lineages. Karluk and Dungan showed more complex patterns, suggesting distinct histories (Tables S2 and S3). To explore genetic diversity among geographically separated CAAH populations, we performed *f*_4_‐statistics of the form *f*_4_(CAAH1, CAAH2; Reference, Mbuti). Using this symmetric framework, we recovered population stratification patterns consistent with the descriptive analyses. It also showed that genetically differentiated CAAH populations shared alleles with West and East Eurasian references in distinct ways (Figure S17). Compared with other CAAH populations, Dungan showed greater genetic drift toward ancient East Asian‐ and ANA‐related ancestries, as well as toward Scythian‐related ancient Central Asians (ACAs) characterized by substantial East Asian ancestry [6]. This was followed by Kyrgyz, Karakalpak, Hazara, and Uyghur, which also showed increased allele sharing with ancient East Asians, ANAs, and ACAs with East Asian affinity. Compared with Tajiks and Turkmen, Uzbeks showed stronger genetic links to ancient East Asians and East Asian‐affinity ACAs, and greater genetic drift with ANAs and ANEAs than Karluk did. Moreover, Turkmen and Karluk shared more alleles with ancient East Asians, ANAs, and ACAs of East Asian affinity than did Tajik. Conversely, the allele‐sharing patterns between CAAH populations and Anatolian farmer‐, Iranian farmer‐, and steppe pastoralist‐related ancestors differed markedly from those involving ancient East Asians, ANAs, and East Asian‐affinity ACAs (Figure S17).

QpAdm modeling showed that most CAAH populations were best modeled as two‐way mixtures (Figure 3A). The West Eurasian source was related to Tajikistan_Ksirov_Kushan, which descended from Hajji_Firuz_C, Bactria Margiana Archaeological Complex, and late Bronze Age steppe lineages, and contributed 30%–86% of ancestry. The East Eurasian source was related to Xianbei_IA from the Amur region and contributed 14%–70% of ancestry. We modeled Dungan as predominantly ANEA‐related (∼90%) with minor Tajikistan_Ksirov_Kushan ancestry, whereas we modeled Karakalpak as a mixture of Alan (∼42%) and Xianbei_IA‐related steppe pastoralist lineages. Sourcefind and fastGLOBETROTTER analyses supported these models at the haplotype level and identified French, Kalash, and Bedouin‐like sources for West Eurasian ancestry and Yakut and northern Han‐like sources for East Eurasian ancestry (Figure S18). We identified a single admixture event for most CAAH populations, except Tajik, which experienced multiple events (Figure 3B and Figure S19). West Eurasian ancestry, linked to French ancestry, made up 32%–45% of the Kyrgyz, Karakalpak, Hazara, and Uyghur genomes and over 50% of the Turkmen, Uzbek, and Karluk genomes (57%–62%). The remaining genetic component was derived mainly from Yakut, indicating East Eurasian influence. Dungan was modeled as approximately 87% Northern Han and 13% French. Tajik ancestry appeared to involve two distinct admixture events, with varying contributions from French, Bedouin, Kalash, Northern Han, and Yakut populations (Figure S19B).

**FIGURE 3:**
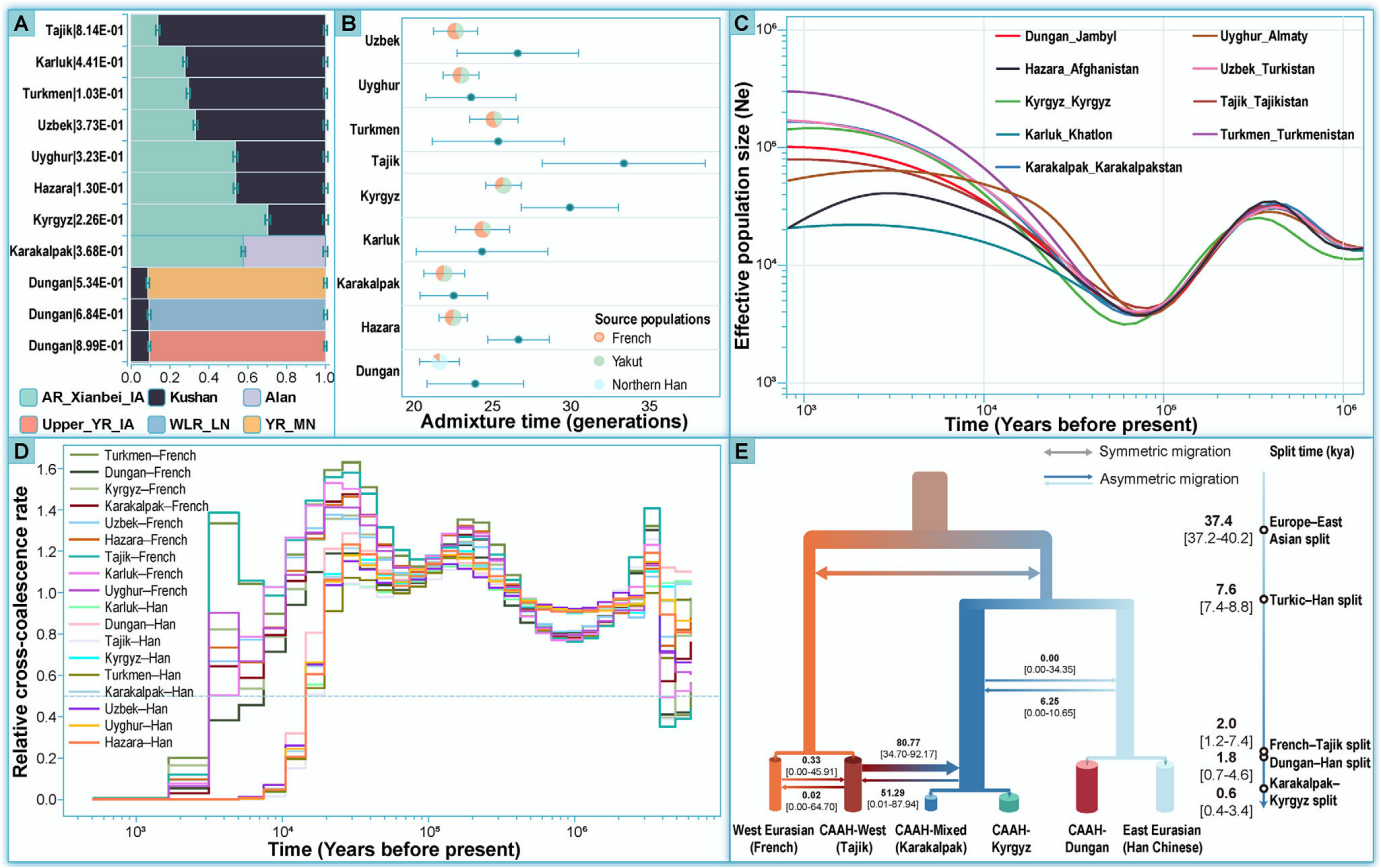
Admixture profiles, admixture dates, and demographic modeling of CAAH populations. (A), Admixture proportions of each CAAH population based on qpAdm estimation. The numerical value following each population represents the *p*‐value from the qpAdm analysis. A *p*‐value > 0.05 indicates that the target population can be successfully modeled as a mixture of the specified source populations. (B), The admixture proportions were inferred from fastGLOBETROTTER analysis, and admixture times were inferred from fastGLOBETROTTER and MALDER analyses. The blue circles denote the MALDER‐based admixture times. Other colored circles denote the fastGLOBETROTTER‐based admixture times and ancestry compositions. The color of the circle represents the different surrogate ancestral sources. The number of admixtures is presented as the mean ± standard deviation. (C), Effective population sizes of each CAAH population. (D), Population divergence times between each CAAH population and East/West Eurasian source populations. EE: East Eurasian population, WE: West Eurasian population. (E), Maximum‐likelihood demographic model of Central Asian populations. Brackets indicate 95% confidence intervals obtained by non‐parametric block bootstrap. Migration rates scaled by effective population size (2Nm) are shown above or below the corresponding arrows; arrows representing gene flow with 2Nm > 1 are bolded.

We used MALDER and fastGLOBETROTTER to date east–west admixture. MALDER‐based estimates revealed a single admixture event involving a West Eurasian source (French) and an East Eurasian source (Yakut or Northern Han). We dated this event to approximately 23–34 generations ago (Figure 3B). FastGLOBETROTTER produced concordant dates, placing admixture in most CAAH populations at ∼22 to 25 generations ago. More recent admixture events were suggested for Uzbek, Hazara, Kyrgyz, and Dungan, which were estimated to have occurred approximately 22–26 generations ago (Figure 3B). In Tajik, two distinct admixture pulses were identified: the most recent approximately 9 generations ago, and an older event approximately 50 generations ago. Demographic inference with SMC++ revealed that CAAH populations experienced substantial growth over the past 10000 years, with lineage‐specific (Karluk, Uyghur, and Hazara) peaks and declines (Figure 3C). Divergence times were ∼20.8–15.3 kya between East Eurasians (Northern Han) and CAAH, ∼7.2 to 2.6 kya between West Eurasians (French) and CAAH, and ∼3.3–1.9 kya among CAAH populations (Figure 3D and Figure S20A). The proportion of West Eurasian ancestry was positively correlated with divergence time from East Eurasians and negatively correlated with divergence time from West Eurasians (Figure S20B,C). We reconstructed demographic histories for four representative CAAH lineages using coalescent simulations. Among models allowing alternative origins and either symmetric or asymmetric gene flow, the best‐fitting model placed the divergence between East and West Eurasian lineages at ∼37.4 kya [95% confidence interval (CI), 37.2–40.2 kya] (Figure 3E and Figure S21). Turkic‐speaking Karakalpak and Kyrgyz shared a common ancestor with Dungan and Han Chinese, diverging at ∼7.6 kya (95% CI: 7.4–8.8 kya). The Karakalpak–Kyrgyz lineage experienced significant gene flow from the Tajik‐related lineage. Divergence between French and Tajik occurred ∼2.0 kya (95% CI: 1.2–7.4 kya), between Dungan and Han Chinese ∼1.8 kya (95% CI: 0.7–4.6 kya), and between Karakalpak and Kyrgyz ∼0.6 kya (95% CI: 0.4–3.4 kya).

### 2.5 Sex‐Biased Admixture Landscape

To investigate sex‐biased admixture in CAAH populations, we estimated and compared the proportions of source populations via autosomal, X‐chromosomal, Y‐chromosomal, and mitochondrial data. Differences in the proportions of autosomal and X‐chromosomal ancestry can indicate sex‐biased admixture. The western Eurasian genetic contribution ranged from 7.8% to 86.4% for autosomes and from 8.4% to 85.8% for X chromosomes (Figure S22). We detected a substantial female bias toward West Eurasian ancestry in Hazara (Z = −0.43; Student's *t*‐test, *p* < 0.05), which is consistent with an ∼71.4% enrichment of West Eurasian‐related maternal lineages, such as haplogroups H, J, K, N1, T, U, and W (Table S4). We found no notable sex bias in the other CAAH populations (−0.20< Z < 0.13; Student's *t*‐test, *p* > 0.05). However, several groups showed discordance between West Eurasian paternal or maternal lineages and their autosomal or X‐chromosomal ancestry estimates (Figure S22 and Table S4). All Karluk individuals carried the Y‐chromosome haplogroup L1a2a, which is common in South Asian populations. West Eurasian‐related paternal lineages appeared in 65.0% of Kyrgyz, 35.3% of Karakalpak, and 30.0% of Dungan, whereas West Eurasian maternal lineages were present in 88.9% of Karluk, 70.0% of Tajik, 55.0% of Uzbek, and 31.6% of Uyghur (Table S4). Overall, we found no evidence for a consistent excess of West Eurasian paternal lineages together with East Eurasian maternal lineages, or the reverse, across CAAH populations.

### 2.6 Genomic Signatures of Positive Selection

Local adaptation can shift allele frequencies beyond expectations under admixture alone [51, 52]. We used multiple selection detection strategies to reconstruct the selection signatures of Central Asians. To identify post‐admixture signals and evaluate their potential biological functions, we first quantified deviations between observed and expected allele frequencies (AFd_e_) in four genetically inferred subgroups: CAAH‐West, CAAH‐Mixed, CAAH‐Kyrgyz, and CAAH‐Dungan (Figure S23). Functional enrichment analysis revealed a shared overrepresentation of neural functions across all four subgroups. In contrast, subgroup‐specific signals diverged markedly: CAAH‐West was enriched for synaptic and neurodevelopmental pathways, CAAH‐Mixed for transport and structural development, CAAH‐Kyrgyz for cell adhesion and tissue organization, and CAAH‐Dungan for behavioral and regulatory processes (Figure S24A). Among metabolic candidates, *EBF2* showed strong signals, particularly in CAAH‐West and CAAH‐Mixed (Figure 4A,B). *EBF2* regulates adipogenesis, and several 3′UTR variants, including rs13257257‐G and rs17054430‐C, occurred at substantially higher observed frequencies (AF_obs_: 0.34–0.42) than expected under the admixture model (0.16–0.18). These variants have been linked to altered brown adipogenesis, increased white fat storage, and susceptibility to inguinal hernia [53]. In CAAH‐West, *FNDC1*, involved in cellular responses to hypoxia and positive regulation of cardiomyocyte apoptosis, also showed pronounced AFd_e_. This signal included rs294883‐T, which has been associated with coronary artery disease (Figure 4A) [54]. In CAAH‐Mixed, two *CARS2* variants, rs7324648‐A and rs373373‐C, were strongly enriched (Figure 4B). These variants influence the synthesis of enzymes involved in serine synthesis [55, 56]. *MTAP*, a key regulator of polyamine metabolism, also showed strong signals in this subgroup (Figure 4B and Table S5). Notably, rs871024, which is linked to melanoma, was more common in Hazara and South Asian populations. Its derived allele frequency increased during the transition from hunting‐gathering to farming and later stabilized during the nomadic period (Figure S24B–D). In CAAH‐Kyrgyz, *CLDN10* emerged as a prominent candidate, with rs77008184, rs2095774, and rs17189719 showing marked differentiation (Figure 4C). These variants are relevant to renal function and intestinal barrier integrity and are associated with serum uric acid and cystatin C levels [55, 56]. The derived allele frequencies of rs77008184 and rs17189719 declined during the shift from hunting‐gathering to farming and fell further with the expansion of pastoralism, whereas rs2095774 followed the opposite trajectory (Figure S25A–C). In CAAH‐Dungan, clusters of variants in *LINGO2*, previously linked to body mass index and fat distribution, displayed extended haplotype homozygosity (EHH) patterns indicative of local adaptation (Figure 4D,E and Figures S25D, S26).

**FIGURE 4:**
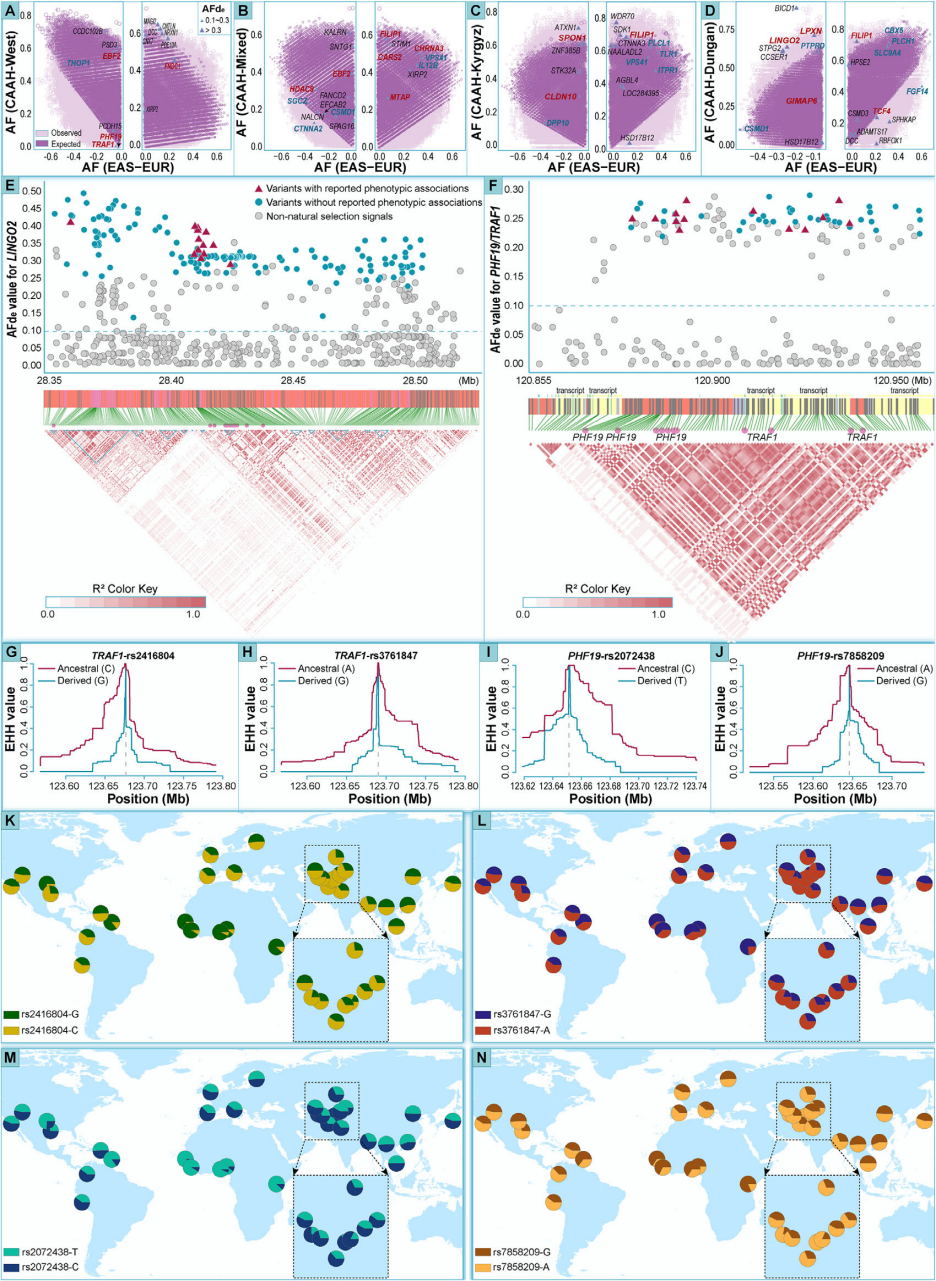
Signatures of biological adaptation in genetically diverse CAAH subgroups and frequency distributions of specific variants. (A–D), Observed and expected allele frequency (AF_obs_ and AF_exp_) distributions for variants within each CAAH subgroup, shown in the context of frequency differences between East and West Eurasian ancestral populations. "Observed" represents the empirical allele frequency in each CAAH subgroup, and "Expected" represents the allele frequency predicted under the fitted admixture model. The top 0.1% of genes per subgroup identified by the window‐based approach that lack reported phenotypic associations in the GWAS Catalog are shown in black; well‐studied genes with GWAS Catalog annotations are shown in red; and genes identified by the highly differentiated variant (HDV) screening‐based approach are shown in scuba blue. AF: allele frequency. (E–F), Deviations between AF_obs_ and AF_exp_ (AFd_e_) as well as linkage patterns of variants in the *LINGO2* (E) and *TRAF1*/*PHF19* genes (F). The colored markers indicate natural selection signatures on these genes. Colored bars beneath each selection signal indicate local genomic features: yellow, coding sequence; light blue, intronic region; pink, untranslated region; orange, intergenic region. (G–J), Extended haplotype homozygosity (EHH) curves for *TRAF1*‐rs2416804 (G), *TRAF1*‐rs3761847 (H), *PHF19*‐rs2072438 (I), and *PHF19*‐rs7858209 (J). (K–N), Allele frequency distributions of *TRAF1*‐rs2416804 (K), *TRAF1*‐rs3761847 (L), *PHF19*‐rs2072438 (M), and *PHF19*‐rs7858209 (N).

We also detected strong immune‐related post‐admixture signals. In CAAH‐West, these centered on *TRAF1* and *PHF19* (Figure 4F, Figure S27A, and Table S5). These loci included alleles linked to allergic diseases (*PHF19*‐rs10760123/rs6478484/rs10818483 and *TRAF1*‐rs1930781), rheumatoid arthritis (RA; *TRAF1*‐rs10435844/rs3761847, *PHF19*‐rs1953126/rs881375/rs2072438), white blood cell count (*TRAF1*‐rs2416804), and lymphocyte count (*PHF19*‐rs7858209) based on previous genome‐wide association studies (GWAS) and medical findings [57, 58, 59, 60]. The frequencies of *TRAF1*‐rs2416804/rs10435844/rs3761847 and *PHF19*‐rs2072438/rs7858209 declined before the agricultural era and again after the rise of nomadic pastoralism, whereas other loci showed opposite trends (Figure S27B–L). EHH decay supported strong positive selection on ancestral alleles at these loci and explained substantial differences in allele frequencies among CAAH subgroups and across continental populations (Figure 4G–N). Analysis of derived allele frequencies in spatiotemporally distributed ancient Eurasian genomes further showed that these loci shifted in frequency during the transitions to agriculture and nomadic pastoralism (Figure S27M) [61]. In CAAH‐Dungan, immune‐related signals involved *LPXN*, *PPP4R1L*, and *GIMAP6*, including *LPXN*‐rs10896794, linked to inflammatory bowel disease; *PPP4R1L*‐rs480143, associated with white blood cell count; and *GIMAP6*‐rs62491814, related to C‐reactive protein levels (Figure 4D and Table S5) [57, 62, 63].

We also observed enrichment of selection signals at loci affecting neurodevelopmental traits. The variant rs4708181 in *FILIP1* was identified in CAAH‐Mixed, CAAH‐Kyrgyz, and CAAH‐Dungan (Figure 4B–D) and has been linked to cortical surface area [64]. In CAAH‐Mixed, we observed strong selection signals in *CHRNA3*, with rs112878080 linked to carbon monoxide diffusing capacity and nine additional variants associated with pulmonary traits, including the FEV1/FVC ratio and post‐bronchodilator FEV1 (Figure 4B and Table S5) [65, 66]; notably, derived allele frequencies of rs112878080, rs138544659, and rs8040868 declined ∼5500 years ago (Figure S28A–C). Selection signals also clustered in *HDAC9*, a gene implicated in brain morphology and neurological conditions (Figure S28D). Variants associated with cortical surface area, cortical thickness, whole‐brain restricted diffusion, and male‐pattern baldness [67, 68, 69, 70], except rs13242758 and rs2073963, showed dynamic trajectories between about 9 and 6 kya and then stabilized after the regional transition to nomadic pastoralism (Figure S28E–O). In CAAH‐Kyrgyz, *SPON1*, which encodes an axon‐guidance protein, carried rs12575169, associated with adolescent idiopathic scoliosis [71], and rs2618516, associated with brain connectivity [72]. In CAAH‐Dungan, *TCF4*, a key regulator of neural development, showed strong selection signals (Figure 4D and Table S5). Variants linked to neuroticism, risk‐taking, and mood‐related traits [73, 74], including rs2919451 and rs2924328, increased sharply in derived allele frequency between about 9 and 7.5 kya, increased again from about 5–3 kya, and then declined markedly around 3 kya (Figure S28P,Q).

To further refine post‐admixture adaptive candidates, we intersected highly differentiated variants (HDVs) between East and West Eurasians with variants showing substantial AFd_e_, yielding 3 69 407 candidates. We then classified variants with AFd_e_ > 0.1 in individual CAAH subgroups as potentially adaptive (Table S6). In CAAH‐West, *THOP1*, a locus associated with testosterone and low‐density lipoprotein cholesterol levels, showed a strong signal (Figure 4A). EHH curves supported local adaptation at these variants (Figure S29A–E). In CAAH‐Mixed and CAAH‐Kyrgyz, *VPS41* showed notable signals, with rs6943746 and rs10274968 linked to neuroticism and depression (Table S6). Although most adaptive signals in CAAH‐Mixed and CAAH‐Kyrgyz showed a West Eurasian frequency bias, HDVs with elevated AFd_e_ in CAAH‐Kyrgyz exhibited an East Eurasian shift (Figure 5A,B). The allele frequency of *VPS41*‐rs10274968 rose markedly between 9 and 7 kya, increased slightly thereafter, and stabilized ∼4 kya (Figure 5C). Additional adaptive signals affected genes related to nervous system function, including *CTNNA2*, associated with chronic pain, in CAAH‐Mixed; *PLCL1* and *DPP10*, associated with psychiatric disorders, in CAAH‐Kyrgyz; and *PLCH1*, *PTPRD*, and *FGF14*, associated with insomnia, in CAAH‐Dungan (Figure 4B–D and Table S6) [75, 76]. Immune‐related loci were also strongly differentiated. These included *CSMD1*‐rs1529316, associated with multiple sclerosis, in CAAH‐Mixed and CAAH‐Dungan; *SGCZ*‐rs9886428, associated with IgG glycosylation, and *IL12B*‐rs3313094, associated with psoriasis, in CAAH‐Mixed; *TLR1*‐rs5743614/rs6531663, associated with atopic dermatitis and allergic disease, in CAAH‐Kyrgyz; and *SLC9A4*‐rs1468788, associated with celiac disease, in CAAH‐Dungan. *IL12B*‐rs3313094 increased in frequency with the transition to agriculture but declined before the rise of nomadic pastoralism (Figure S29F). *TLR1*‐rs5743614 decreased after ∼6 kya, whereas *TLR* *1*‐rs6531663 increased steadily over the same period (Figure S29G,H). Notably, the allele frequency at *SLC9A4*‐rs1468788 rose continuously over the past 10 kya (Figure 5D). These immune‐related loci also showed pronounced geographic variation in allele frequency (Figure S30). We further identified a diet‐related signal at *CBX5*‐rs4759074 in CAAH‐Dungan, potentially consistent with adaptation to meat‐based diets. Its frequency peaked ∼6 kya, before the emergence of pastoralism (Figure 5E). Population‐specific enrichment also involved *CBX5*‐rs17110109 in CAAH‐Dungan and *ITPR1*‐rs13313995 in CAAH‐Kyrgyz, both linked to alopecia (Table S6) [70, 77, 78]. These *CBX5* variants showed marked frequency differences between CAAH‐Dungan and other CAAH subgroups, as well as across continental populations (Figure 5F,G).

**FIGURE 5:**
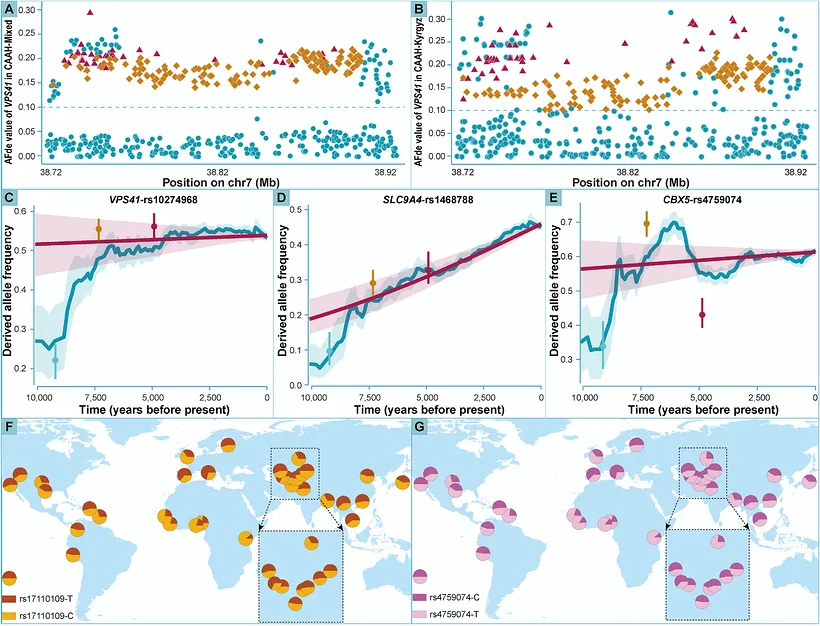
AFd_e_ distribution of *VPS41* variants, allele frequency trajectories, and distributions of local adaptive signatures. (A–B), AFd_e_ distribution of *VPS41* variants in CAAH‐Mixed (A) and CAAH‐Kyrgyz (B). Loci marked in orange‐yellow represent HDVs within *VPS41*, exhibiting a West Eurasian‐biased frequency in CAAH‐Mixed or CAAH‐Kyrgyz. The claret‐marked loci indicate HDVs with East Eurasian‐biased frequencies in CAAH‐Mixed or CAAH‐Kyrgyz. Loci marked in turquoise denote variants within the *VPS41* gene that lack signals of natural selection. (C–E), Frequency trajectories for *VPS41*‐rs10274968 (C), *SLC9A4*‐rs1468788 (D), and *CBX5*‐rs4759074 (E). (F–G), Allele frequency distributions of *CBX5*‐rs17110109 (F) and *CBX5*‐rs4759074 (G) across global populations.

We next performed a genome‐wide scan for strong local adaptation using the composite of multiple signals (CMS) to validate these reported signatures and explore method‐specific recent or old selection signatures. We identified 363 significant gene regions in CAAH‐West, 333 in CAAH‐Mixed, 484 in CAAH‐Kyrgyz, and 428 in CAAH‐Dungan (Table S7). CMS confirmed multiple candidate regions identified by the window‐based AFd_e_ scan, including *PSD3*, *SNX7*, and *XIRP2* in CAAH‐West; *FANCD2* and *XIRP2* in CAAH‐Mixed; *CTNNA3*, *HSD17B12*, *NAALADL2*, *SPON1*, and *ZNF385B* in CAAH‐Kyrgyz; and *ADAMTS17*, *BICD1*, *CSMD3*, *HPSE2*, *PTPRD*, and *TCF4* in CAAH‐Dungan (Tables S5 and S7). Across all four groups, enrichment analyses consistently highlighted glycosylation‐related pathways, including O‐linked glycosylation and diseases of glycosylation (Figure S31). Neuronal and signaling‐related processes, including synaptic signaling and neuronal system pathways, also recurred across multiple groups. At the subgroup level, CAAH‐West showed moderate enrichment for neuronal and circadian pathways, CAAH‐Mixed displayed fewer and more restricted signals, CAAH‐Kyrgyz showed broader enrichment in membrane transport and receptor‐mediated signaling, and CAAH‐Dungan showed stronger enrichment in cell morphogenesis and projection assembly. In addition, CMS identified several strong signals that were not prominent in the window‐based scan. In CAAH‐West and CAAH‐Mixed, these included variants in *MPP5* and *COL21A1*, both potentially involved in vessel‐wall integrity and extracellular matrix remodeling, as well as *SMAD5*, which functions in angiogenesis, tissue homeostasis, and osteochondral differentiation, all of which showed clear CMS peaks (Table S7). We also identified a shared signal in *PRIM2*, which encodes a subunit of the DNA primase complex required for replication initiation, suggesting recent positive selection in CAAH‐Kyrgyz and CAAH‐Dungan. In CAAH‐West, *ECD*, *KLHL24*, and *CCDC25* harbored 56, 45, and 43 candidate loci, respectively. Previous studies linked rs2271904 and rs36152134 in *ECD* to blood pressure, rs10110224 in *CCDC25* to insomnia, and rs11782624 in *CCDC25* to giant cell arteritis (Figure S32). In CAAH‐Mixed, we detected a strong signal in the *HLA‐DPA1*/*HLA‐DPB1* region, which is primarily associated with immune‐related phenotypes (Figure S33). In CAAH‐Kyrgyz, *IPMK* contained 25 candidate loci, including rs2790216 and rs7922032, which have been associated with immune‐related traits, such as inflammatory bowel disease and variation in monocyte count (Figure S34). In CAAH‐Dungan, we observed strong CMS peaks at *ULK4* and *ANKRD31*, with rs7707394 in *ANKRD31* previously associated with low‐density lipoprotein levels (Figure S35). Our work presented a complex selection landscape among Central Asians, which influenced the health of Central Asian populations through adaptation and antagonistic pleiotropy among immune, metabolic traits, and disease.

### 2.7 Neanderthal and Denisovan Introgression and the Biological Functions of Archaic Heritage

Archaic introgression varies across Eurasian and Oceanian populations [79, 80], but its extent in Central Asia remains incompletely characterized. We used high‐efficiency methods, including Sprime and IBDmix, to reconstruct archaic introgression patterns in Central Asians. We first used IBDmix to identify archaic introgression segments (AISs) in the Altai Neanderthal genome, following the same rules and thresholds as in recent work [79, 80, 81]. The distribution of Neanderthal‐derived sequences in CAAH‐West closely resembled that of European populations from the 1000 Genomes Project (1KGP), whereas patterns in CAAH‐Mixed mirrored South Asians (Figure 6A). In contrast, CAAH‐Kyrgyz and CAAH‐Dungan showed the shortest Neanderthal AISs per individual, which may partly reflect differences in sample size [81]. On average, each carried ∼50.01 Mb of Neanderthal‐derived sequences, ranging from 52.95 Mb in CAAH‐West and CAAH‐Mixed to 39.23 Mb in CAAH‐Kyrgyz. We identified 104 Neanderthal‐introgressed regions absent from the 1KGP populations, as Central Asian populations were not included in this project (Figure S36A). These included numerous archaic segments and genes unique to CAAH populations, such as *GUSBP1* and *BCL11A*, indicating additional regional diversity in archaic ancestry. For example, one introgressed segment overlapping *BCL11A* is associated with hematopoietic cell development and hematological disorders. Additionally, 54 segments were specific to CAAH subgroups: 27 in CAAH‐West, 18 in CAAH‐Mixed, 4 in CAAH‐Kyrgyz, and 5 in CAAH‐Dungan (Figure S36A). To increase confidence, we also applied Sprime [82] to infer Neanderthal‐ and Denisovan‐derived segments and integrated Neanderthal calls across methods to obtain the high‐confidence introgressed genomic regions and genes. This yielded 1605 high‐confidence Neanderthal segments spanning 392.51 Mb and 132 Denisovan segments covering 37.17 Mb (Figure S36B). On average, each carried 13.24 Mb of high‐confidence Neanderthal sequence and 0.34 Mb of Denisovan sequence (Figure 6B and Figure S36B). Our data supported a model involving one major Neanderthal admixture pulse and two Denisovan‐related pulses, based on patterns of match rates, consistent with those reported in East Asian populations [80] (Figure 6C, Figure S37, and Table S8).

**FIGURE 6:**
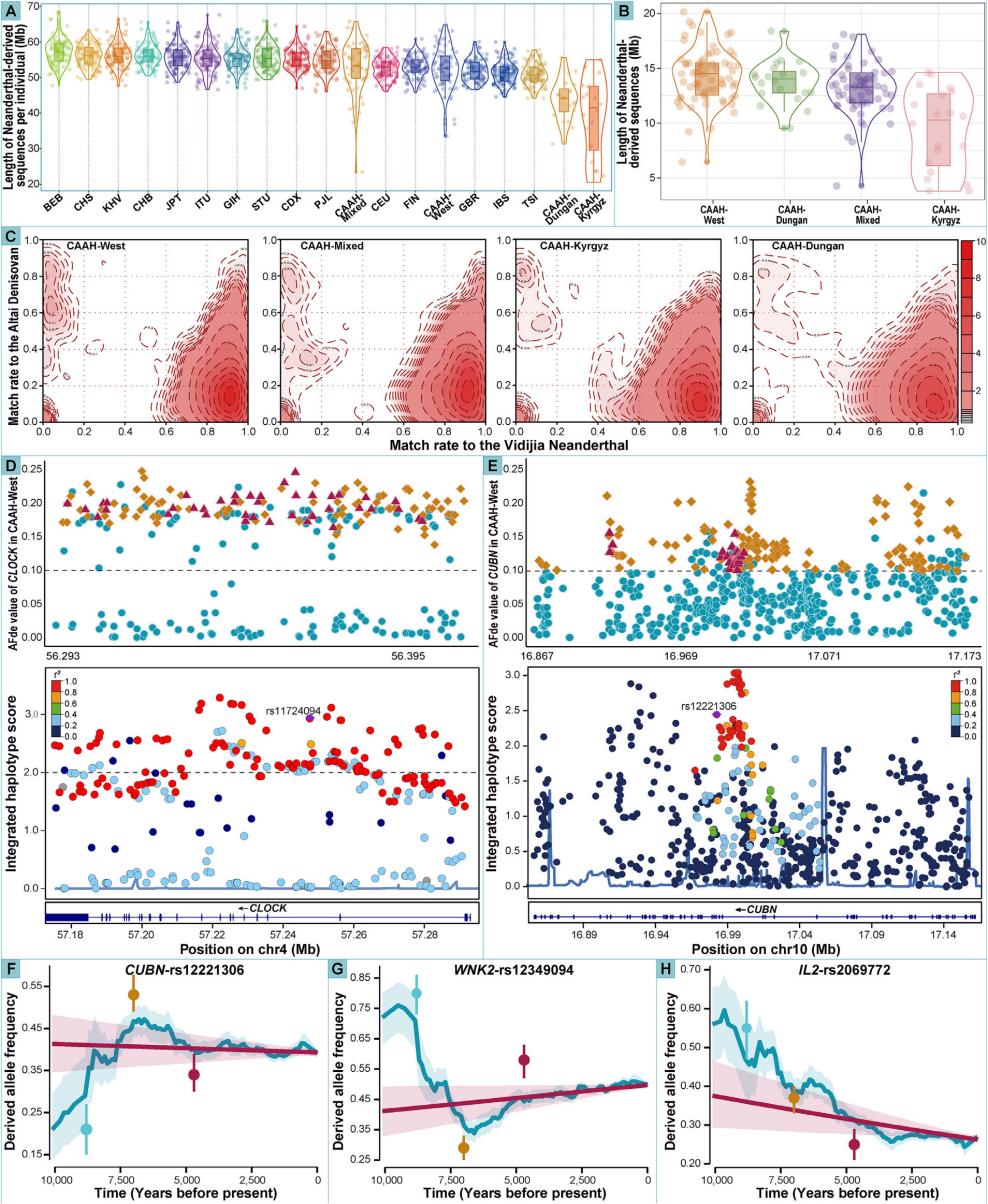
Distribution of Neanderthal‐ and Denisovan‐derived archaic introgression segments (AISs) and adaptive introgression signals. (A), Distribution of Neanderthal‐derived AISs identified via the IBDmix method across four CAAH subgroups and Eurasian reference populations from the 1000 Genomes Project (1KGP). (B), Distribution of high‐confidence Neanderthal‐inherited AISs within each CAAH subgroup, as determined by IBDmix and Sprime. (C), Contour density plots of the matching proportions of introgressed fragments to the Neanderthal and Denisovan genomes. (D), Distribution of AFd_e_ and integrated haplotype score (iHS) for potential Neanderthal‐introgressed variants in the *CLOCK* gene identified in CAAH‐West. The upper panel shows the AFd_e_ distribution. Claret triangles indicate adaptive introgression signals, orange‐yellow rhombi represent natural selection signatures, and turquoise circles denote non‐adaptive introgression and non‐natural selection signals in the *CLOCK* gene. The lower panel depicts iHS values and linkage patterns within the target region centered on rs11724094, with variant positions corresponding to those in the upper panel. (E), Distribution of AFd_e_ and iHS values ​​for potential Neanderthal‐introgressed variants in the *CUBN* gene identified in CAAH‐West, along with linkage patterns within the target region centered on rs12221306. Variant color coding is consistent with Figure 6D. (F–H), Frequency trajectories over time for *CUBN*‐rs12221306 (F), *WNK2*‐rs12349094 (G), and *IL2*‐rs2069772 (H).

We next explored the potential phenotypic relevance of high‐confidence AISs using pathway enrichment and GWAS annotations. We identified multiple previously unreported Neanderthal signals at immunity‐related loci, including *CXCR5*, *TLR5*, *PLCL2*, *RAB17*, *USP20*, *TRIML1*, *PIBF1*, *C6*, and *HHLA1*, and at metabolism‐related loci, including *DDC*, *FAH*, *MTRR*, *ASRGL1*, *ACSBG2*, *ALK*, and *CRTC3*. We also replicated many previously reported Neanderthal‐associated regions linked to immune, metabolic, pigmentation, dermatological, neurological, and reproductive traits [83, 84, 85, 86] (Table S9). Denisovan segments similarly overlapped loci previously associated with immune and metabolic phenotypes (Table S9). Within the CAAH subgroups, Neanderthal segments at *BUD13*, *ZPR1*, and *APOA5* overlapped loci associated with lipid metabolites. CAAH‐specific Neanderthal sequences with phenotypic relevance included *L3MBTL3* (lipid metabolism), *RAD51B* (RA/asthma), and *ESR1* (bone mineral density) in CAAH‐West [87, 88]; *PPP6R3* (bone mineral density) and *MAD1L1* (psychiatric disorders) in CAAH‐Mixed [89, 90]; *SLCO1B1* (hepatic drug transport) in CAAH‐Kyrgyz; and *UBE2L3* (serum lipids and autoimmune disorders, such as Crohn's disease, inflammatory bowel disease, and systemic lupus erythematosus) in CAAH‐Dungan [91]. Denisovan‐derived segments overlapped loci previously associated with height, type 2 diabetes, and Takayasu arteritis; notable variants included *HHAT* rs115453328 in CAAH‐West (obsessive–compulsive traits), *RASA3* rs61971965 in CAAH‐Kyrgyz (gray matter density), and *IL2RA* rs61839660 in CAAH‐Dungan (immune‐related traits). Pathway analysis further revealed shared enrichment for muscle cell differentiation, nervous system development, and muscle tissue growth (Figure S38).

Although archaic adaptive introgression has been reported in other regions, its role in Central Asia remains unclear [17, 79, 80]. We identified a Neanderthal‐derived haplotype at *CLOCK* comprising 42 archaic SNPs in CAAH‐West, with rs11724094 linked to male‐pattern baldness (Figure 6D). We also detected additional archaic signals in CAAH‐West, including *CUBN* rs12221306, associated with metabolite levels, and *WNK2* rs12349094, associated with educational attainment. Both loci showed distinct temporal frequency trajectories, consistent with changing evolutionary pressures over time (Figure 6E–G). In addition, we detected archaic SNPs at *PXK* rs6445975, linked to systemic lupus erythematosus, *IL2* rs2069772, linked to allergic sensitization, and *CDC42BPA* rs1929863, linked to white blood cell count. Notably, *IL2* rs2069772 showed a marked decline in derived allele frequency since ∼10 kya (Figure 6H) [92]. In CAAH‐Kyrgyz, *DGKH* rs73187291 was associated with insulin‐like growth factor 1 levels [55], and in CAAH‐Dungan, *TOR1B* rs12634, located in the 3′ UTR, was linked to prostate cancer risk. Collectively, these findings show that archaic introgression patterns in CAAH genomes differentiated from those of other populations and may have contributed, at least in part, to local adaptation in metabolic and immune phenotypes.

### 2.8 Medical Relevance and Pharmacogenomic Implications

To characterize the distribution of medically relevant variants in CAAH populations, we annotated all variants against ClinVar [93] and identified 116 pathogenic (level 5) variants across 99 genes. Every participant carried at least two ClinVar‐listed pathogenic variants (median, 9 alleles; range, 3–16; Figure 7A). Among the unique pathogenic variants, 56.9% (66/116) were singletons, 16.4% had a MAF < 0.01, and 11.2% had a MAF of 0.01–0.05 (Figure 7A). Among the 208 pathogenic or likely pathogenic (level 4) variants, 17.8% (37/208) had a MAF > 0.05; three of these had a mean MAF < 0.05 in the Genome Aggregation Database (gnomAD) v3.1.2, and only one had a MAF < 0.05 across all gnomAD populations (Figure 7B and Table S10). Most variants (∼51.9%) were linked to autosomal recessive disorders, whereas ∼20.7% were linked to autosomal dominant disorders (Figure 7B and Figure S39A). To assess the representation of reportable secondary findings, we cross‐referenced pathogenic and likely pathogenic variants with the American College of Medical Genetics and Genomics (ACMG) Secondary Findings Panel (ACMG‐SF v3.2) [94]. Ninety‐two of 166 individuals carried at least one of 10 ACMG‐listed variants spanning seven genes (Figure 7C). Four variants occurred as singletons, in *MUTYH* and *BTD* (autosomal recessive) and in *LDLR* (autosomal dominant), and two appeared as doubletons in *BRCA2* (autosomal dominant) and *ATP7B* (autosomal recessive). Notably, *SCN5A A1673G* (rs1805124, H558R), associated with autosomal dominant ventricular fibrillation and altered cardiac sodium channel function, was present in 65 individuals (∼39.2%; Figure 7B). This variant was most frequent in Karakalpak (∼41.2%) and was also common in African populations (Figure S39B), suggesting that its interpretation may be sensitive to ancestry context and that some classifications in ACMG and related resources may warrant cautious re‐evaluation in population datasets.

**FIGURE 7:**
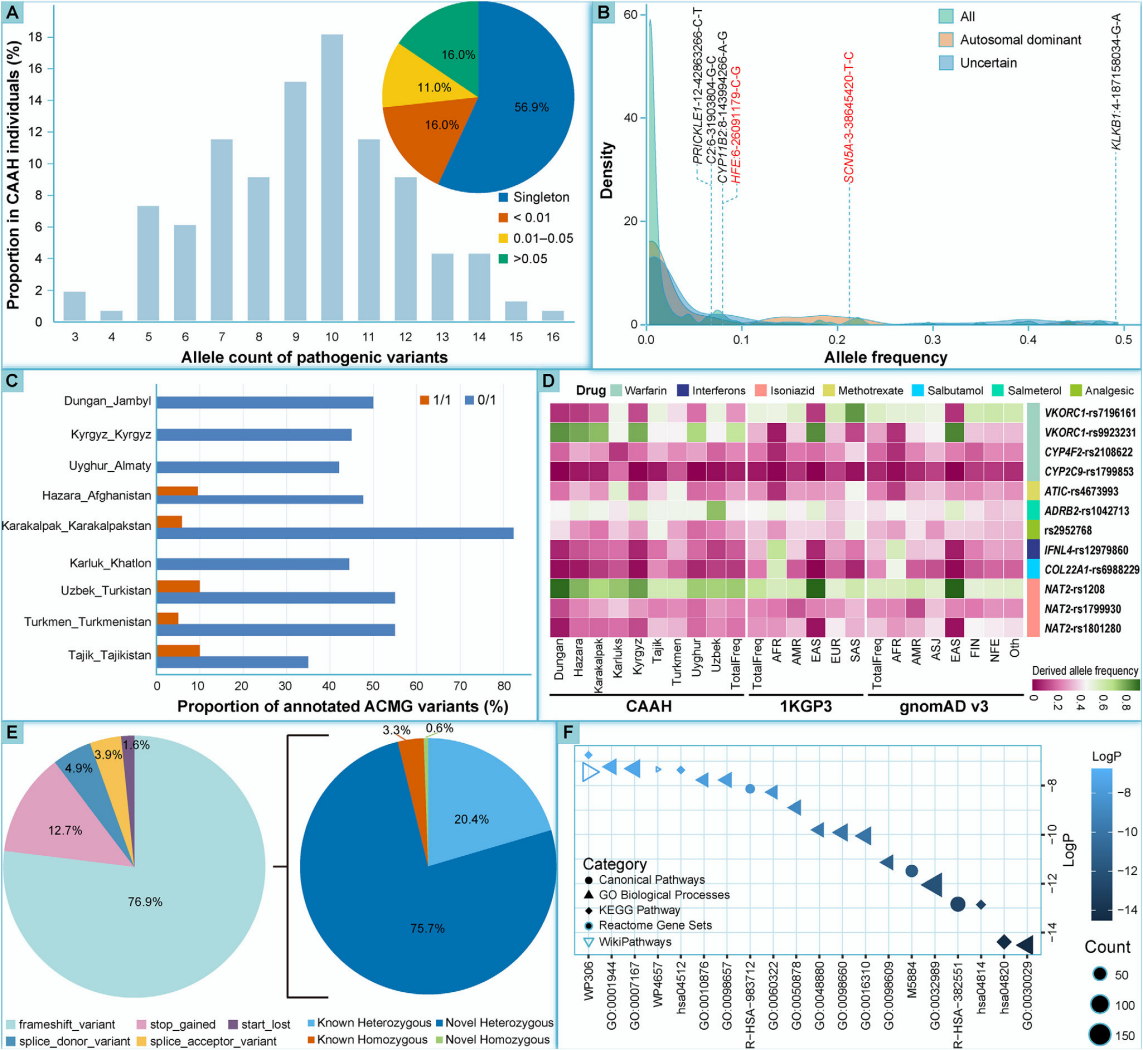
Statistics of medically relevant genetic variants in our dataset. (A), The number of alleles of identified pathogenic variants carried by each CAAH individual, alongside the allele frequency distribution of these variants. (B), Density plot displaying the frequencies of pathogenic and likely pathogenic ClinVar variants (*n* = 208), categorized by the most common inheritance patterns of associated monogenic disease genes; variants with autosomal dominant inheritance or unknown modes are highlighted. The variant with the highest minor allele frequency (MAF) value, three variants with MAFs > 0.05 in CAAH but average MAFs < 0.05 in the Genome Aggregation Database (gnomAD v3.1.2), and two reportable American College of Medical Genetics and Genomics (ACMG) variants with MAFs > 0.05 are presented as gene names: chromosome‐base pair position‐reference allele‐alternative allele. (C), Distribution of homozygous (1/1) and heterozygous (0/1) genotypes for annotated ACMG variants across each CAAH population. (D), Frequency spectra of derived alleles for pharmacogenomic variants in our dataset, the 1KGP, and gnomAD. AFR: African, AMR: American, EAS: East Asian, EUR: European, SAS: South Asian, ASJ: Ashkenazi Jewish, FIN: Finnish, NFE: non‐Finnish European, and Oth: Remaining individuals. (E), Functional annotations of putative loss‐of‐function variants and the proportions of previously unreported and known heterozygous and homozygous protein‐truncating variants (PTVs) in our dataset. (F), The enrichment results of annotated heterozygous PTVs.

We next examined allele frequencies for pharmacogenomic variants from the ADME (absorption, distribution, metabolism, and excretion) gene list and ClinVar drug‐response variants, revealing marked differences between CAAH and other continental populations (Figure 7D). For warfarin, the derived allele of *VKORC1*‐1639A (rs9923231) was frequent (≥ 73.7%) in Dungan, Hazara, Karakalpak, Kyrgyz, and Uyghur, consistent with prior reports that this allele is associated with lower warfarin dose requirements in some populations. *VKORC1* rs7196161 showed elevated derived allele frequencies (≥42.5%) in Karluk, Tajik, Turkmen, Uzbek, and non‐East Asian reference populations, particularly South Asians. CYP4F2*3 (rs2108622), previously associated with higher warfarin dose requirements, varied substantially across CAAH populations, as did CYP2C9*2 (rs1799853), which was enriched in Karluk and Turkmen. CYP2C9*2 carriers exhibit reduced warfarin metabolism, increased phenytoin sensitivity, and a greater risk of gastrointestinal bleeding with some non‐steroidal anti‐inflammatory drugs. Together, these patterns support genotype‐informed warfarin dosing in Central Asia, although direct clinical utility would require independent pharmacokinetic and outcome validation in the relevant populations. We also observed pronounced frequency variation for *ATIC* T675C (rs4673993), with high frequencies (≥ 40%) in Karluk, Tajik, Turkmen, Dai in China, and South Asian references (Figure 7D and Figure S39C); CC homozygotes presented an enhanced response to methotrexate, underscoring the value of *ATIC* genotyping. Uzbek exhibited a very high derived allele frequency at *ADRB2* rs1042713 (77.5%), which has been reported to be associated with salmeterol efficacy. Additional frequency differences were detected in loci previously associated with analgesic response (rs2952768), isoniazid metabolism [NAT2*5 (rs1801280), NAT2*6 (rs1799930), NAT2*12 (rs1208)], interferon response (rs12979860), and salbutamol response (rs6988229).

### 2.9 Loss‐of‐Function Variants and Human Knockouts

To characterize gene‐disrupting variation in CAAH populations, we cataloged putative loss‐of‐function (pLoF) variants and human knockouts. We identified 3436 pLoF variants: 76.9% frameshift, 12.7% stop‐gained, 4.9% splice‐donor, and 3.9% splice‐acceptor, with the remainder stop‐lost (Figure 7E). We also detected 3381 high‐confidence protein‐truncating variants (PTVs), predominantly heterozygous (96.2%), of which 78.8% were unique to this dataset (Figure 7E). Gene set enrichment analyses highlighted pathways involved in system development, cell motility, and signaling molecules and interactions (Figure 7F). Uyghur showed the strongest enrichment signals, including pathways related to cytoskeletal function in muscle cells, head development, sensory system development, and 22q11.2 copy‐number variation syndrome (Figure S39D). We further identified 129 homozygous PTVs across 118 genes, eight of which were absent from dbSNP v156. The newly observed homozygous PTVs included *CCDC88C* in all CAAH populations; *NOL9* and two *CRK* alleles specific to Uzbek; *ZMIZ2* unique to Tajik; *RELN* restricted to Hazara; and *BPIFB3* and *TGM2* alleles detected only in Kyrgyz. Notably, a GWAS of 19,629 Europeans linked *RELN* variants to regional brain volumes and cognitive and mental‐health traits [95].

## 3 Discussion

### 3.1 Fine‐Scale Population Structure of CAAH Populations

Central Asia has long been a nexus of trans‐Eurasian genetic, cultural, and economic exchange. Nevertheless, the demographic history of contemporary Central Asians and the genetic underpinnings of population‐specific traits and diseases remain incompletely resolved. Our study provides a new WGS resource for 20 CAAH populations and uses it, together with extensive modern and ancient references, to reconstruct fine‐scale structure and deep demographic history. The WGS data revealed a pronounced east–west genetic gradient among CAAH populations that was broadly consistent with previous SNP‐array [10] and ancient DNA work [3], but our analyses refined this picture in several ways. Tajik, Karluk, Turkmen, and Uzbek populations showed elevated ancestry related to Iranian and Anatolian farmers and EEHGs, and occupied the West Eurasian side of the CAAH continuum. In contrast, Kyrgyz, Uyghur, Karakalpak, and Hazara showed stronger affinities with Altaic groups in East Eurasia and with ancient populations of the Mongolian Plateau, with reduced West Eurasian ancestry. Dungan stood out as an East Eurasian outlier among CAAH populations, with predominant ANEA ancestry and close ties to Sino‐Tibetan speakers and ancient Yellow River populations. These patterns confirm that present‐day Central Asian populations are not a single homogeneous admixed group but instead comprise multiple lineages with distinct combinations of West Eurasian farmer, steppe, and East Asian ancestries. An array‐based study reporting greater allele sharing between Kyrgyz, Uzbek, and Turkmen populations with diverse East Asian/Siberian groups than with West Eurasians, in contrast with these findings [10], underscoring the value of high‐density genotyping and broad sampling for reconstructing population history. By combining qpAdm and haplotype‐based methods, we further identified stratified ancestries within CAAH: elevated West Eurasian ancestry in Tajik, Karluk, Turkmen, and Uzbek; complex east–west admixture in Karakalpak, Uyghur, and Hazara; enriched East Eurasian ancestry in Kyrgyz; and exceptionally high ANEA‐related ancestry in Dungan.

When we placed these genetic patterns in their linguistic and geographic context, we observed both alignments and mismatches that were informative about past movements. Among Turkic‐speaking populations sampled across Eurasia, we identified four broad clusters corresponding to the eastern, Siberian, Central Asian, and western regions. Genetic distances, ancestry components, and haplotype sharing varied gradually across space, and PCs correlated strongly with latitude and longitude, suggesting that isolation by distance and geographically structured admixture were major drivers of Turkic diversity. When viewed alongside archaeological and linguistic work that has proposed southern Siberia/Mongolia (“steppe hypothesis”) [10], Northeast China (“farming/Northeast Asia” hypothesis linked to ANEA‐related ancestry and the Xinglongwa/Hongshan cultures) [96], and the Altai–Sayan [97] regions as potential Turkic homelands, our findings were most consistent with an origin in Northeast Asia followed by westward dispersal and recurrent gene flow with local West Eurasian groups. We explicitly acknowledge, however, that these findings refine rather than resolve the debate on Turkic origins, and that multiple scenarios remain compatible with the genetic data.

Indo‐European groups in our dataset formed two main clines, one spanning Eastern Europe and another linking Central Asia and the Caucasus. Tajik groups showed elevated Iranian and Anatolian farmer and EEHG‐related ancestries, underscoring a deep West Eurasian imprint. In contrast, Hazara showed a strong affinity with Altaic groups in northern East Asia and southern Siberia, and Dungan aligned more closely with East Asians despite residing in Central Asia. These contrasts illustrate that language, geography, and genetics are only partially aligned and that long‐distance migrations, such as the historical movements of Chinese Hui communities into Central Asia and the complex ethnogenesis of Hazara, have played a major role in shaping present‐day structure [98, 99].

The timing of these contributions differed across populations, with most admixture pulses clustering around 23–34 generations ago, in line with the Song–Yuan period of intensified trans‐Eurasian contact and the rise of the Mongol Empire, and with additional older and more recent pulses in specific groups such as Tajiks. Given the Bronze Age onset of east–west admixture [9], these estimates are probably conservative lower bounds. Coalescent‐based divergence estimates further showed that the Tajik‐related lineage descended from West Eurasian ancestors, whereas Dungan and Altaic‐speaking Central Asians shared an origin with East Eurasian lineages, followed by asymmetric gene flow between these clades. These models move beyond qualitative descriptions and provide quantitative constraints on when and how West and East Eurasian ancestries combined in Central Asia.

### 3.2 Signatures of Local Biological Adaptation

The influence of selection on human health and disease remains a central question in human genetics. We performed complementary genome‐wide scans to characterize local adaptation across the four CAAH subgroups. AFd_e_‐based analyses were designed to detect post‐admixture deviations from ancestry‐based expectations and, when integrated with ancient DNA time series, enabled us to trace subgroup‐specific allele frequency shifts across major ecological and subsistence transitions. These analyses revealed widespread yet heterogeneous post‐admixture signals, with recurrent enrichment in metabolic, immune, and neural pathways. Convergent signals at *EBF2* in CAAH‐West and CAAH‐Mixed support post‐admixture selection on adipogenesis‐related pathways, potentially linked to energy balance and thermoregulation. Additional subgroup‐enriched signals at *MTAP* in CAAH‐Mixed, *CLDN10* in CAAH‐Kyrgyz, and *LINGO2* in CAAH‐Dungan further suggest divergence in metabolic and physiological adaptation. The temporal trajectories of several derived alleles, particularly across the transitions from hunting‐gathering to farming and from farming to pastoralism, further imply that shifts in subsistence strategy likely contributed to these adaptive patterns. However, the trajectories were not uniform, arguing against a single region‐wide selective regime.

Immune‐related loci also showed strong post‐admixture differentiation. In CAAH‐West, *TRAF1* and *PHF19* carried alleles associated with RA, allergic disease, white blood cell count, and lymphocyte count. Given the substantial West Eurasian ancestry in this subgroup, some of these alleles may have been introduced through admixture and then reshaped by local selective pressures, consistent with previous reports that immune‐risk alleles enriched in western Eurasian hunter‐gatherers later declined under changing selective regimes [100]. In parallel, *LPXN*, *PPP4R1L*, and *GIMAP6* in CAAH‐Dungan point to additional subgroup‐specific immune remodeling. We also detected prominent signals at loci related to nervous system function. Shared signals at *FILIP1* across multiple subgroups suggest partially convergent pressures on neurodevelopment‐related pathways, whereas *CHRNA3* and *HDAC9* in CAAH‐Mixed, *SPON1* in CAAH‐Kyrgyz, and *TCF4* in CAAH‐Dungan indicate more localized selective histories. The intersection between AFd_e_ variants and east–west HDVs further highlighted post‐admixture selection at *THOP1* and *VPS41*, emphasizing both localized adaptation and the intricate mosaic of east–west ancestry. Because many of these loci are pleiotropic, the associated traits should be viewed as functional clues rather than direct targets of selection.

The CMS scan provided an independent and broader assessment of local adaptation by integrating the cross‐population number of segregating sites by length (XP‐nSL), the integrated haplotype score (iHS), the population branch statistic (PBS), and the derived allele frequency differentiation (ΔDAF). Whereas AFd_e_ specifically captured post‐admixture adaptation, CMS identified stronger composite genome‐wide signals, confirming several AFd_e_ candidates while also revealing additional loci not highlighted by the window‐based scan. The overlap between the two approaches supports robust adaptive signals at regions including *PSD3*, *SNX7*, *XIRP2*, *CTNNA3*, *HSD17B12*, *NAALADL2*, *SPON1*, *ZNF385B*, *ADAMTS17*, *HPSE2*, *PTPRD*, and *TCF4*. CMS‐specific signals at *MPP5*, *COL21A1*, and *SMAD5* in CAAH‐West and CAAH‐Mixed; *PRIM2* in CAAH‐Kyrgyz and CAAH‐Dungan; *ECD*, *KLHL24*, and *CCDC25* in CAAH‐West; *HLA‐DPA1*/*HLA‐DPB1* in CAAH‐Mixed; *IPMK* in CAAH‐Kyrgyz, and *ULK4* and *ANKRD31* in CAAH‐Dungan further extend the adaptive landscape. Together, these results support a model in which adaptation in CAAH populations emerged through the interplay of admixture, ecological transition, and cultural change, generating a fine‐scale mosaic of metabolic, immune, and neurodevelopmental responses.

### 3.3 Archaic Introgression Landscape

We comprehensively characterized the genomic distribution and potential functional relevance of Neanderthal and Denisovan introgression in CAAH populations. The distribution of Neanderthal‐derived segments varied across subgroups, consistent with heterogeneous demographic histories and local admixture. We identified 104 Neanderthal‐inherited segments absent from the 1KGP, underscoring the complexity of archaic introgression and its contribution to regional genomic diversity. Several CAAH‐specific AISs, including one overlapping *BCL11A* segment, fell within loci previously linked to hematopoietic and hematological phenotypes [101], suggesting that some regionally enriched introgressed segments fall within functionally important genomic regions. More broadly, many of the annotated signals recapitulated prior observations from other populations, particularly at immunity‐ and metabolism‐related loci, whereas others expanded the catalog of AISs observed in Central Asia. Subgroup‐specific Neanderthal AISs overlapped loci previously associated with immune, metabolic, neurodevelopmental, and pharmacogenomic traits, including *L3MBTL3* and *RAD51B* in CAAH‐West, *MAD1L1* in CAAH‐Mixed, *SLCO1B1* in CAAH‐Kyrgyz, and *UBE2L3* in CAAH‐Dungan. These patterns suggest that Neanderthal‐derived sequences may contribute to phenotypic heterogeneity across CAAH populations. By contrast, Denisovan‐derived segments were fewer and showed substantially more limited functional annotation than Neanderthal‐derived sequences. Pathway analyses revealed shared enrichment for muscle cell differentiation and nervous system/muscle development across Neanderthal‐ and Denisovan‐derived sequences, suggesting that archaic alleles may converge on broader physiological pathways. Despite the widespread presence of AISs, we found only limited evidence for adaptive introgression. We identified several archaic haplotypes with signatures of putative positive selection, including a Neanderthal‐derived variant at *CLOCK* in CAAH‐West. Temporal shifts in derived allele frequencies at immune‐ and metabolism‐related loci suggest changing evolutionary pressures, potentially linked to shifts in subsistence strategy or pathogen exposure. Nevertheless, many subgroup‐specific AISs, particularly in CAAH‐Kyrgyz and CAAH‐Dungan, lacked strong functional annotation or clear evidence of adaptive significance, suggesting either modest biological effects, limited power in current datasets, or both. Overall, these findings highlight the value of region‐focused genomic studies for refining the demographic and functional landscape of archaic introgression in historically understudied populations. They also underscore the need for validation in larger datasets and direct phenotype‐linked analyses.

### 3.4 Central Asian Genomes Inform Human Disease and Health

Our analysis expands the catalog of medically relevant and pharmacogenomically annotated variations in historically understudied Central Asian populations. By integrating ClinVar, ACMG‐SF, pharmacogenomic annotations, and pLoF/PTV calls, we provide a population‐level view of the distribution of previously reported variants across CAAH populations. Importantly, these resources are used here primarily to describe the landscape of medically relevant variations, rather than to support direct clinical reporting or interpretation without orthogonal validation. Several observations illustrate the importance of ancestry‐aware interpretation. For example, the relatively high frequency of *SCN5A*‐rs1805124 in some CAAH subgroups, together with its broader frequency distribution in African populations, suggests that variant interpretation may vary substantially across ancestry backgrounds and that classifications derived largely from non‐Central Asian datasets should be applied with caution. Similarly, marked differences in the frequency spectra of pharmacogenomic loci, including *VKORC1*, *CYP2C9*, and *CYP4F2* (warfarin metabolism), *ATIC* (methotrexate response), *ADRB2* (salmeterol response), and NAT2*5/*6/*12 (isoniazid response), indicate that Central Asian populations may be underrepresented in existing pharmacogenomic reference frameworks. These results provide a foundation for preemptive screening and for optimizing drug regimens across regional populations. However, we do not interpret them as direct evidence for immediate clinical implementation without dedicated validation in regional cohorts. Our analyses extended the catalog of human knockouts by identifying high‐confidence LoF alleles and newly observed homozygous PTVs. These findings are valuable for future genotype–phenotype mapping and for prioritizing genes for functional follow‐up. However, rather than representing definitive clinical assertions, these variants should be regarded as candidates for future validation and deeper functional study.

Recent WGS studies in Oceania, Southeast Asia, South Asia, Africa, and the Americas have shown the power of integrative population genomic frameworks that combine dense modern sequencing, ancient genomic data, and complementary analytical approaches to reconstruct demographic history and characterize biologically relevant variation [25]. These studies highlight that region‐specific genomic resources are essential for resolving fine‐scale population structure, identifying local adaptation signals, and improving the representation of diverse ancestries in medical genomics. In this context, the CAGDP extends this framework to Central Asia, a historically pivotal yet genomically underrepresented region of Eurasia. Beyond its immediate findings, the CAGDP provides a foundation for future genomic diversity research in this region. Broader sampling across ethnolinguistic groups and geographic regions will be necessary to build a more representative reference resource and improve imputation performance in understudied populations. The fine‐scale substructure identified here has important implications for study design, informing both modern cohort construction and ancient DNA sampling while reducing bias in downstream analyses. Integrating the CAGDP with global genomic resources will support more inclusive precision medicine and pharmacogenomics research across Eurasia. Continued advances in high‐depth ancient DNA sequencing and long‐read sequencing will further refine complex demographic history, structural variation, and haplotype architecture, thereby deepening our understanding of genomic diversity, evolutionary history, and health‐related variation in Central Asian populations.

## 4 Conclusion

The pilot phase of the CAGDP provides a high‐resolution whole‐genome framework for investigating the demographic history, biological adaptation, archaic introgression, and medically relevant variation of contemporary Central Asian and Afghan Hazara populations. By analyzing 166 individuals from 20 populations and integrating these genomes with extensive modern and ancient Eurasian reference data, we show that present‐day Central Asians do not constitute a single admixed population but instead form a structured genetic mosaic shaped by varying contributions from West Eurasian farmer‐ and steppe‐related ancestries and multiple East Eurasian lineages. Our analyses refine the genetic landscape of Eurasian Turkic‐ and Indo‐European‐speaking groups, identify clear signatures of long‐distance migration in the Dungan and Hazara, and date most major east‒west admixture events to the last millennium, consistent with intensified trans‐Eurasian contact during the historical period. Beyond population history, we identify subgroup‐specific post‐admixture adaptive signals enriched in metabolic, immune, and neurodevelopmental pathways, suggesting that ecological transition, subsistence change, and local selective pressures jointly shaped biological diversity across Central Asia. We also expand the regional catalog of Neanderthal‐ and Denisovan‐derived sequences, identify introgressed loci potentially relevant to immunity, metabolism, neurological traits, and drug response, and show that archaic ancestry in Central Asia includes both shared Eurasian components and locally distinctive features. In parallel, our characterization of pathogenic, pharmacogenomic, and pLoF variants reveals substantial ancestry‐related differences in medically relevant allele frequencies and underscores the need for more representative genomic resources in this historically understudied region. Together, these findings establish Central Asia as a critical region for resolving Eurasian population history and for advancing the inclusiveness of human evolutionary genomics and precision medicine. They also provide a foundation for future work involving broader sampling, deeper sequencing, integration of ancient DNA, and phenotype‐linked validation to further clarify how migration, admixture, adaptation, and archaic inheritance have shaped genomic diversity and health‐related variation across Central Asia.

## 5 Methods

### 5.1 Ethical Approval, Sample Collection, and Genome Sequencing

This work was developed through collaborations among anthropological, genetic, and linguistic scientists within the CAGDP Consortium, aiming to improve the representation of the genomic diversity of ethnolinguistically diverse populations and to provide comprehensive insights into their population history, biological adaptation, archaic introgression, and medical implications. The study was approved by the Medical Ethics Committee of the National Centre for Biotechnology of Kazakhstan (protocol No. 2 of 10 June 2020), the Nazarbayev University Institutional Research Ethics Committee (protocol No. 17 of 16 April 2019), the Biomedical Ethics Review Committee of West China Hospital, Sichuan University (2024–1463), and all procedures were conducted in accordance with the Declaration of Helsinki [102]. The participants were selected based on the criterion that all four grandparents were indigenous to the respective ethnic groups. Informed consent was obtained from all participants. Saliva samples were collected from 166 individuals from five Central Asian countries (Kyrgyzstan, Kazakhstan, Uzbekistan, Tajikistan, and Turkmenistan) and Afghanistan in South Asia via the Oragene DNA Self‐Collection Kit (OG‐500, DNA Genotek, Canada). The sampled individuals included Kyrgyz people from various regions in Kyrgyzstan (Talas, Issyk‐Kul, Naryn, Osh, Batken, and Jalal‐Abad), Uzbeks from the Turkistan Region in Kazakhstan, Karakalpaks from the Karakalpakstan Region in Uzbekistan, Karluks from the Khatlon Region in Tajikistan, Uyghurs from the Almaty Region in Kazakhstan, Turkmens from Daşoguz and Mary Regions in Turkmenistan, Dungans from the Jambyl Region in Kazakhstan, Tajiks from several regions in Tajikistan (Sughd, Gorno‐Badakhshan, and Khatlon), and Hazaras from various provinces in Afghanistan (Bamyan, Ghazni, Oruzgan, and Ghor). Genomic DNA was extracted using the prepIT‐L2P kit (DNA Genotek, Canada) according to the manufacturer's protocol, and the DNA concentration was measured using a Qubit 3.0 fluorometer (Thermo Fisher Scientific). Library preparation was performed according to the standard Illumina protocol, with all libraries purified via Agencourt AMPure XP (Beckman Coulter). The final libraries were quantified via qPCR. WGS was performed on the Illumina HiSeq 4000 platform with a preset sequencing depth of 10× across all samples.

### 5.2 Sequencing Quality Control and Variant Discovery

Samples were retained if at least 80% of all bases had a quality score of 30 or higher. Sentieon Genomics tools (version 202010.04) were used to map reads from the newly generated genomic data, following the Best Practices guidelines of Genome Analysis Toolkit [103]. The raw sequencing reads were aligned to the human reference genome assemblies GRCh37 and GRCh38 using BWA v0.7.17 [104]. PCR duplicates were removed with Sentieon Dedup. The aligned sequencing reads from different lanes were then sorted and merged via SAMtools v1.10 [105]. GVCF files were generated with the HaplotypeCaller module, and joint variant calling was performed via the CombineGVCFs and GenotypeGVCFs modules [106]. We assessed relatedness among newly sequenced individuals with KING v.2.3.0 [107] and PLINK v.1.90 [108], excluding one individual from each pair with a kinship coefficient > 0.0442 or PI_HAT > 0.0884. We used VariantEval to assess the GRCh37‐based callset, including raw and filtered variant counts, the proportion of variants absent from dbSNP v156, and Ti/Tv ratios. We classified identified SNPs into four groups according to MAF: (i) singleton variants, (ii) rare variants (MAF < 0.01), (iii) intermediate variants (0.01 ≤ MAF < 0.05), and (iv) common variants (MAF ≥ 0.05). We further performed variant‐level quality control with bcftools, including sequencing depth distribution, call rate, the correlation between singleton burden and sequencing depth, and Ti/Tv ratios across allele frequency bins.

### 5.3 Dataset Merging

The newly generated GRCh37‐based dataset was merged with the published Human Origins (HO) and 1240K datasets (V54.1), which include contemporary and ancient populations worldwide in the Allen Ancient DNA Resource [50]. We filtered the merged data using PLINK v1.90 [108], excluding variants with missing call rates greater than 0.05 (–geno 0.05), MAFs less than 0.05 (–maf 0.05), and exact test *p*‐values for Hardy‒Weinberg equilibrium less than 10^−6^ (–hwe 10^−6^). Samples with missing call rates exceeding 0.1 (–mind 0.1) were also removed. Relatedness coefficients between individuals within populations were estimated via KING v.2.3.0 [107] and PLINK v.1.90 [108]. The final merged HO dataset comprised 5476 individuals and 593,050 SNPs, whereas the merged 1240K dataset included 2923 individuals and 1,135,264 SNPs (Table S1). To investigate the fine‐scale genetic substructure of Turkic‐ and Altaic‐speaking populations across Eurasia, the merged HO dataset was further integrated with previously published data [4, 10]. The resulting dataset comprised 5798 individuals and 81,480 SNPs. The extremely low‐density extended HO dataset was used to investigate the population structure of the newly sequenced CAAH populations and their linguistically related groups. The merged low‐density HO dataset and the medium‐density 1240K dataset were primarily applied for admixture profile modeling and demographic history reconstruction. We merged the GRCh37‐based callset with the 1KGP VCF dataset using VCFtools, mainly for archaic introgression analyses. In addition, we merged the GRCh38‐based callset with 126 Northwest Chinese individuals generated in the pilot phase of the 10K_CPGDP [20] and 709 high‐depth whole‐genome sequences from 38 Eurasian populations in the HGDP [18], all processed with the same read‐mapping and variant‐calling pipeline, to generate a high‐density WGS dataset. We retained only biallelic autosomal variants, which were used mainly to infer signatures of natural selection (Figure S40).

### 5.4 Population Structure Inference

To investigate the genetic affinity and population stratification of CAAH populations within Eurasian and regional contexts, we conducted PCAs using the smartpca program in EIGENSOFT v7.2.1 [109], with numoutlieriter set to 0 and lsqproject set to YES. Mlabri and ancient populations were projected onto the first two principal components. Scatter plots were generated using R v4.2.2 and custom scripts. An unsupervised model‐based clustering analysis was performed using the maximum‐likelihood method implemented in ADMIXTURE (v1.3.0) across Eurasian and regional populations [110]. The number of ancestral components (K) ranged from 2 to 20, with a 10‐fold cross‐validation setting (–cv = 10). SNPs in strong linkage disequilibrium (LD) were pruned using the “–indep‐pairwise 200 25 0.4” command in PLINK v1.90. For each value of K, 100 bootstrap resamplings were conducted with different random seeds. *F*_ST_ distances between population pairs were calculated using PLINK v1.90, excluding populations with sample sizes less than five. The phylogenetic relationships among ethnolinguistically diverse target populations were further estimated using TreeMix v1.13 [111]. The frequency spectrum for each population was calculated using PLINK v1.90 and subsequently utilized as input for constructing TreeMix‐based topologies. Migration edges, ranging from 0 to 7, were explored using the ‐m parameter to assess potential gene flow events.

### 5.5 Relationship Between Genetics and Geography Among Turkic Populations

Geographic coordinates of each sampling site were used to perform Procrustes analyses in the R package vegan (https://github.com/vegandevs/vegan) [112], following the approach of Atkinson et al. [113]. The first two PCs from the PCA plot of Turkic groups were rotated and scaled to minimize squared differences between PCs and geographic coordinates after projection onto a sphere. The Procrustes M^2^ statistic, representing the sum of squared differences between the two ordinations, was calculated. Statistical significance was assessed using *p*‐values derived from 100,000 permutation tests.

### 5.6 F‐Statistical Analysis

Various forms of the *f_3_*‐ and *f_4_*‐statistics were computed using ADMIXTOOLS v.7.0.2 to measure shared genetic drift between two populations since their divergence from a common ancestral population [114]. Outgroup *f_3_*‐statistics of the form *f_3_* (Target, Reference; Mbuti) were performed to estimate overall allele sharing between all target and reference populations. Additionally, admixture *f_3_*‐statistics of the form *f_3_* (Ref1, Ref2; Target) were calculated for all pairs of modern or ancient references to test whether the target population was derived from the admixture of populations related to two predefined ancestry surrogates. Tree‐like relatedness between four populations was further evaluated using the qpDstat package in ADMIXTOOLS with the *f_4_* model (*f_4_* Mode: YES). The block jackknife method was used to assess the statistical significance of the *f_4_*‐values. To quantify genetic homogeneity and heterogeneity between two target populations, we conducted symmetrical *f_4_* (Target1, Target2; Ref, Mbuti).

### 5.7 Admixture Modeling With qpWave and qpAdm

To determine whether target population pairs (left populations) formed a single clade relative to a set of outgroups (right populations), we conducted rank tests using the qpWave program (version 1520) [114]. Admixture modeling based on the *f_4_*‐statistics was performed using the qpAdm program (version 1520) within the ADMIXTOOLS package. This approach assessed the number of distinct ancestry streams from surrogate populations needed to explain the genetic makeup of the target populations and estimated the corresponding admixture proportions. The parameters “allsnps: YES” and “details: YES” were applied to include all SNPs across the four populations and display the specific entries of the *f_4_*‐matrix and Z‐scores. Only models with *p* ≥ 0.05 were considered as well‐fitting.

### 5.8 Admixture Time Estimation

Long‐distance migrations and subsequent admixtures between populations can lead to an exponential decay of admixture‐induced LD. To compute the weighted LD decays and infer the admixture dates, we utilized MALDER v1.0 with additional parameters set to mindis: 0.005 and jackknife: YES [115]. Multiple modern populations from East and West Eurasia were employed as potential ancestral sources, testing all possible combinations.

### 5.9 Haplogroup Classification and Clustering Analysis

Y‐chromosomal haplogroups were classified using Y‐LineageTracker 1.3.0 [116] and the hGrpr2.py package within HaploGrouper [117]. For the HaploGrouper‐based analysis, two reference files, treeFileNEW_isogg2019.txt and snpFile_b38_isogg2019.txt, were utilized. Maternal haplogroups were classified using HaploGrep2 [118] and HaploGrouper, based on PhyloTree17.

### 5.10 Inference of Sex‐Biased Admixture

To evaluate potential sex‐biased admixture processes in CAAH populations, ADMIXTURE was used to estimate ancestry proportions from surrogate source populations on both autosomes and the X chromosome. Z‐scores were calculated to assess differences between autosome‐ and X chromosome‐based admixture proportions for a given ancestry, using the formula $Z=\left(P_{A}-P_{X}\right)/\sqrt{{\sigma_{A}}^{2}+{\sigma_{X}}^{2}}$, where P_A_ and P_X_ represent the ancestry proportions on autosomes and X chromosomes, respectively, and σ_A_ and σ_X_ are their corresponding standard errors [119]. A positive Z‐score indicates a higher ancestry proportion on autosomes than on the X chromosome, suggesting male‐driven admixture; conversely, a negative Z‐score indicates female‐driven admixture. Due to the original use of this formula to assess differences in qpAdm‐based ancestry proportions and the unavailability of X‐chromosome genotype data for the source populations modeled with qpAdm in this study, ADMIXTURE was employed to estimate admixture proportions from the WGS dataset. Additionally, a Student's *t*‐test was performed [99], yielding a *p*‐value < 0.05, indicating sex‐biased admixture in the target population.

### 5.11 Haplotype‐Based Fine‐Scale Population Structure Reconstruction

#### 5.11.1 Segmented Haplotype Estimation and Fine‐scale Population Structure Identification

Stricter filtering criteria of geno: 0.1 and mind: 0.1 were applied to prune genome‐wide SNPs. The Segmented HAPlotype Estimation & Imputation Tool (SHAPEIT v2.r904) was then used to estimate haplotypes based on the genetic maps from HapMap phase II b37 [120, 121]. To identify an optimal starting point for the estimated haplotypes and generate more parsimonious graphs, we performed haplotype phasing with ten burn‐in iterations (–burn 10), ten pruning iterations (–prune 10), and 30 main iterations (–main 30) of MCMC (Markov chain Monte Carlo). The fine‐scale population structure was subsequently analyzed using fineSTRUCTURE v4.0 based on the phased haplotypes [122].

#### 5.11.2 Shared IBD Fragment Estimation

Refined IBD (refined‐ibd.17Jan20.102.jar) was used to detect shared IBD segments between each pair of individuals [121]. A minimum length of 0.1 cM was set for the identified IBD fragments using the parameter length = 0.1. The detected IBD segments were classified into three categories: 1–5 cM, 5–10 cM, and > 10 cM. Segments in the 1–5 cM range likely reflect ancient genetic connections dating back 1500–2500 years; those in the 5–10 cM range correspond to a time interval of approximately 500–1500 years ago; and segments greater than 10 cM likely indicate recent shared ancestry within the last 500 years. To provide an overview of IBD sharing, we summed the counts and lengths of IBD segments for each pair of individuals and calculated the population mean for each block.

#### 5.11.3 Admixture Events Inferred From fastGLOBETROTTER

ChromoPainterv2 was used to paint each chromosome of the recipient populations as a series of haplotype chunks and to estimate the number of ancestry chunks inherited from donor populations [122]. Initially, ChromoPainterv2 was run to paint each phased haploid of the first ten recipient individuals, using ten iterations of expectation‐maximization with the “‐in” switch and “‐iM” emission parameters to estimate the chunk size and switch error rate. A Perl script was subsequently applied to estimate the switch (‐n) and emission (‐M) rates across individuals. The estimated parameters were then used to rerun ChromoPainter v2 to construct the copy vectors, modeling both recipient and donor individuals as patchworks of donor haplotypes. The “chunk length” output files obtained across all chromosomes were summed. FastGLOBETROTTER was then employed to identify, date, and describe admixture events in target populations, using the chunk‐length output and painting samples with the options “prop.ind: 1” and “null.ind: 1” [123]. The significance of the inferred admixture dates was estimated using “bootstrap.date.ind: 1” and “bootstrap.num: 100” options. SourcefindV2 was also used to infer admixture models based on haplotype sharing identified by ChromoPainter.

### 5.12 Demographic History Inference

For each newly sequenced population, SMC++ was used to infer the population size history [124]. Input files were prepared based on the genotypes of all called biallelic SNPs and included all individuals from each population. The two samples with the highest sequencing depth in each population were specified as the distinguished lineages. SMC++ was run with a mutation rate of 1.25 × 10^−8^ per base pair per generation and a constant generation time of 29 years. To infer the population divergence time, MSMC2 (multiple sequentially Markovian coalescent) was employed [125]. The two samples with the highest sequencing depth were selected from each target population for MSMC2 analysis. The French and Han Chinese populations from the HGDP were chosen as representatives of European and East Asian ancestries, respectively. MSMC2 analyses were performed in accordance with standard recommendations. Each analysis was run independently three times to estimate coalescence rates within population1 and population2, as well as between the two populations. Absolute time estimates were calculated using the same mutation rate and generation time as those adopted in SMC++. The relative cross‐coalescence rate (rCCR) was calculated, and the divergence time for each population pair was inferred at the point where the rCCR reached 0.5.

### 5.13 Complex Demographic Model Reconstruction

To investigate the complex demographic history of Central Asian populations, demographic models were reconstructed via fastsimcoal v2.7 [126]. The French and Han populations from the HGDP were selected as proxies for European and East Asian ancestral lineages, respectively. From each of the four CAAH subgroups, one representative population, Tajik, Karakalpak, Kyrgyz, and Dungan, was included to capture the regional genetic diversity of four representative genetic lineages. SNPs were filtered according to the following criteria: (i) missing in at least one sample; (ii) deviation from Hardy–Weinberg equilibrium (*p* < 10^−^^4^) in at least one population; (iii) located within a CpG island; (iv) located in a coding region; and (v) absence of ancestral state information. Pairwise site frequency spectra (SFS) were generated for these populations, and we evaluated multiple demographic models incorporating plausible divergence and gene flow scenarios: (i) Tajik shares a common ancestor with French, whereas Karakalpak, Kyrgyz, and Dungan share a common ancestor with Han, with Karakalpak and Kyrgyz forming a lineage that diverged earlier than Dungan and Han with or without instantaneous West Eurasian gene flow events (modelA/B); (ii) Tajik, Karakalpak, and Kyrgyz share a common ancestor with French, with the common ancestor of Karakalpak and Kyrgyz diverging first with or without East Eurasian gene flow event (modelC/D); and (iii) Karakalpak and Kyrgyz originate from an East Eurasian lineage with the common ancestor of Han and Dungan, but later received instantaneous gene flow from either French or Tajik (modelE/F/G). Model parameters were estimated by maximizing the composite likelihood of the observed SFS under each model, with multiple independent runs performed to ensure convergence and robustness. Parameter estimates were scaled using a mutation rate of 1.25 × 10^−^^8^ per site per generation and a generation time of 29 years. The best‐fitting model was identified based on likelihood values, providing quantitative estimates of the timing and magnitude of divergence and admixture events that have shaped the genetic landscape of Central Asia.

### 5.14 Medical Relevance and Biological Adaptation

#### 5.14.1 Variant Annotation

Variant annotation of the GRCh37‐based dataset was performed using Ensembl Variant Effect Predictor, with a focus on variants annotated as pathogenic or likely pathogenic [127]. These pathogenic variants were reviewed against the ACMG‐SF v3.2 gene panel in accordance with the ACMG criteria [128]. SnpEff v4.3, with default parameters, was employed for LoF predictions [129], defining a variant as a PTV if it was annotated as “frameshift”, “transcript ablation”, “splice‐acceptor”, “splice‐donor”, or “stop‐gained”. To generate a list of high‐confidence PTVs, we restricted variants to high‐confidence regions as determined by Genome in a Bottle (ftp://ftp‐trace.ncbi.nlm.nih.gov/ReferenceSamples/giab/data/AshkenazimTrio/analysis/NIST_v4.1_SmallVariantDraftBenchmark_12182019/GRCh37/HG002_GRCh37_1_22_v4.1_draft_benchmark.bed).

#### 5.14.2 Inference of Candidate Loci of Local Adaptation

To identify potential signals of natural selection while minimizing confounding from heterogeneous ancestry profiles, we divided CAAH into four genetically distinct subgroups based on haplotype clustering. CAAH‐West (*n* = 69) included individuals from Tajik, Karluk, Turkmen, and Uzbek groups and showed a higher proportion of West Eurasian ancestry. CAAH‐Mixed (*n* = 57) comprised Uyghur, Hazara, and Karakalpak and reflected balanced West and East Eurasian contributions. CAAH‐Kyrgyz (*n* = 20) consisted of Kyrgyz individuals and showed relatively greater East Eurasian ancestry, whereas CAAH‐Dungan (*n* = 20) primarily included Dungan individuals and displayed strong affinity with East Asian populations. We modeled each CAAH subgroup as a two‐way admixture of East and West Eurasian ancestries, using Han Chinese and French from the HGDP as references. For genome‐wide variants, we calculated AFd_e_ within each subgroup as the absolute difference between AF_obs_ and AF_exp_, where AF_exp_ = *f*_East_×ɑ + *f*_West_×(1‐ɑ) [51, 52]. Here, *f*_East_ and *f*_West_ denote allele frequencies of a SNP in the East and West Eurasian ancestral populations, and ɑ represents the East Eurasian ancestry proportion estimated by ADMIXTURE. Using a window‐based approach [130], we ranked genes by AFd_e_ quantiles and retained the top 0.1% as candidates for local adaptation. To detect post‐admixture selection signals, we identified HDVs between East and West Eurasians (*F*_ST_ > 0.2) and retained those with AFd_e_ > 0.1 in CAAH subgroups [51, 52]. We annotated candidate variants using the GWAS Catalog (v.2022‐09‐14) and assessed pathway enrichment with Metascape [131]. We visualized allele‐frequency trajectories using the AGES browser (https://reich‐ages.rc.hms.harvard.edu/#/) and validated temporal selection signals using X‐statistics derived from ancient DNA time series [61].

We then performed a composite selection scan by integrating two haplotype‐based measures (XP‐nSL and iHS), PBS, and ΔDAF into a CMS score. These complementary statistics capture extended haplotype structure and population‐specific shifts in allele frequencies. We computed XP‐nSL and iHS using Selscan v2.0.0 and normalized them with Norm, excluding variants with EHH decay < 0.05. We inferred ancestral alleles from reference data and calculated DAF using PLINK. We obtained ΔDAF by comparing DAF between the target and reference populations. We calculated PBS for each variant under a three‐population framework, using Han Chinese from Beijing and Northern Europeans from Utah in the 1KGP as the reference and outgroup populations, respectively. We first estimated pairwise *F*_ST_ among the three populations and then derived PBS to quantify locus‐specific divergence in each focal subgroup. Finally, we calculated CMS for each site and selected variants within the top 0.1% of the genome‐wide distribution as candidate loci for hard selective sweeps:

$$\mathrm{CMS}=-\log\prod_{i}Pi$$

where *Pi* represents the empirical *p*‐value from each test.

### 5.15 Archaic Introgression Estimation

The IBDmix (v.1.0.1) method was initially employed to detect AISs likely derived from Neanderthals [81]. Owing to IBDmix's limited sensitivity for detecting Denisovan‐derived AISs, Sprime v.07Dec18.5e2 was used exclusively to identify Denisovan‐derived AISs in CAAH [82]. Additionally, Sprime was utilized to detect Neanderthal‐derived AISs. The newly generated data were merged with 1KGP WGS data for IBDmix analysis. All multi‐allelic variants and InDels from the archaic genome and the merged dataset were removed, with a focus on autosomal variants. Archaic ancestry detection was then conducted within each CAAH subgroup to mitigate the impact of population structure. To minimize misclassification due to incomplete lineage sorting, only AISs with a logarithm of the odds ratio for the linkage score above 4 and a length greater than 50 kb were included in downstream analyses. To maximize the detection of Neanderthal‐like sequences, a conservative approach was used for filtering the callset. After the initial identification of Neanderthal‐ and Denisovan‐derived sequences via IBDmix, the Neanderthal‐related callset was refined by masking regions identified as Denisovan‐like segments in Africans that were also classified as Neanderthal‐derived sequences in any population.

For the Sprime analysis, we designated the four previously defined CAAH subgroups as target populations and used YRI (Yoruba in Ibadan, Nigeria, *n* = 108) as the outgroup. We applied a score threshold of 150,000, along with default parameters, to optimize performance and accuracy. To infer the origin of AISs and quantify the proportion of putative archaic alleles matching reference genomes, we calculated the match rate for each AIS as the ratio of matched sites to the total number of compared sites. We used the kde2d function in the R package to generate contour plots and visualize distinct waves of archaic introgression. We further modeled the distribution of match rates across non‐overlapping archaic ancestry segments following Browning et al. [82]. For Neanderthal ancestry, we fitted both a single truncated normal model and a two‐component truncated normal mixture model to the match rate distribution and estimated parameters by maximum likelihood. We compared model fit using log‐likelihood, Akaike Information Criterion, Bayesian Information Criterion, and a likelihood ratio test (LRT) with 3 degrees of freedom. For Denisovan ancestry, we applied an analogous framework, comparing a single normal model with a two‐component normal mixture model. We performed an LRT with 3 degrees of freedom and applied the Bonferroni correction to 4 tests, one per population, resulting in a significance threshold of 0.0125.

We extracted segments with clear Neanderthal or Denisovan origins for further exploration of the biological functions of the identified AISs. We retained only segments containing at least 30 putatively introgressed alleles, comparable to the Vindija Neanderthal genome or to the Denisovan genome. We classified segments as Neanderthal‐derived when the match rate to the Vindija Neanderthal genome exceeded 0.6 and to the Altai Denisovan genome was below 0.3. We classified segments as Denisovan‐derived when the match rate exceeded 0.3 to the Altai Denisovan genome and was below 0.3 to the Vindija Neanderthal genome. We again retained only AISs with a log‐odds linkage score greater than 4 and a length exceeding 50 kb to reduce misclassification due to incomplete lineage sorting [33]. Finally, we intersected the Neanderthal‐inherited AISs identified by both IBDmix and Sprime using bedtools to generate a high‐confidence set for adaptive introgression analyses [132]. To identify high‐confidence signals of adaptive introgression, we focused on archaic variants supported by both allele‐frequency‐based and haplotype‐based (iHS) selection signals.

## Author Contributions

M.G.W., S.H.D., and G.L.H. contributed equally to this work. Conceptualization: M.G.W., G.L.H., L.H.W., M.Z., C.L. Software: S.H.D., Q.X.S., L.T.L., J.Z. Validation: G.L.H., M.G.W., S.H.D., Q.X.S., R.K.T., J.Z., Z.S. Formal analysis: M.G.W., G.L.H., S.H.D, Q.X.S, L.T.L., J.Z. Investigation: M.G.W., G.L.H., Z.S., C.L. Resources: GLH, MGW, LHW, MZ. Data curation: G.L.H., M.G.W., M.Z., C.L. Writing – original draft: M.G.W., G.L.H. Writing – review & editing: M.G.W., G.L.H., R.K.T., Z.S., C.L., L.H.W., M.Z. Visualization: M.G.W., G.L.H., S.H.D., Q.X.S., L.T.L. Supervision: M.G.W., G.L.H., C.L. Project administration: G.L.H., M.G.W., L.H.W., M.Z., C.L. Funding acquisition: G.L.H., M.G.W., L.H.W., M.Z.

## Funding

This work was supported by the National Natural Science Foundation of China (82572153 and 82402203), Science Committee of the Ministry of Education and Science of the Republic of Kazakhstan (AP23486749 and BR18574101), Faculty Development Competitive Research Grants Programs of Nazarbayev University (SST2019012), Major Project of the National Social Science Foundation of China (23&ZD203), Open Project of the Key Laboratory of Forensic Genetics of the Ministry of Public Security (2024FGKFKT02), Center for Archaeological Science of Sichuan University (23SASA01 and 24SASB03), and 1·3·5 Project for Disciplines of Excellence, West China Hospital, Sichuan University (ZYJC20002).

[Correction added on 10 July 2026, after first online publication: the first grant number of Science Committee of the Ministry of Education and Science of the Republic of Kazakhstan was changed from AP2348674 to AP23486749.]

## Conflicts of Interest

The authors declare no conflicts of interest.

## Supporting information




**Supporting File 1**: advs76320‐sup‐0001‐SuppMat.pdf.

**Supporting File 2**: advs76320‐sup‐0002‐TablesS1‐S10.xlsx.

## Data Availability

The raw data of 166 individuals were submitted to the Genome Variation Map database under accession number GVM000900 (https://bigd.big.ac.cn/gvm/getProjectDetail?Project=GVM000900). All the data used here are included in the supplementary materials. Reference populations can be found in publicly available datasets, such as the Human Genetic Diversity Project dataset, Oceania genomic resource, and the Allen Ancient DNA Resource (HO and 1240K datasets) from the David Reich Lab (https://dataverse.harvard.edu/dataset.xhtml?persistentId=doi:10.7910/DVN/FFIDCW&version=8.0). The raw data can be provided by Guanglin He and Mengge Wang, pending scientific review and a completed material transfer agreement with Sichuan University. Requests for the raw data should be submitted to: Guanglin He at guanglinhescu@163.com, Mengge Wang at menggewang2021@163.com, and Maxat Zhabagin at mzhabagin@gmail.com.
