# Supplementary material for: Whole‐Genome Sequencing Pilot of the Central Asian Genomic Diversity Project Reveals Distinct Histories, Adaptation, and Introgression
Source: Adv Sci (Weinh). 2026 Jul 7;13(53):e76320. doi: 10.1002/advs.76320 (PMC13339586; doi:10.1002/advs.76320)
Supplement: Supplementary file 1 [file ADVS-13-e76320-s001.pdf]

Supporting Information

Whole-genome sequencing pilot of the Central Asian Genomic Diversity Project reveals distinct histories, adaptation, and introgression

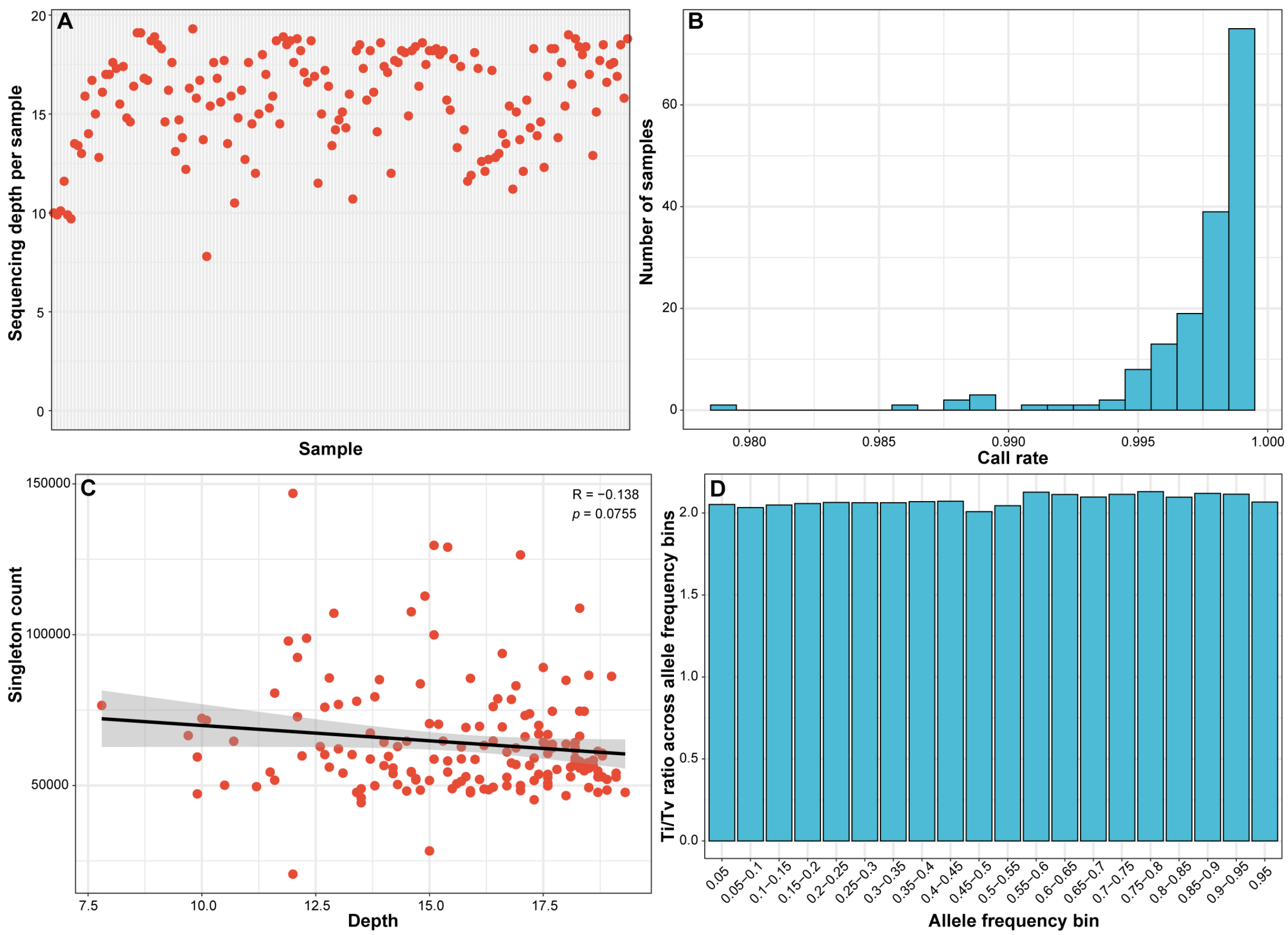

Figure S1. Sequencing quality metrics for newly sequenced Central Asians and Afghan Hazaras (CAAH). (**A**) Distribution of sequencing depth per sample. (**B**) Distribution of call rates across CAAH individuals. (**C**) Distribution of singleton counts across sequencing depths. (**D**) Ti/Tv ratios across allele frequency bins.

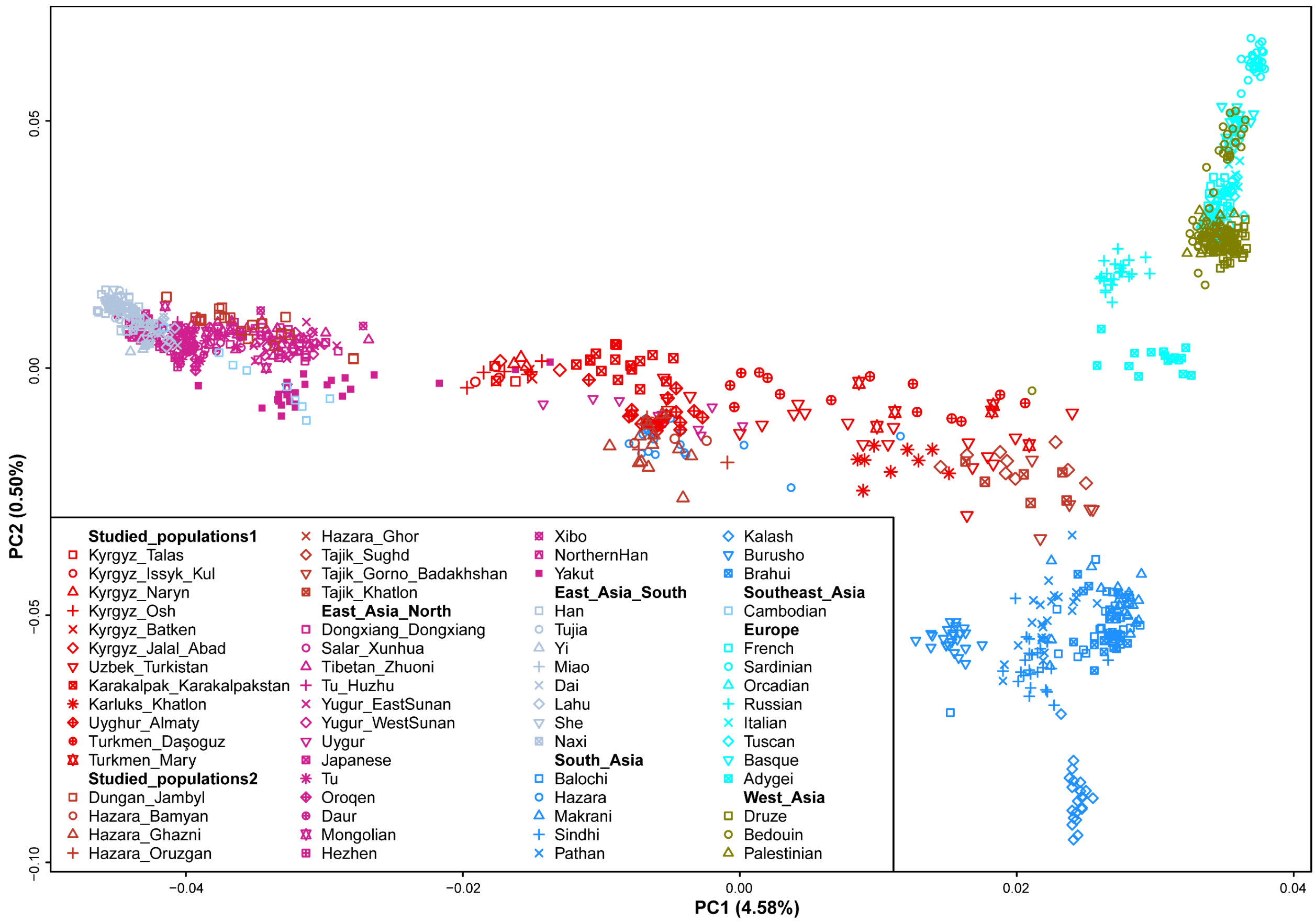

Figure S2. Genetic relationships between newly sequenced CAAH and Eurasian reference populations revealed based on the GRCh38-based whole-genome sequencing dataset. Eurasian reference populations were grouped by geographic region.

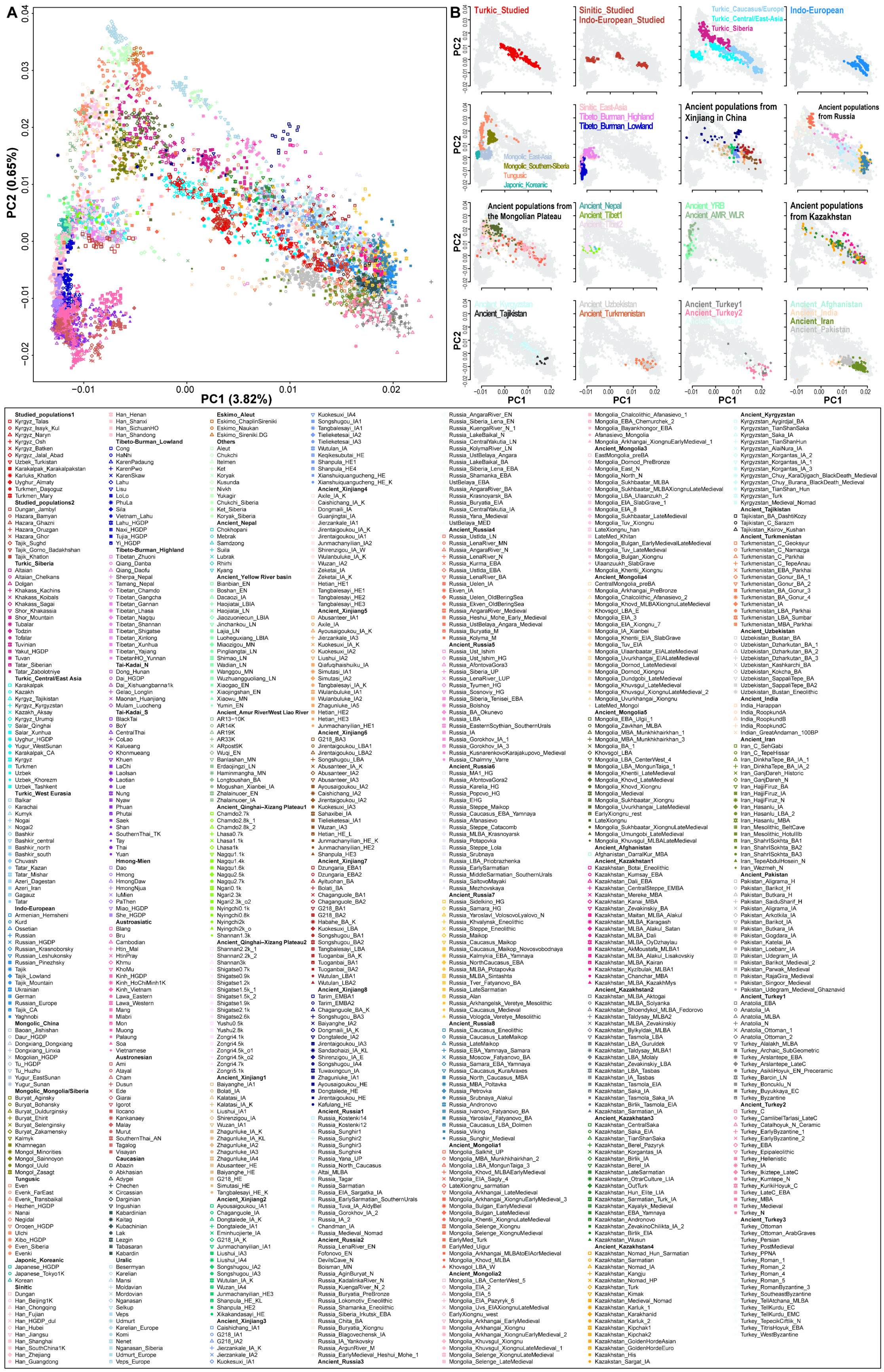

A
PC2 (0.65%)
PC1 (3.82%)
B
Turkic_Studied
Sinitic_Studied
Indo-European_Studied
Turkic_Caucasus/Europe
Turkic_Central/East-Asia
Turkic_Siberia
Indo-European
Mongolic_East-Asia
Mongolic_Southern-Siberia
Tungusic
Japonic_Koreanic
Sinitic_East-Asia
Tibeto_Burman_Highland
Tibeto_Burman_Lowland
Ancient populations from Xinjiang in China
Ancient populations from Russia
Ancient populations from the Mongolian Plateau
Ancient_Nepal
Ancient_Tibet
Ancient_YRB
Ancient_AMR_WLR
Ancient populations from Kazakhstan
Ancient_Kyrgyzstan
Ancient_Tajikistan
Ancient_Uzbekistan
Ancient_Turkmenistan
Ancient_Turkey1
Ancient_Turkey2
Ancient_Afghanistan
Ancient_India
Ancient_Iran
Ancient_Pakistan
PC2
PC1

Figure S3. Genetic relationships between newly sequenced CAAH individuals and spatiotemporally diverse Eurasian reference populations, based on the merged Human Origins (HO) dataset. (**A**) The population structure of 920 modern and ancient Eurasian populations included in the merged HO dataset, with ancient populations projected onto the first two principal components (PCs). Modern populations are color-coded by linguistic affiliation, while ancient populations are color-coded by geographic region. (**B**) Clustering patterns of the same 920 populations, highlighting linguistically distinct modern groups and geographically distinct ancient groups. For clarity of visualization, densely represented ancient groups from the same region were randomly grouped.

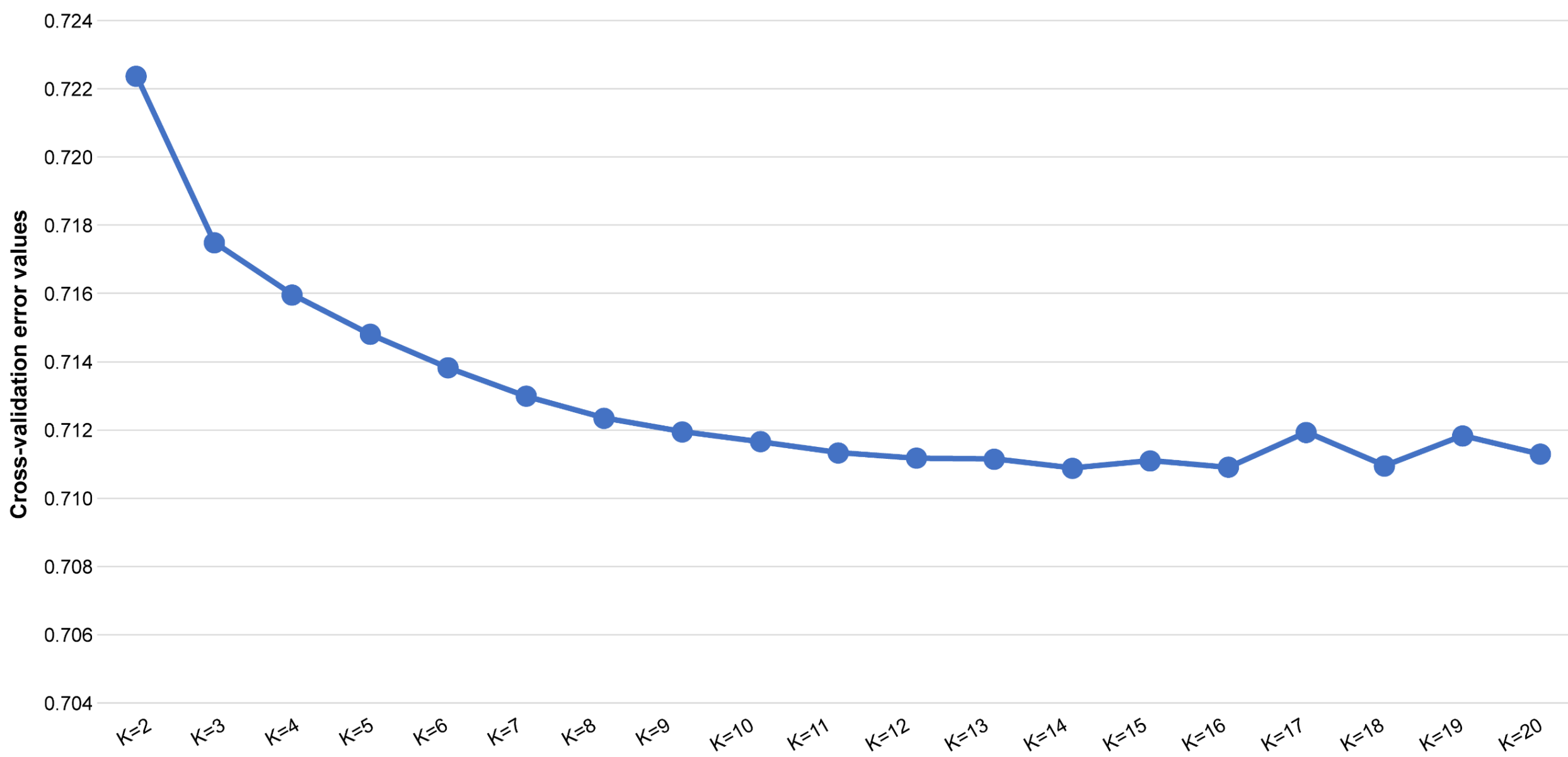

Figure S4. Cross-validation error values under the ADMIXTURE model (K = 13).

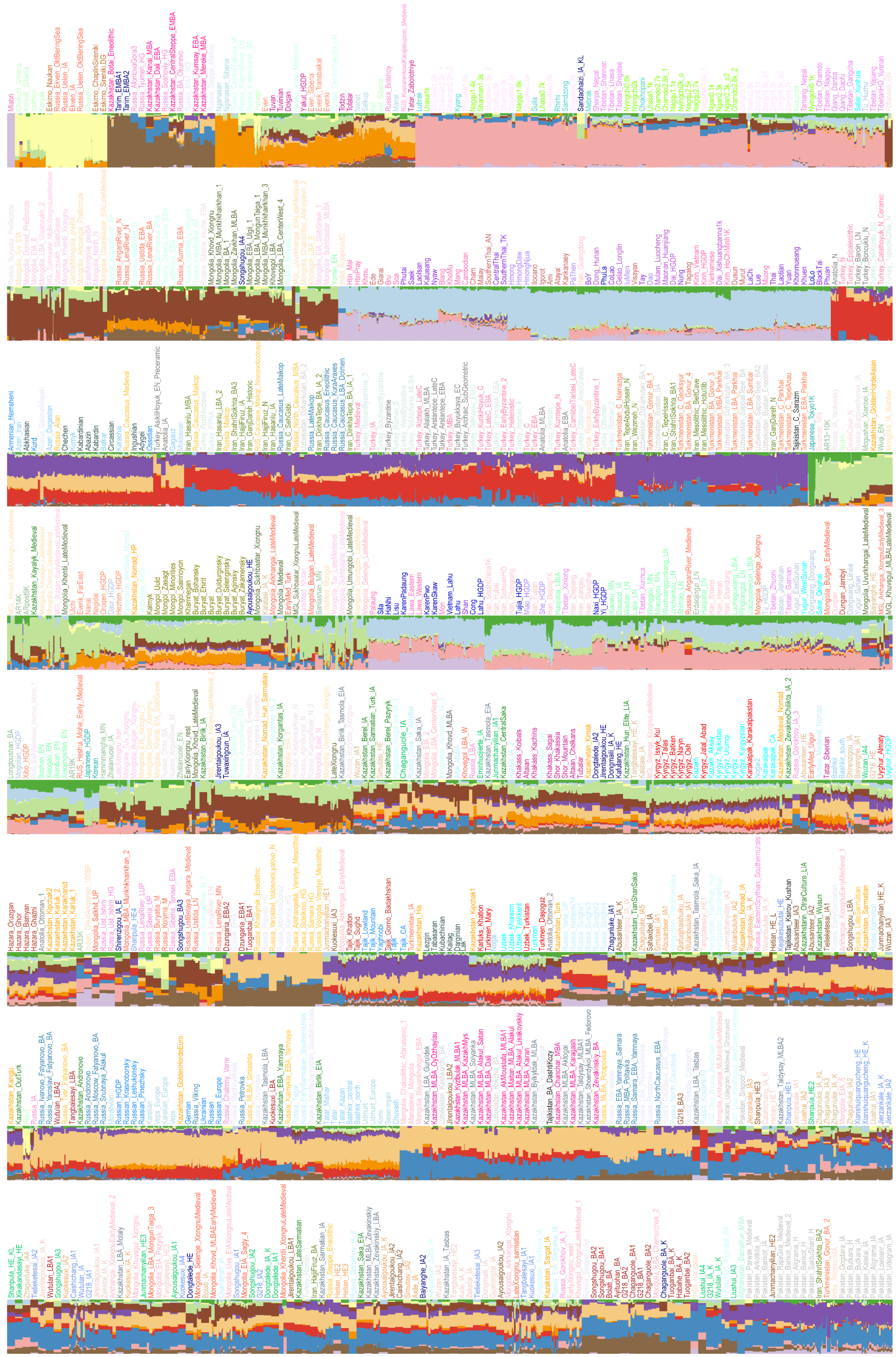

Figure S5. Model-based ADMIXTURE results of 920 spatiotemporally diverse modern and

ancient Eurasian populations included in the merged HO dataset at K = 13. Population grouping is shown in Figure S3.

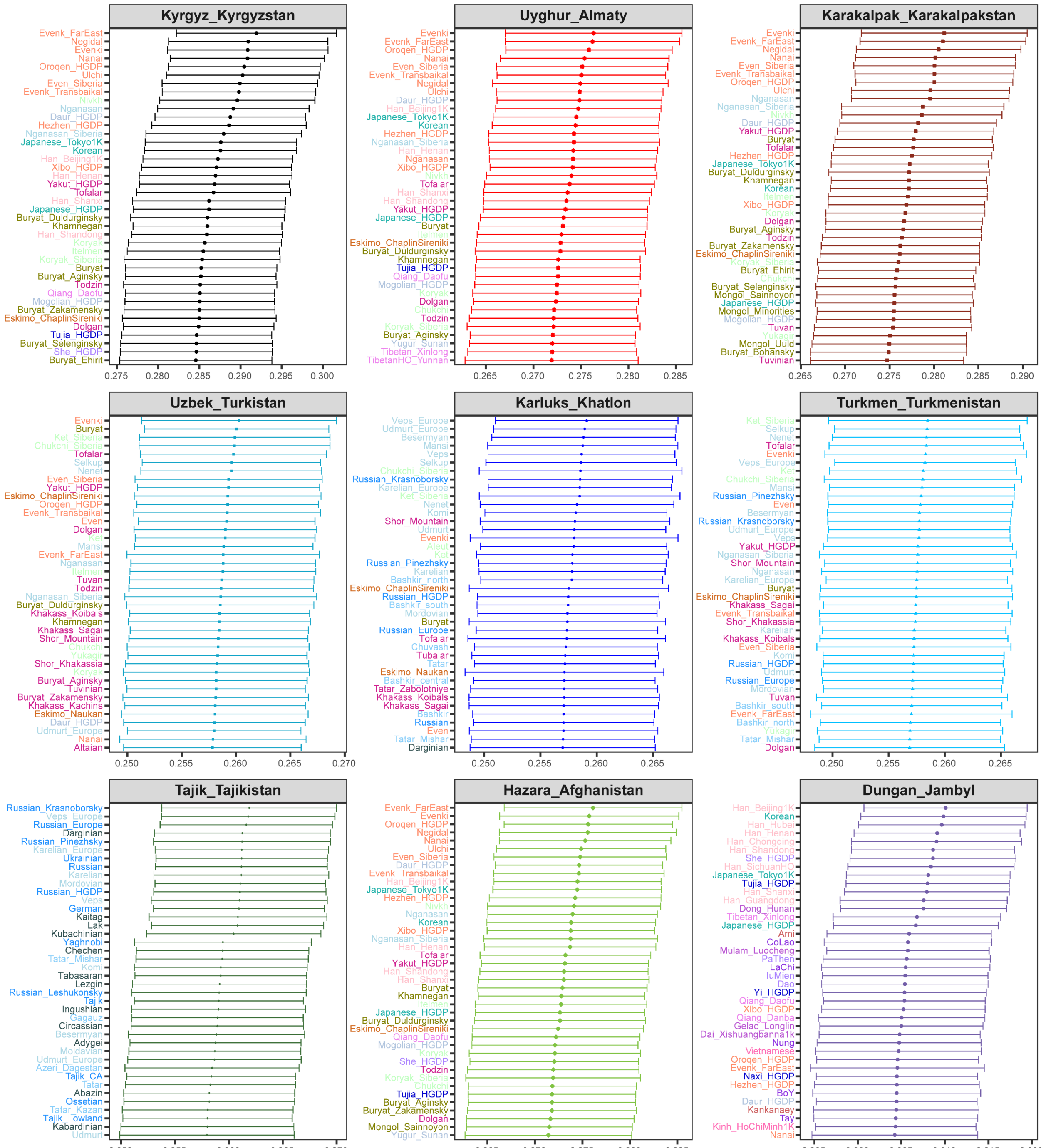

Figure S6. The shared drift between CAAH and modern Eurasian reference populations estimated using outgroup f_3-statistics of the form f_3(CAAH, Modern Eurasians; Mbuti). The top 40 f_3-values of each population are presented. Populations were grouped according to language family, consistent with the classifications used in the PCA plot presented in Figure S3.

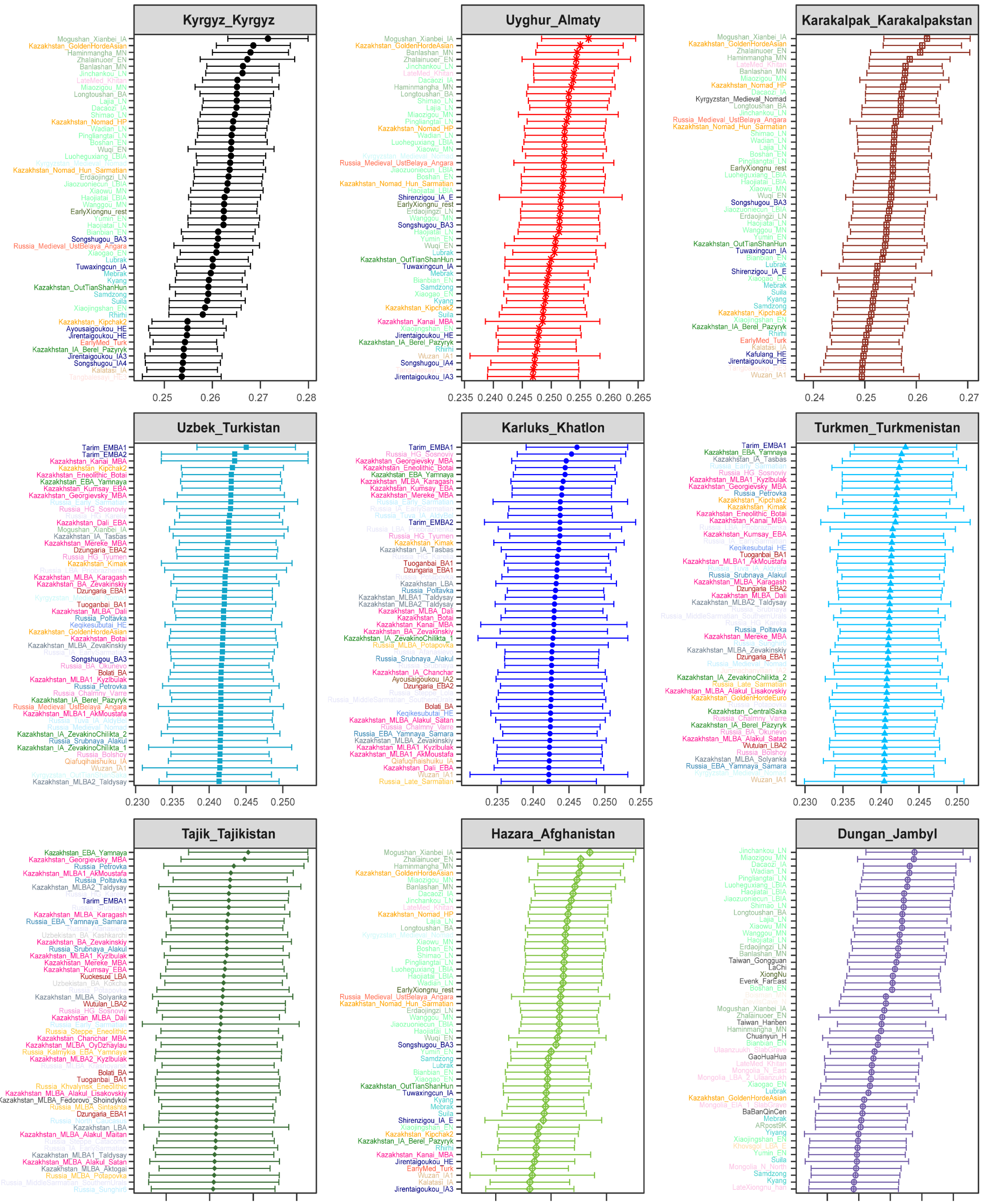

Figure S7. The shared drift between CAAH and ancient Eurasian reference populations estimated using outgroup f_3-statistics of the form f_3(CAAH, Ancient Eurasians; Mbuti). The top 40 f_3-values of each population are presented. The grouping is consistent with the PCA plots shown in Figure S3.

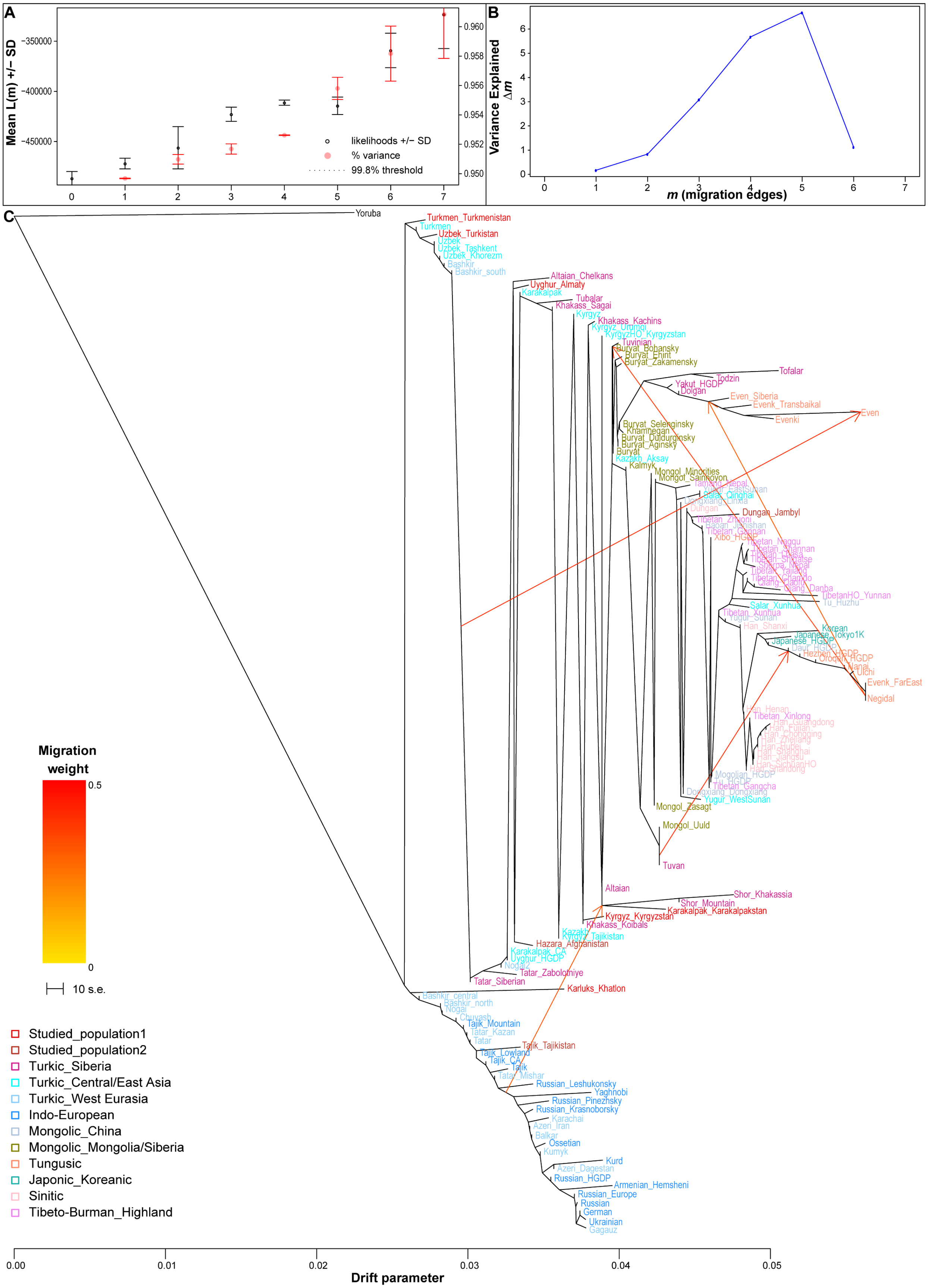
A
Mean L(m) +/− SD
−350000
−400000
−450000
0
1
2
3
4
5
6
7
0.960
0.958
0.956
0.954
0.952
0.950
likelihoods +/− SD
% variance
99.8% threshold
B
Variance Explained
Δm
m (migration edges)
C
Yoruba
Migration weight
0.5
0
10 s.e.
Studied_population1
Studied_population2
Turkic_Siberia
Turkic_Central/East Asia
Turkic_West Eurasia
Indo-European
Mongolic_China
Mongolic_Mongolia/Siberia
Tungusic
Japonic_Koreanic
Sinitic
Tibeto-Burman_Highland
0.00
0.01
0.02
0.03
0.04
0.05
Drift parameter

Figure S8. Treemix-based topology of CAAH and linguistically related but geographically distinct populations. (**A**) Mean and standard deviation of composite likelihood (left axis, black circles) and proportion of variance explained (right axis, red circles). (**B**) Second-order rate of change in likelihood. (**C**) The Maximum likelihood tree of Altaic-, Indo-European-, and Sino-Tibetan-speaking populations inferred from five migration events, rooted with Yoruba. Populations are colored by language families as in Figure S3, with migration arrows shaded according to weight.

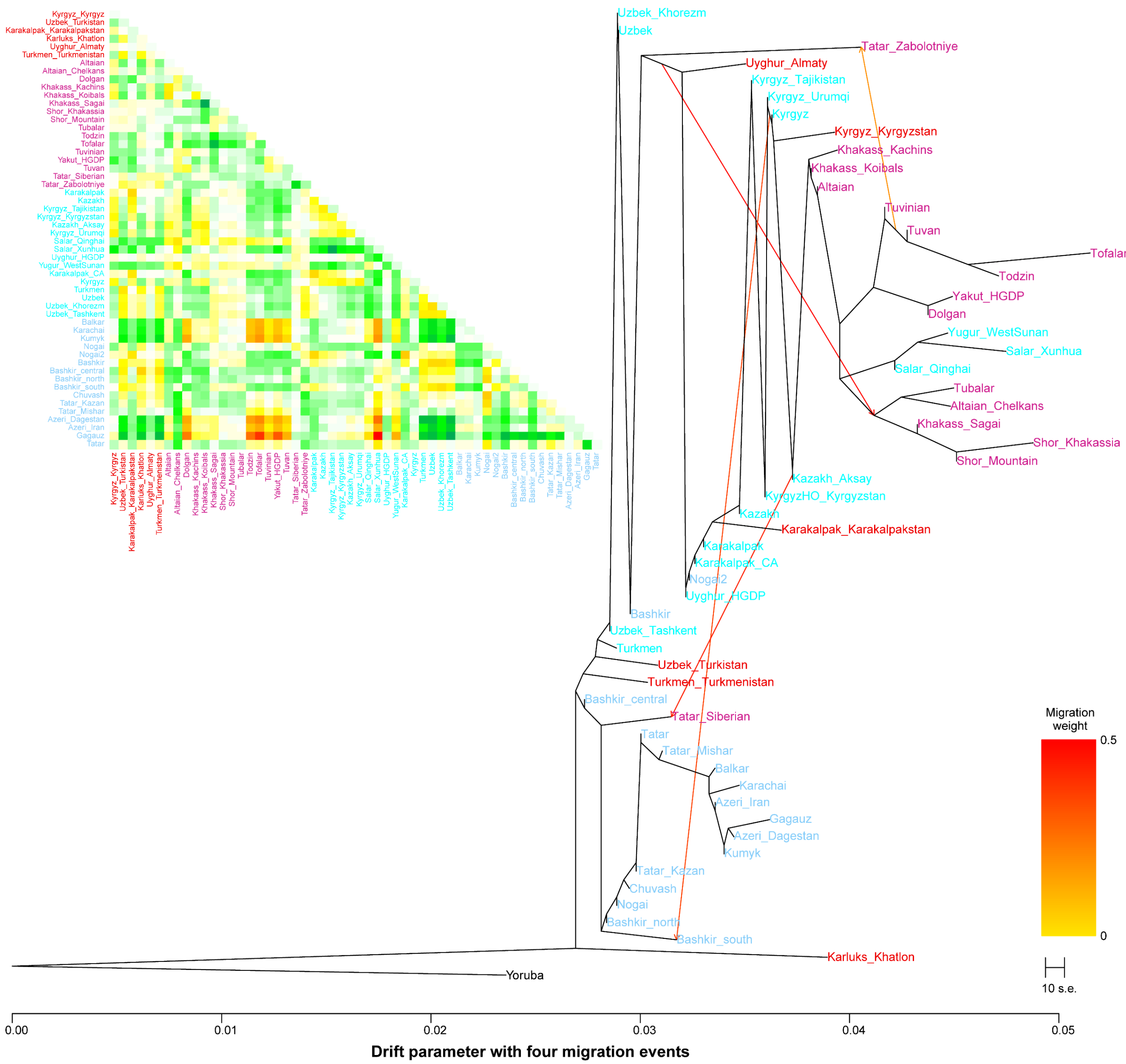

Figure S9. Treemix-based topology of 54 geographically distinct Turkic-speaking populations. The left panel displays the residuals from the maximum likelihood tree. The right panel depicts the inferred maximum likelihood tree incorporating four migration events. Population colors correspond to the groupings presented in Figure S3.

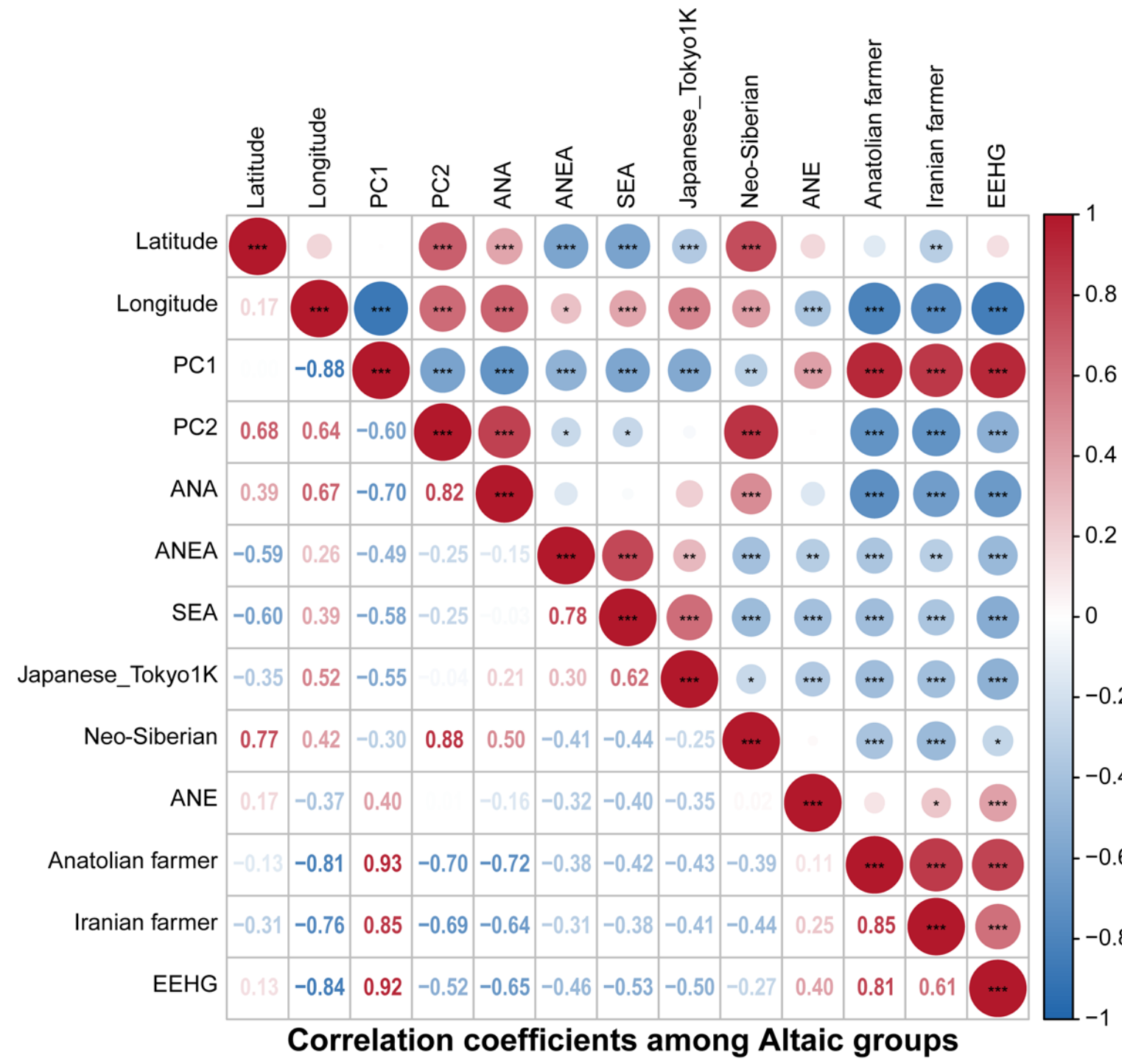

Figure S10. Correlation between geographic coordinates (latitude and longitude) and genetic features (PC1, PC2, and estimated ancestral components) among 94 Altaic-speaking populations, including Turkic, Mongolic, Tungusic, Koreanic, and Japonic groups. See table S1 for detailed population information. The font color in the lower left panel, as well as the color, size of the circles, and the asterisks in the upper right panel, follow the same conventions as in Figure 2B.

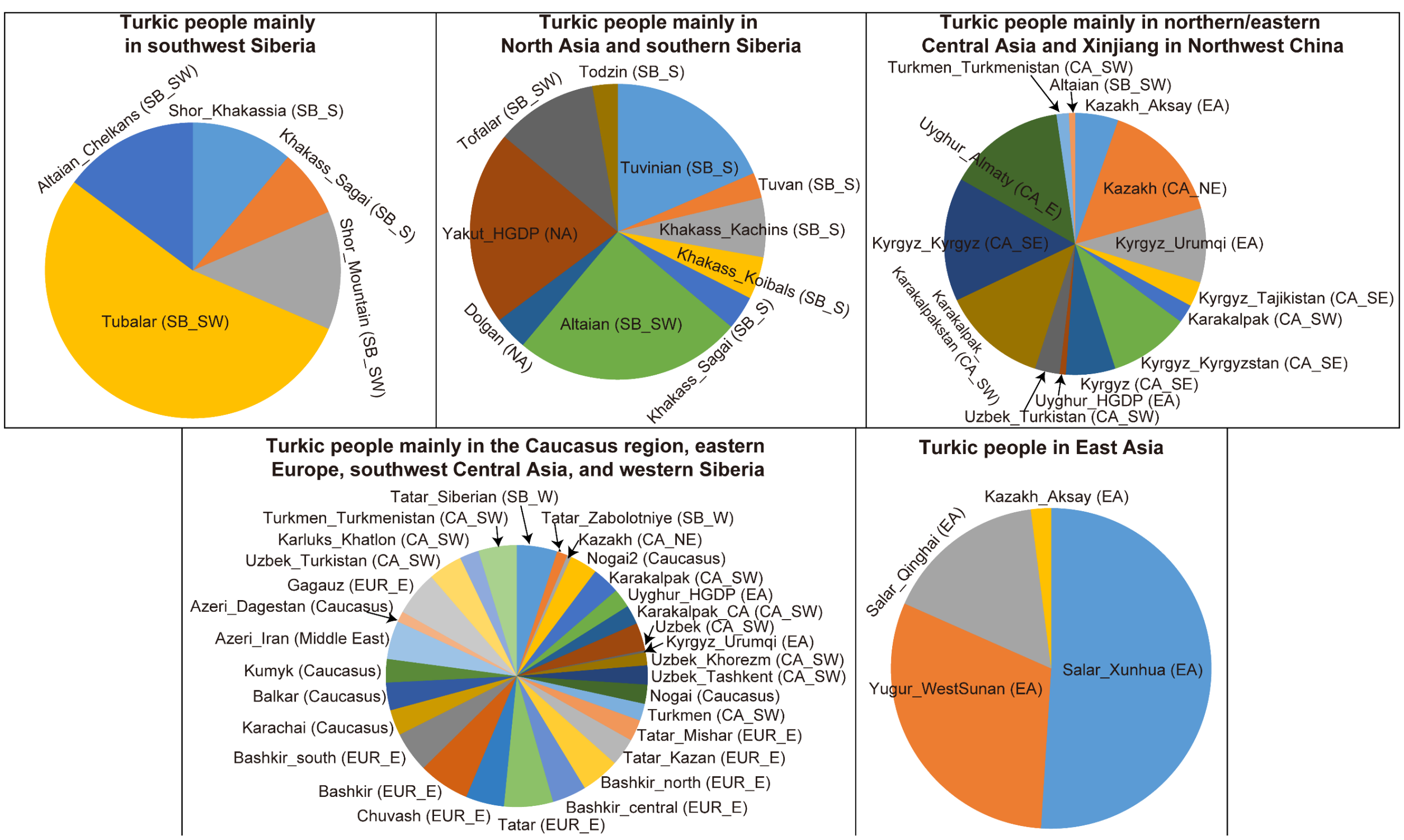

Figure S11. Population composition of Turkic-related clusters inferred by fineSTRUCTURE (Figure 2E). SB_S, Southern Siberia; SB_SW, Southwestern Siberia; SB_W, Western Siberia; NA, North Asia; CA_SW, Southwestern Central Asia; CA_NE, Northeastern Central Asia; CA_SE, Southeastern Central Asia; EA, East Asia; EUR_E, Eastern Europe.

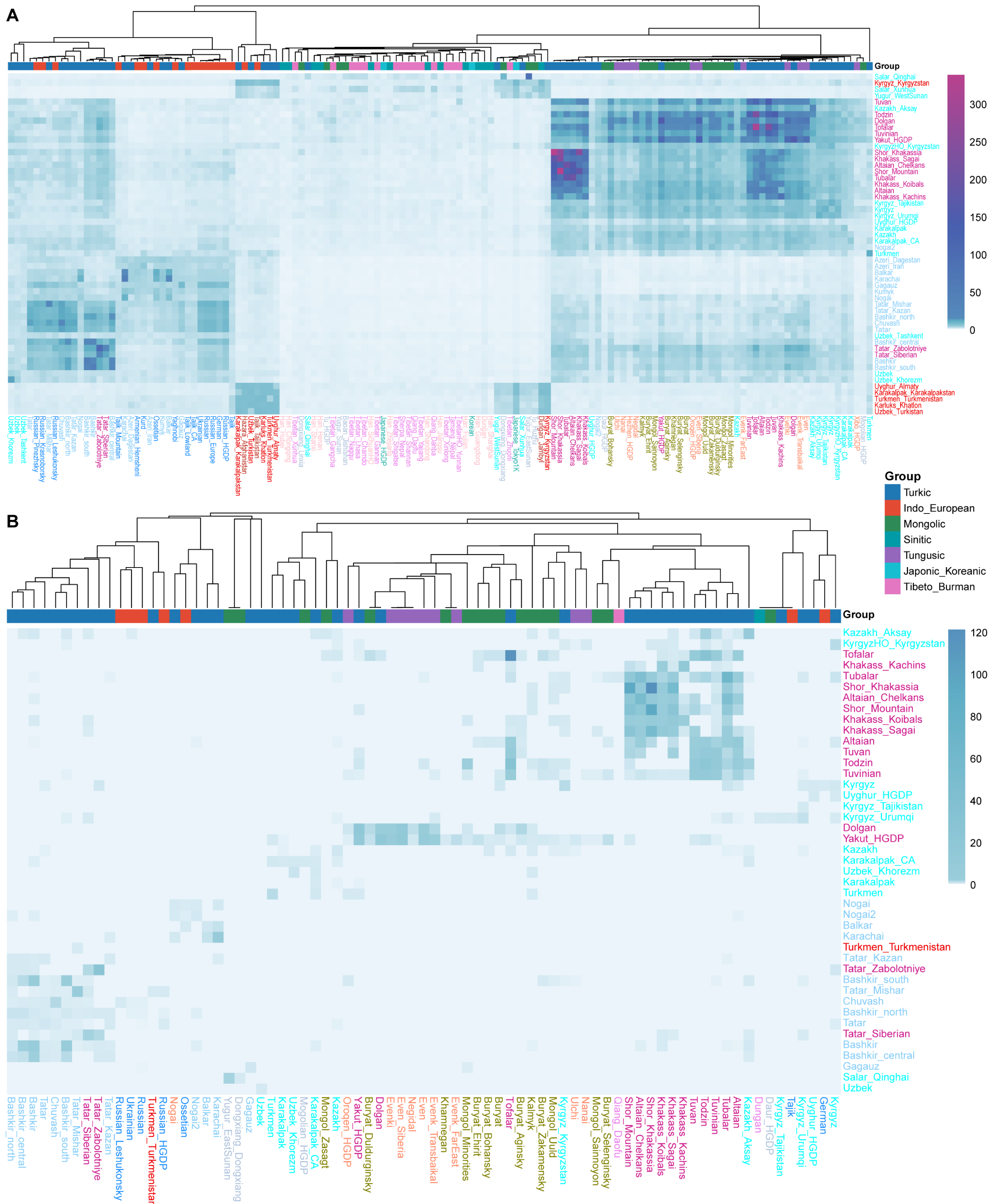

Figure S12. Identity-by-descent (IBD) sharing between Turkic groups and Eurasian reference populations. (**A**) Total IBD length shared between each Turkic group and Altaic, Sinitic, Tibeto-Burman, and Indo-European groups in the low-density extended HO dataset. (**B**) Summed length of IBD blocks over 10 cM (youngest) shared between each Turkic group and Altaic, Sinitic, Tibeto-Burman, and Indo-European groups in the low-density extended HO dataset. Eurasian reference populations are grouped by language family, with population name colors consistent with those in Figure S3. IBD length was stratified by the minimum value, the 50% median, and the 75%, 90%, 97.5%, and maximum percentiles.

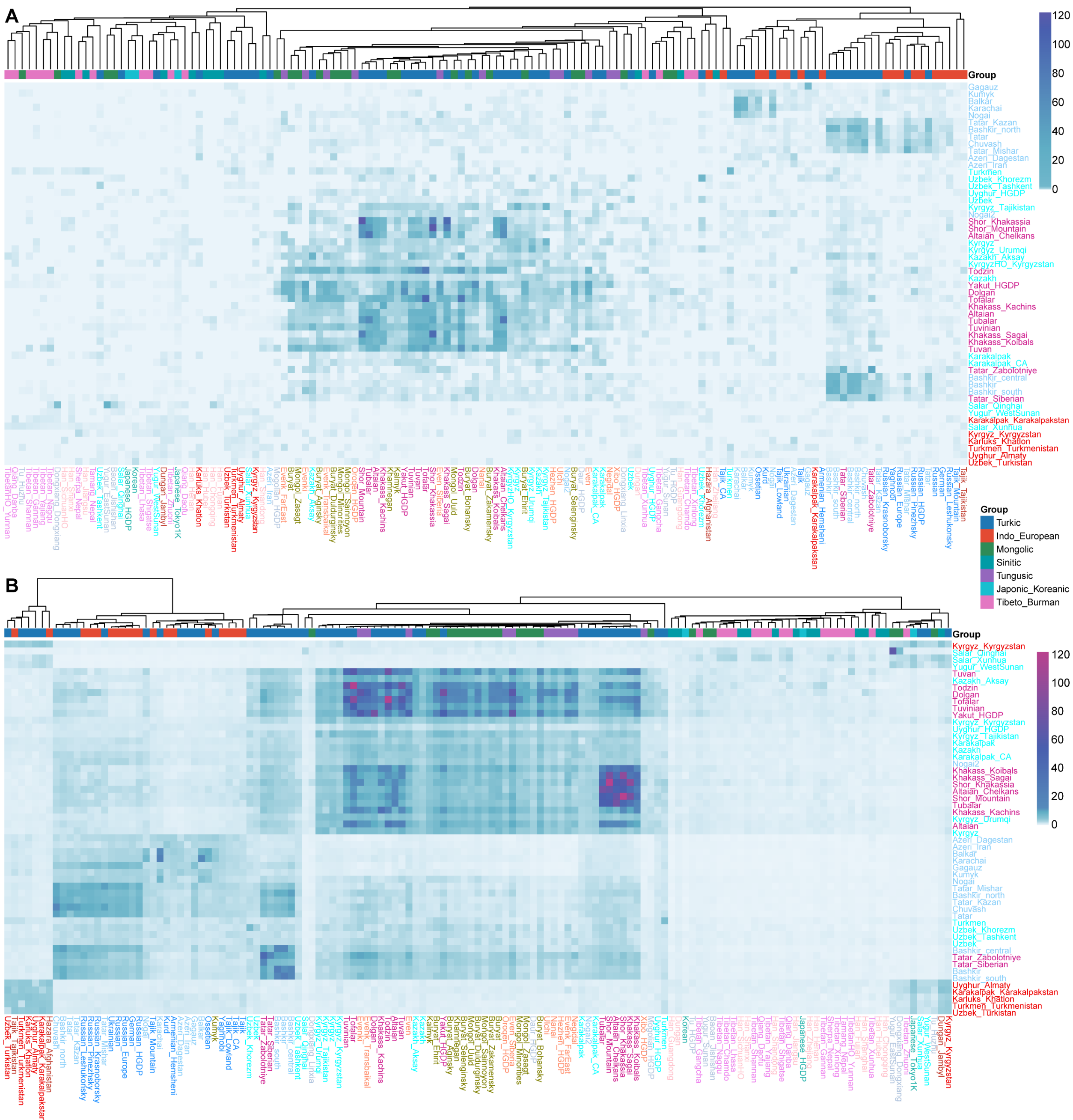

Figure S13. Summed lengths of IBD blocks between each Turkic group and Altaic, Sinitic, Tibeto-Burman, and Indo-European groups in the low-density extended HO dataset, shown

for block sizes of 5–10 cM (A) and 1–5 cM (B). Eurasian reference populations are grouped by language family, with population names colored consistently with those in Figure S3. IBD length was stratified by the minimum value, the 50% median, and the 75%, 90%, 97.5%, and maximum percentiles.

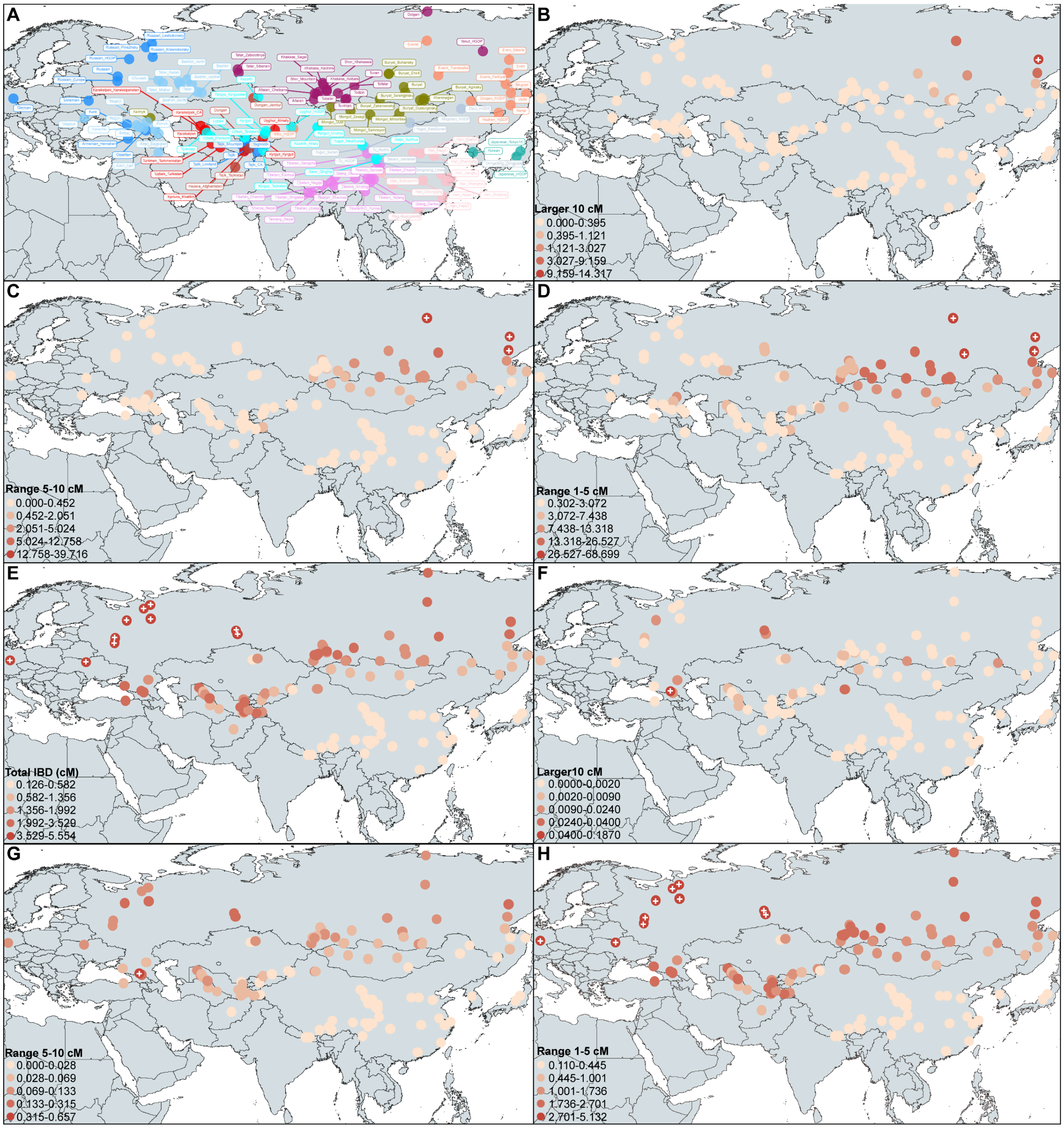

Figure S14. IBD sharing between Turkic groups and publicly available Altaic, Sino-Tibetan, and Indo-European groups. (**A**) Geographic distribution of reference populations used in the IBD analysis. Population colors follow those in Figure S3. (**B–D**) Summed length of IBD segments > 10 cM (**B**), 5–10 cM (**C**), and 1–5 cM (**D**) shared between the easternmost Turkic group (Dolgan and Yakut) and Eurasian reference populations in the low-density extended HO

dataset. (**E–H**) Summed length of total IBD lengths (**E**), IBD segments > 10 cM (**F**), 5–10 cM (**G**), and 1–5 cM (**H**) shared between 16 West Eurasian Turkic groups and Eurasian reference populations in the low-density extended HO dataset. Circle positions correspond to population distributions in Figure S14A.

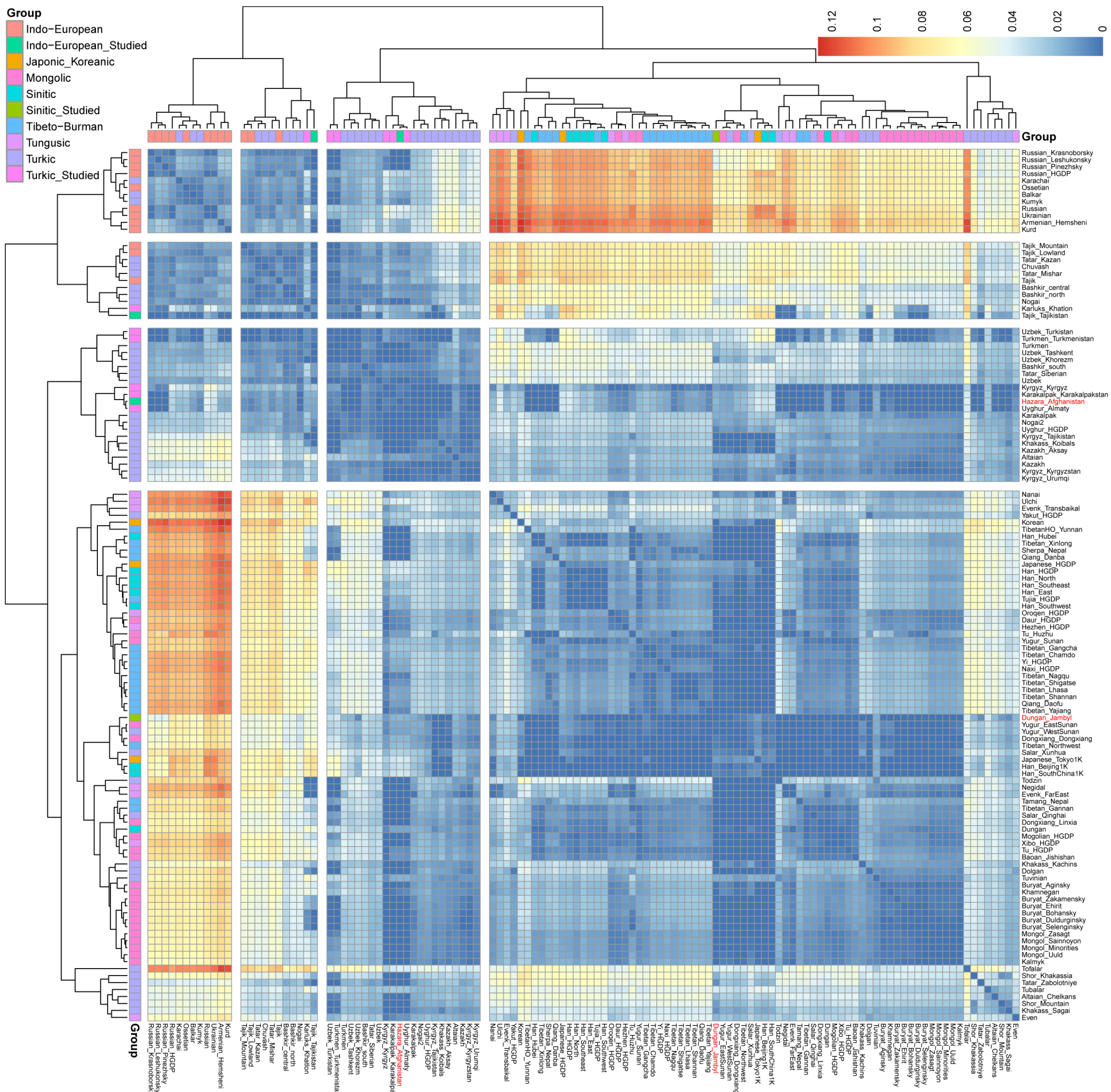

Figure S15. Pairwise Fst genetic distances between geographically distinct Eurasian populations.

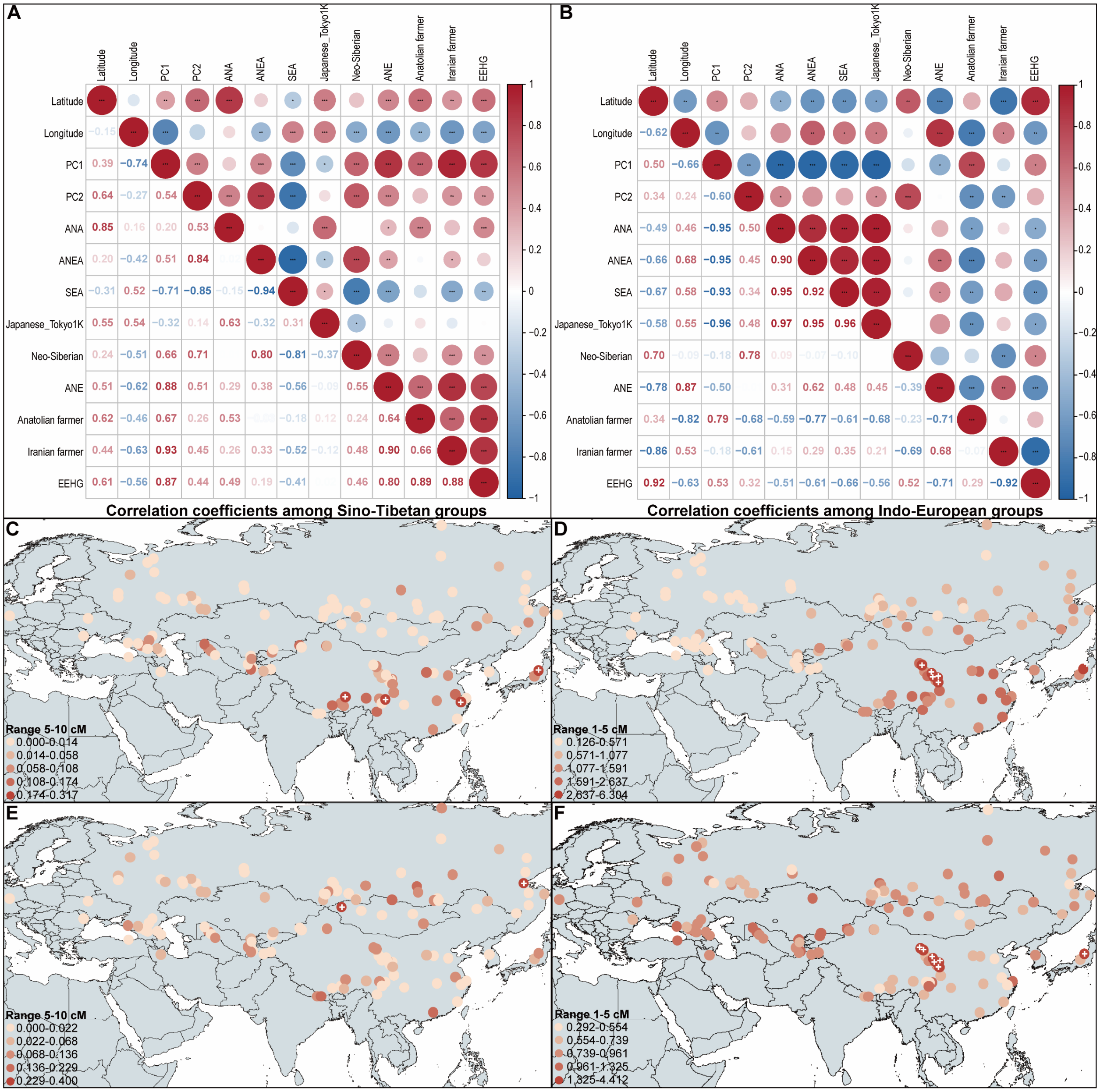

Figure S16. The correlation between geographical and genetic patterns and IBD sharing between Jambyl Dungan/Afghan Hazara and Eurasian reference populations. (**A–B**) The correlation between latitude/longitude and genetic features (PC1, PC2, and estimated ancestral components in Figure S5) and between estimated ancestral components among Sino-Tibetan groups across East Eurasia (**A**) and among Indo-European-speaking populations in Central Asia and West Eurasia (**B**). ANA: Ancient Northeast Asian ancestry; ANEA: Ancient northern East Asian ancestry; SEA: Southern East Asian ancestry; ANE: Ancient North Eurasian ancestry; EEHG: Eastern European hunter-gatherers. (**C–D**) Summed length of IBD segments 5–10 cM (**C**) and 1–5 cM (**D**) shared between the Jambyl Dungan and Eurasian reference populations in the low-density extended HO dataset. (**E–F**) Summed length of IBD segments 5–10 cM (**E**) and 1–5 cM (**F**) shared between the Afghan Hazara and Eurasian reference populations. Circle positions correspond to population distributions in Figure S14A.

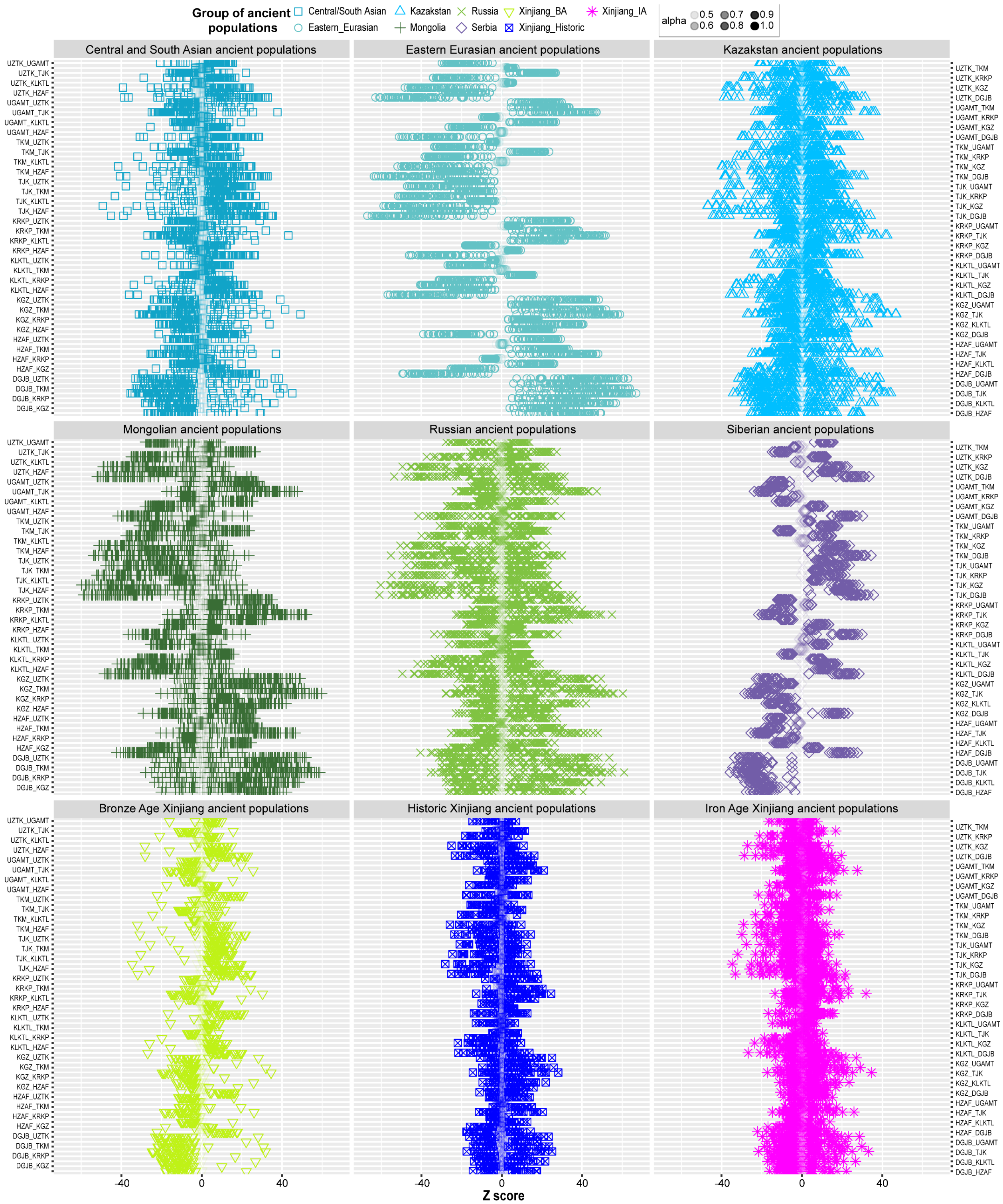

Figure S17. The Z scores of f_4-statistics of the form f_4(CAAH1, CAAH2; Reference, Mbuti). Ancient Eurasian groups were used as reference populations. Different colored shapes represent various groups of ancient reference populations. DGJB: Dungan_Jambyl, HZAF: Hazara_Afghanistan, UZTK: Uzbek_Turkistan, UGAMT: Uyghur_Almaty, TJK: Tajik_Tajikistan, KLKTL: Karluks_Khatlon, TKM: Turkmen_Turkmenistan, KRKP: Karakalpak_Karakalpakstan, and KGZ: Kyrgyz_Kyrgyzstan.

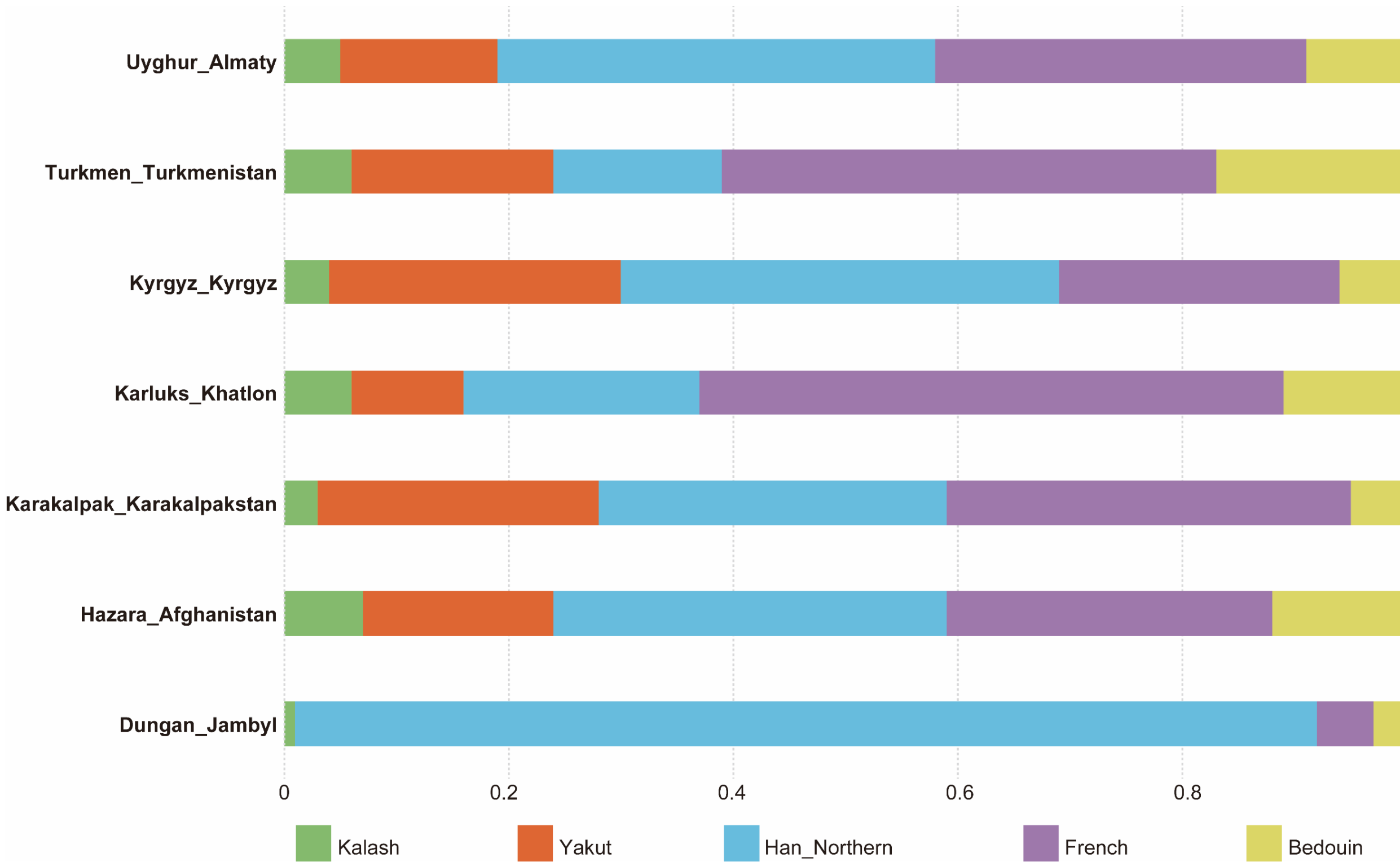

Figure S18. The admixture profiles of CAAH estimated based on SOURCEFIND.

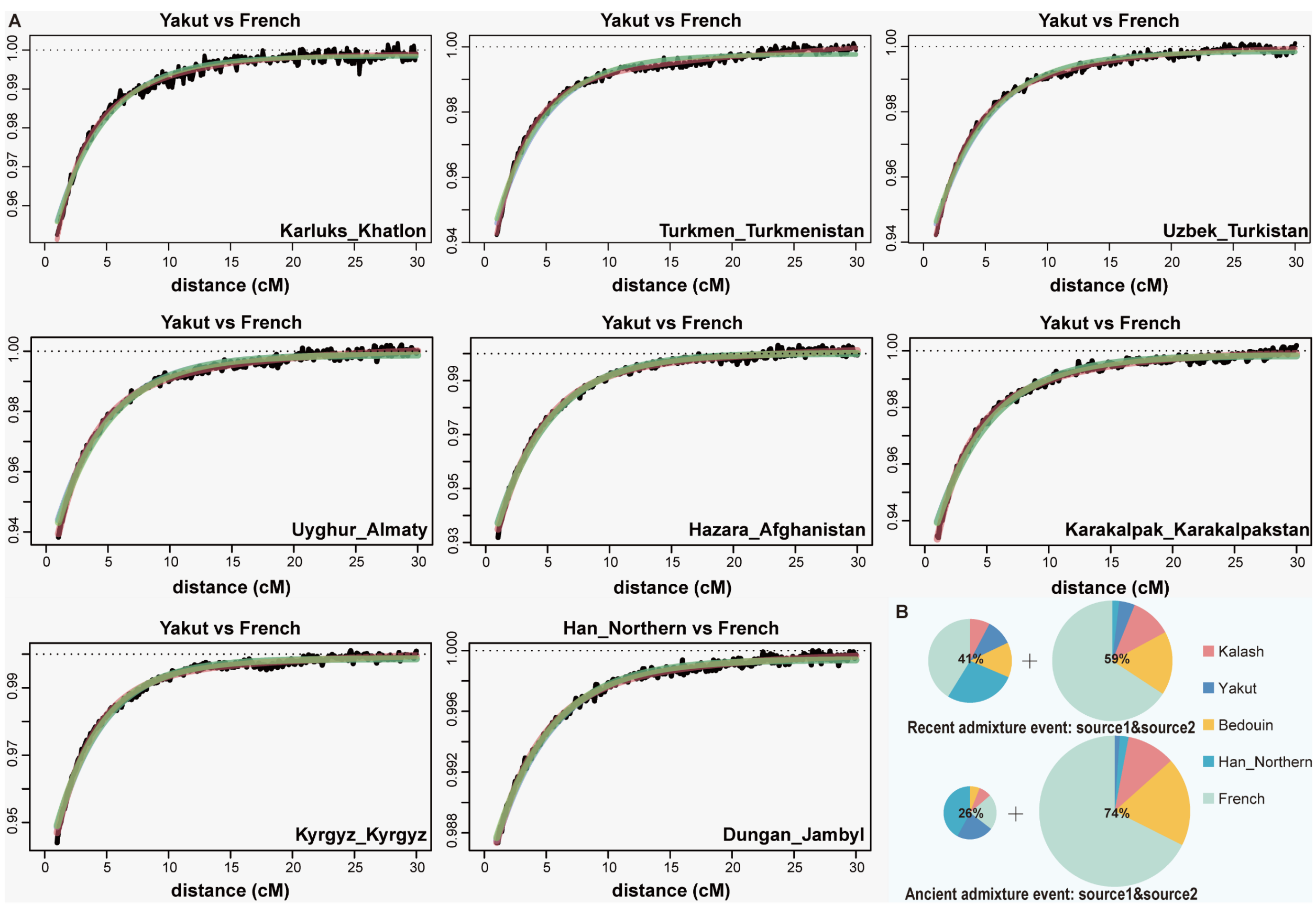

Figure S19. The ancestry patterns of CAAH revealed via haplotype-based

fastGLOBETROTTER. (**A**) Admixture probability curves for CAAH except for the Tajikistan Tajik. The black line represents the scaled probability, the green line shows the fitted model assuming a single wave of admixture, the cyan line shows the fitted model assuming a single wave of admixture between two source populations, and the red line shows the fitted model assuming two waves of admixture with different dates. (**B**) The ancestry profile of Tajikistan Tajik estimated with the original surrogate populations.

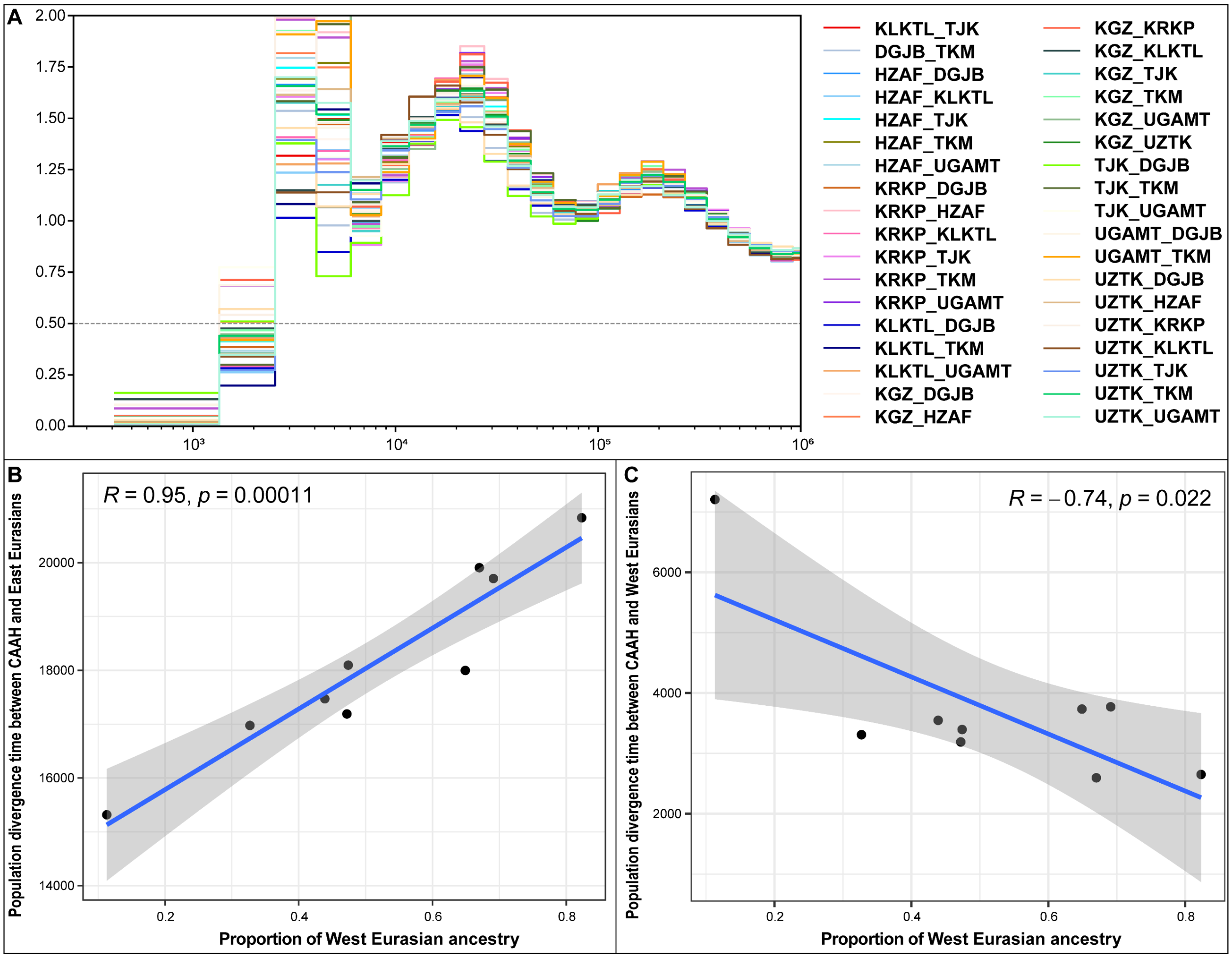

Figure S20. Population divergence times among CAAH groups. (**A**) The inferred population split times of pairwise CAAH groups. (**B**) The correlation between the estimated proportion of West Eurasian ancestry and population divergence times of CAAH vs East Eurasian population, represented by Northern Han Chinese. (**C**) The correlation between the estimated proportion of West Eurasian ancestry and population divergence times of CAAH vs West Eurasian population, represented by French.

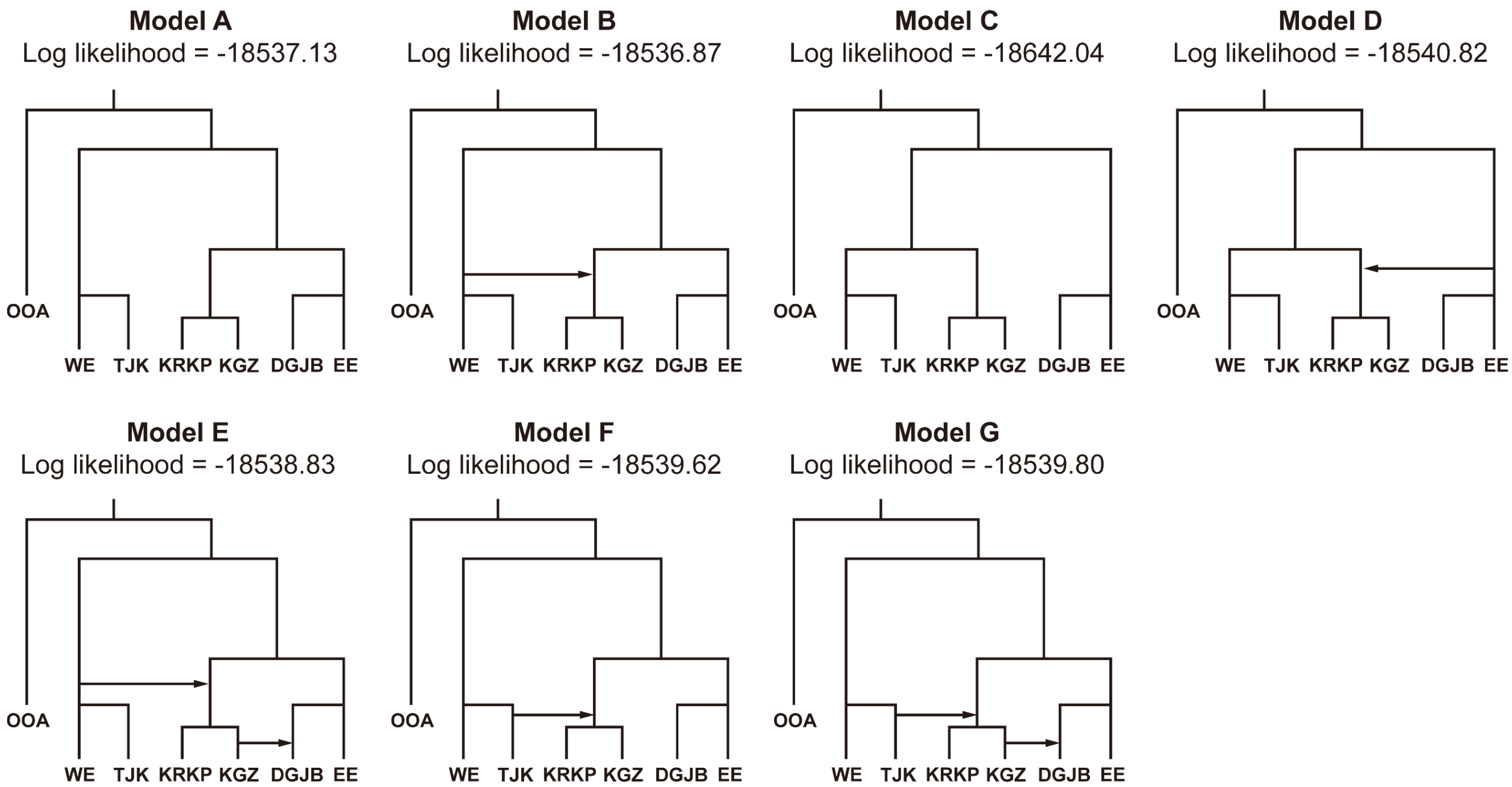

Figure S21. Maximum-likelihood demographic models for Central Asian populations. Model A provided the best overall fit, despite a slightly lower likelihood than Model B, because the likelihood difference was not statistically significant and Model A used fewer parameters. OOA: Out of Africa; WE: West Eurasian; TJK: Tajik_Tajikistan; KRKP: Karakalpak_Karakalpakstan; KGZ: Kyrgyz_Kyrgyzstan; DGJB: Dungan_Jambyl; EE: East Eurasian.

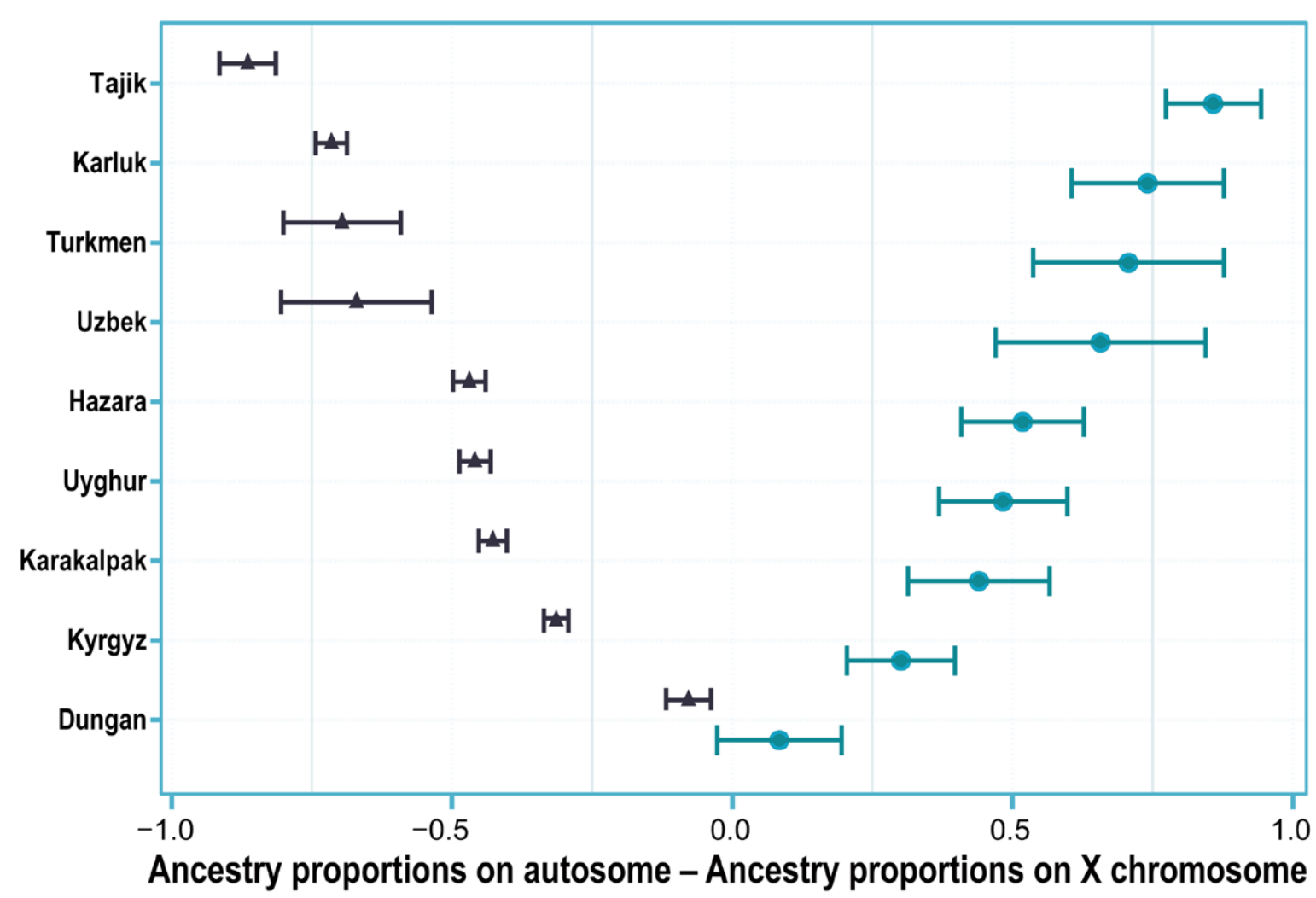

Figure S22. Sex-biased admixture patterns in CAAH inferred from autosomal and X-chromosomal variants. The estimated proportions of western Eurasian ancestry are shown, with autosomal-derived values plotted as negative values.

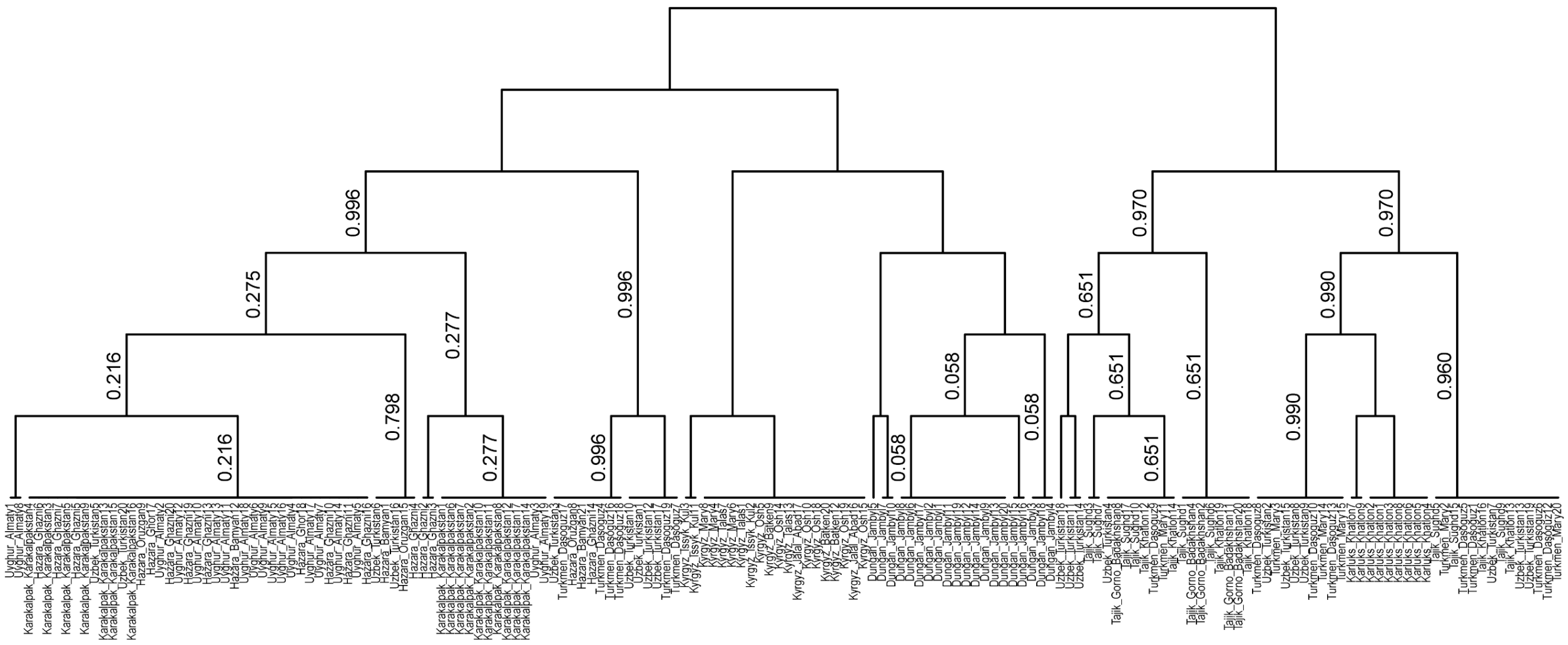

Figure S23. Fine-scale population structure of the newly sequenced populations as revealed by fineSTRUCTURE analysis.

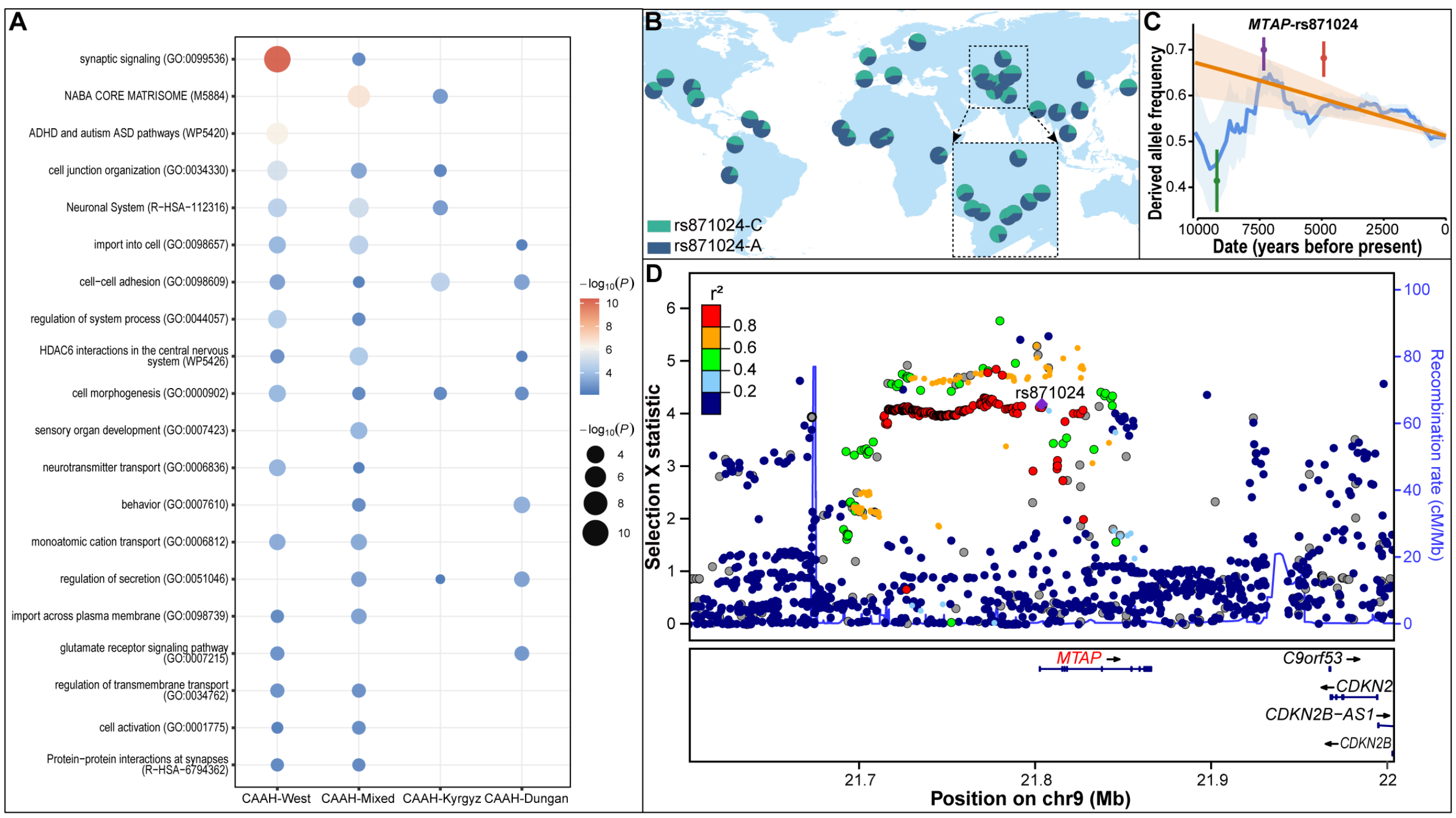

Figure S24. Enrichment analysis of natural selection signals identified using a window-based scan and the distribution patterns of *MTAP*-rs871024 frequencies in ancient and modern populations. (**A**) The heatmap presents biological pathways associated with candidate signatures of local adaptation. (**B**) Allele frequency distributions of *MTAP*-rs871024 across global populations. (**C**) The derived allele frequency (DAF) trajectory over time for *MTAP*-rs871024. (**D**) Selection plot of *MTAP* variants and linkage patterns of *MTAP*-rs871024 identified in CAAH-Mixed with adjacent loci. The plot was generated using selection X-statistic values derived from ancient West Eurasian populations (Harvard Dataverse: https://doi.org/10.7910/DVN/7RVV9N).

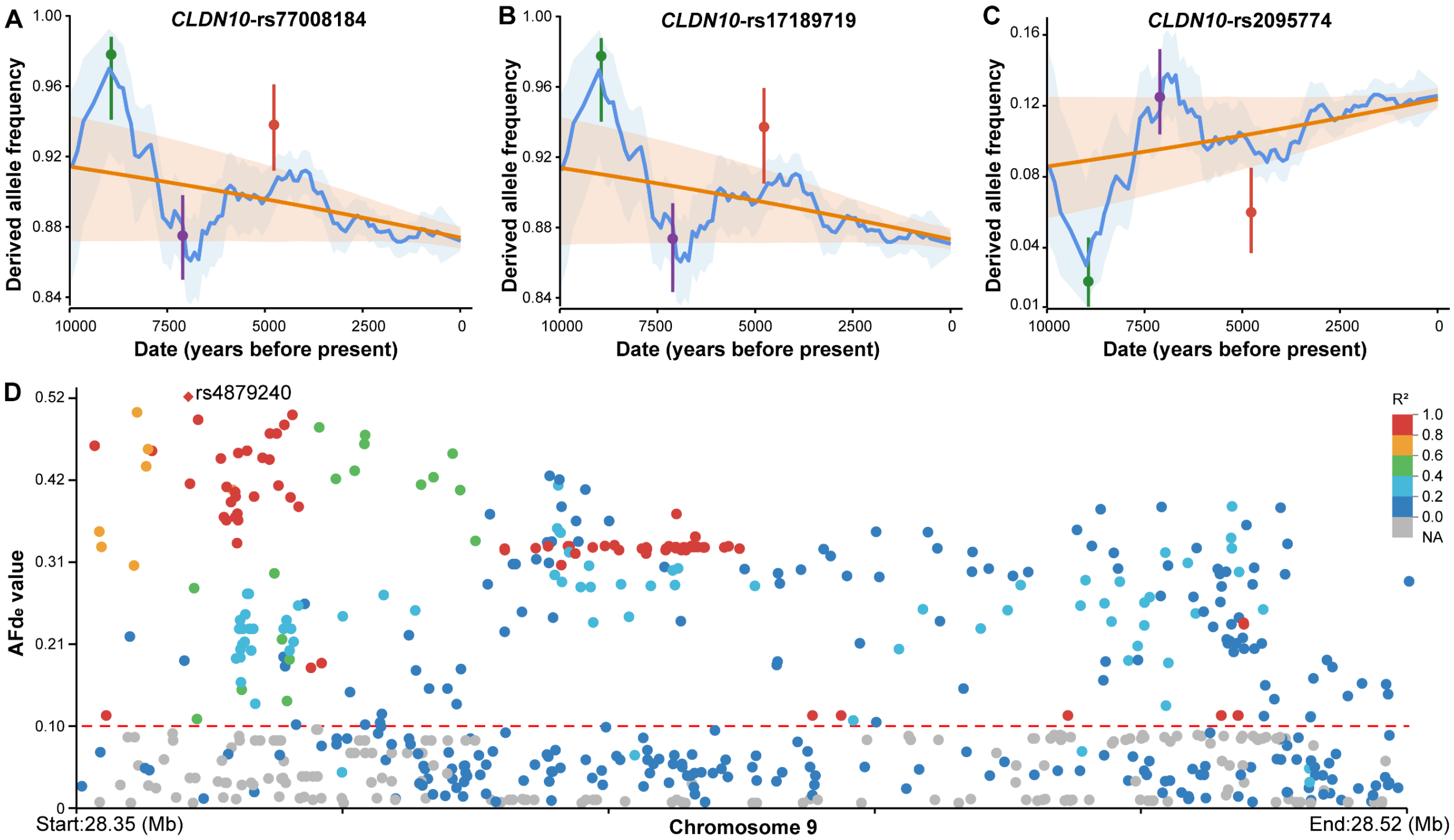

Figure S25. DAF trajectories of variants within *MTAP* and linkage patterns of variants within *TRAF1*. (**A–C**) The DAF trajectory over time for *GLDN10*-rs77008184 (**A**), *GLDN10*-rs17189719 (**B**), and *GLDN10*-rs2095774 (**C**). (**D**) Linkage patterns of variants within the *LINGO2* gene, with rs4879240 exhibiting the highest AFd_e in natural selection signals within this region as the focal locus. The positions of the variants in Figure S25D are the same as in Figure 4E. Different degrees of linkage disequilibrium (LD) are represented by various colors, with red ($R^2 = 1$) indicating complete LD. AFd_e: the deviation between observed and expected allele frequencies.

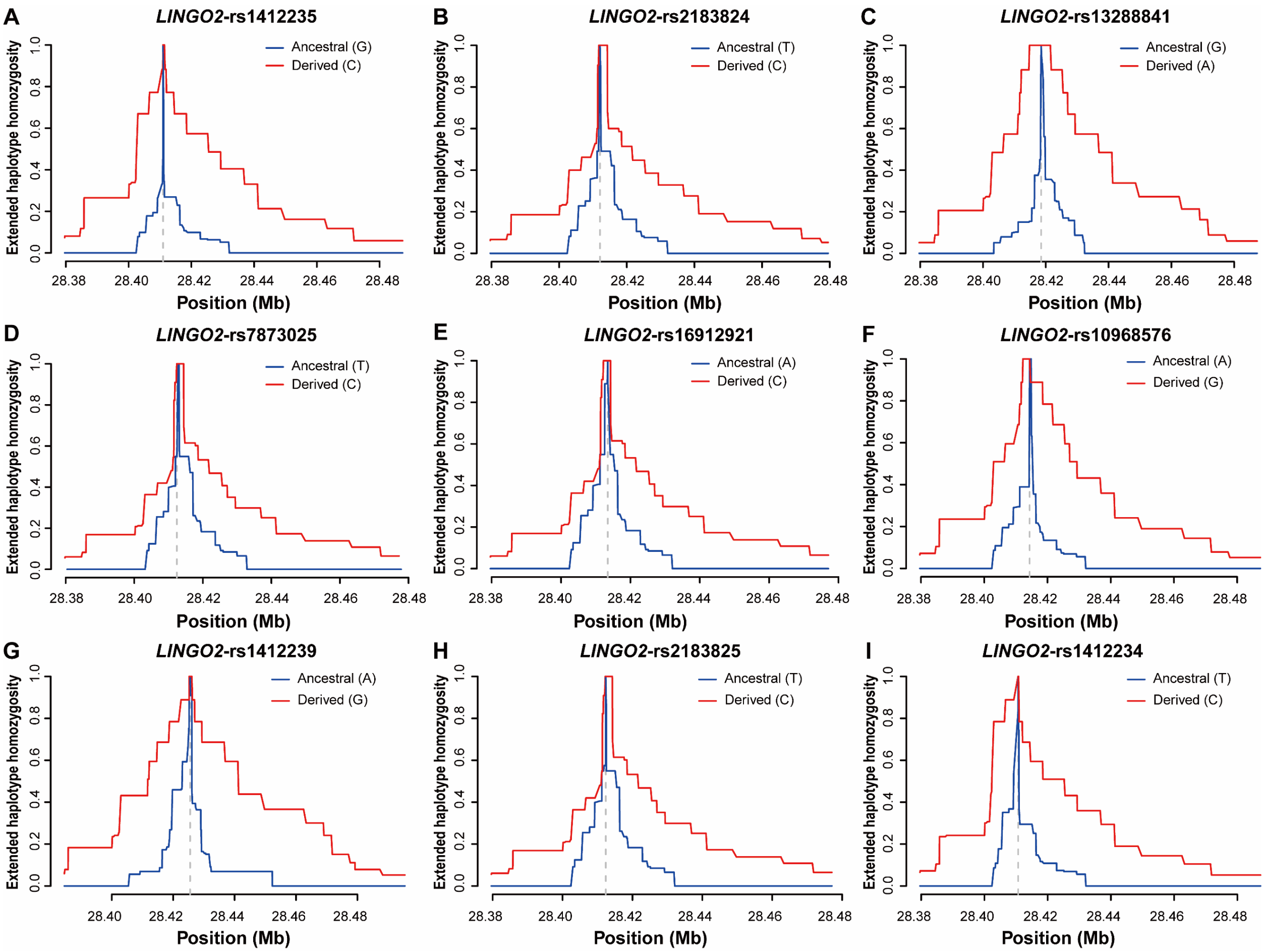

Figure S26. Extended haplotype homozygosity (EHH) curves of *LINGO2* variants. The EHH curves of rs1412235 (**A**), rs2183824 (**B**), rs13288841 (**C**), rs7873025 (**D**), rs16912921 (**E**), rs10968576 (**F**), rs1412239 (**G**), rs2183825 (**H**), and rs1412234 (**I**) in the *LINGO2* gene.

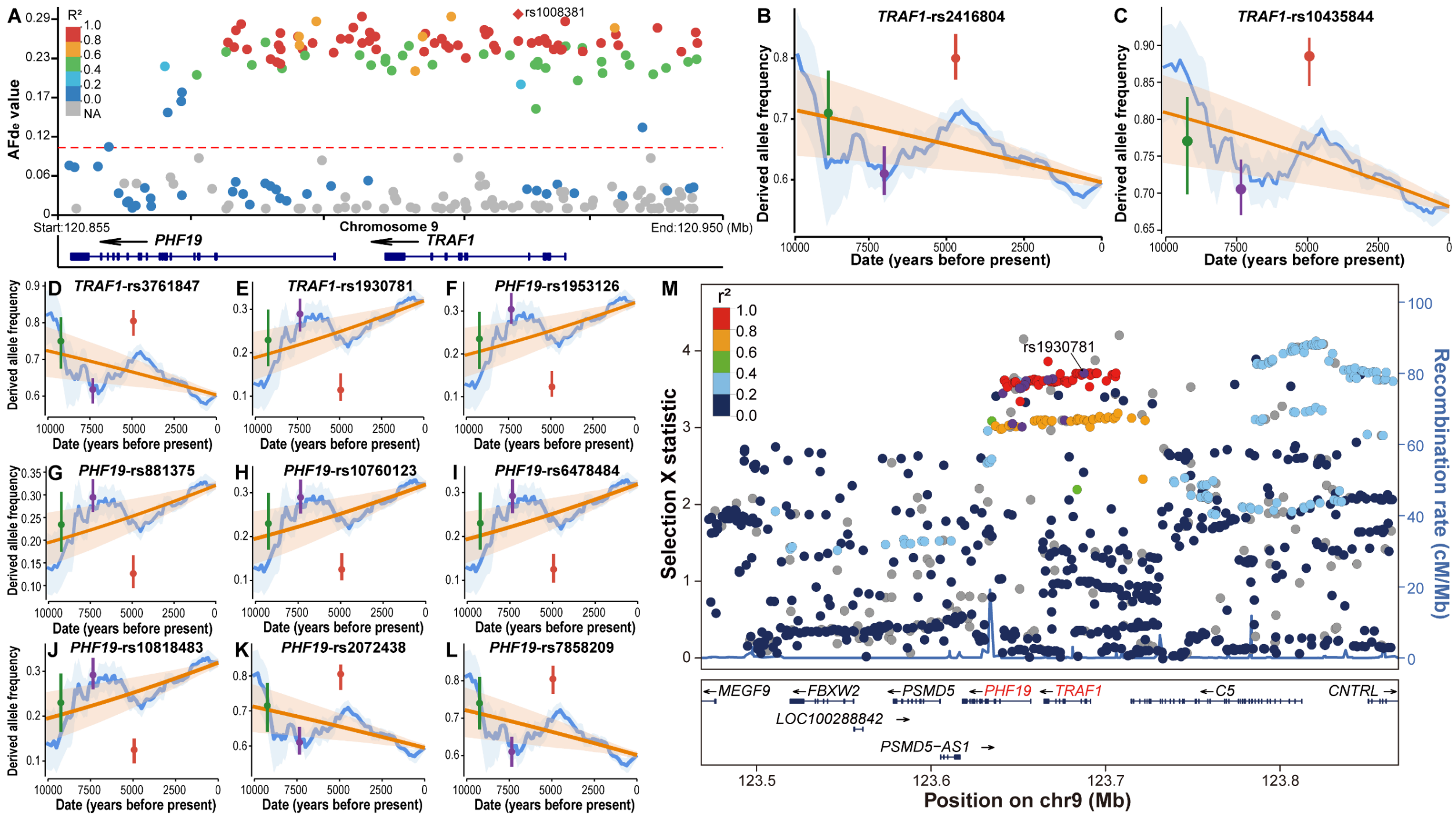

Figure S27. Linkage patterns, DAF trajectories, and selection signals for *TRAF1* and *PHF19* variants. (**A**) Linkage patterns of variants within the *TRAF1* and *PHF19* genes, with rs1008381 exhibiting the highest AFdₑ in natural selection signals within this region as the focal locus. The positions of the variants in Figure S27A are the same as in Figure 4F. Different degrees of LD are represented by various colors, with red ($R^2 = 1$) indicating complete LD. (**B–E**) The DAF trajectory over time for rs2416804 (**B**), rs10435844 (**C**), rs3761847 (**D**), and rs1930781 (**E**) in the *TRAF1* gene. (**F–L**) The DAF trajectory over time for rs1953126 (**F**), rs881375 (**G**), rs10760123 (**H**), rs6478484 (**I**), rs10818483 (**J**), rs2072438 (**K**), and rs7858209 (**L**) in the *PHF19* gene. (**M**) Selection plot of *TRAF1* and *PHF19* variants among ancient West Eurasian populations, alongside linkage patterns within the target region centered on rs1930781, which demonstrated the highest AFdₑ among natural selection signals identified for *TRAF1* and *PHF19* variants with phenotypic effects reported in the GWAS Catalog. The plot was generated via selection X-statistic values derived from ancient West Eurasian populations (Harvard Dataverse: https://doi.org/10.7910/DVN/7RVV9N).

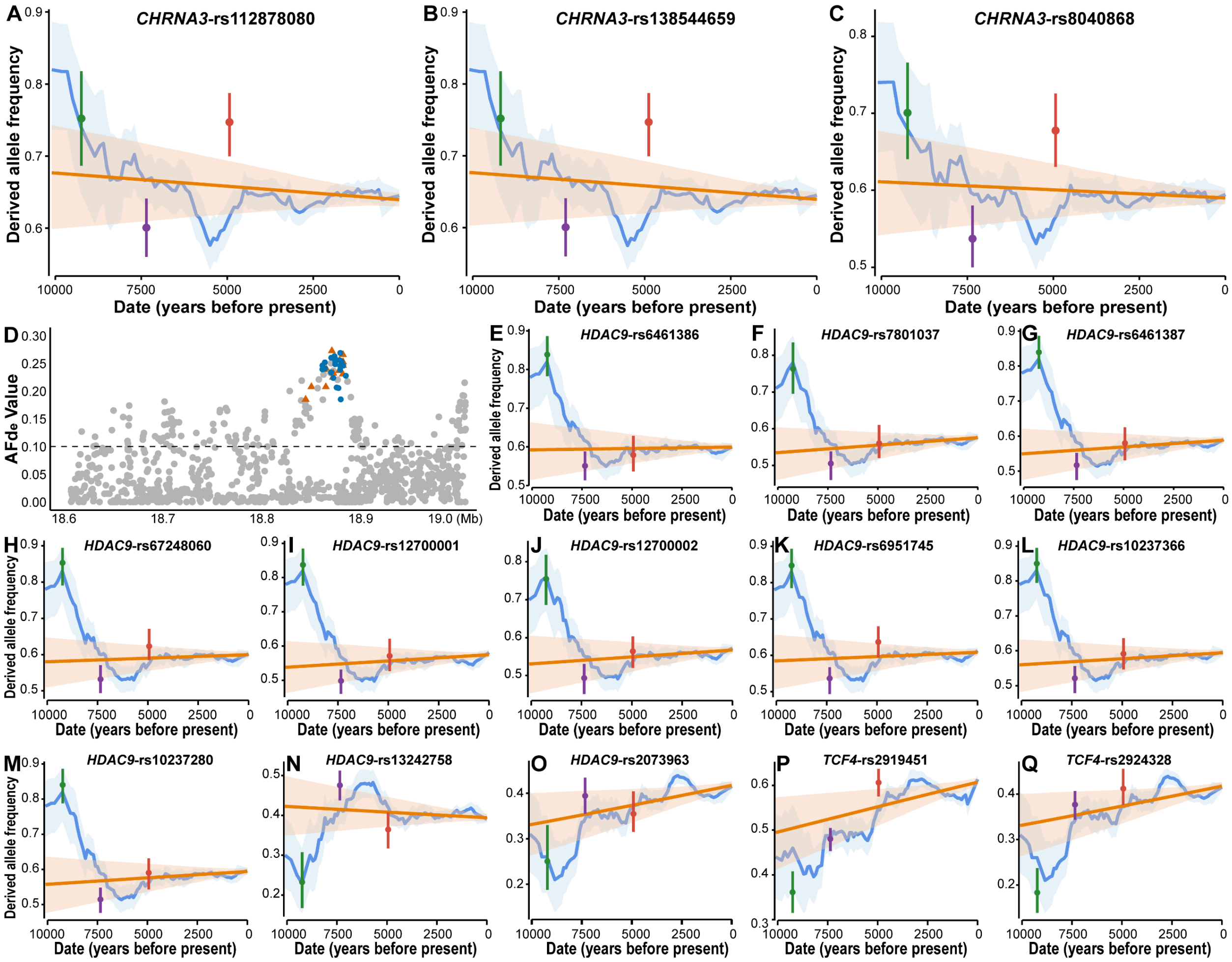

Figure S28. DAF trajectories of variants within *CHRNA3* and *HDAC9* and AFd$_e$ distribution of variants within *HDAC9*. (**A–C**) DAF trajectories over time for *CHRNA3*-rs112878080 (**A**), *CHRNA3*-rs138544659 (**B**), and *CHRNA3*-rs8040868 (**C**), respectively. (**D**) AFd$_e$ distribution of variants located between 18.6 and 19.0 Mb within *HDAC9*. Variants in blue indicate natural selection signals without reported phenotypic effects, whereas those in orange represent natural selection signals with phenotypic associations reported in the GWAS Catalog. DAF trajectories over time for rs6461386, rs7801037, rs6461387, rs67248060, rs12700001, rs12700002, rs6951745, rs10237366, rs10237280, rs13242758, and rs2073963 in *HDAC9* (**E–O**), as well as rs2919451 and rs2924328 in *TCF4* (**P–Q**).

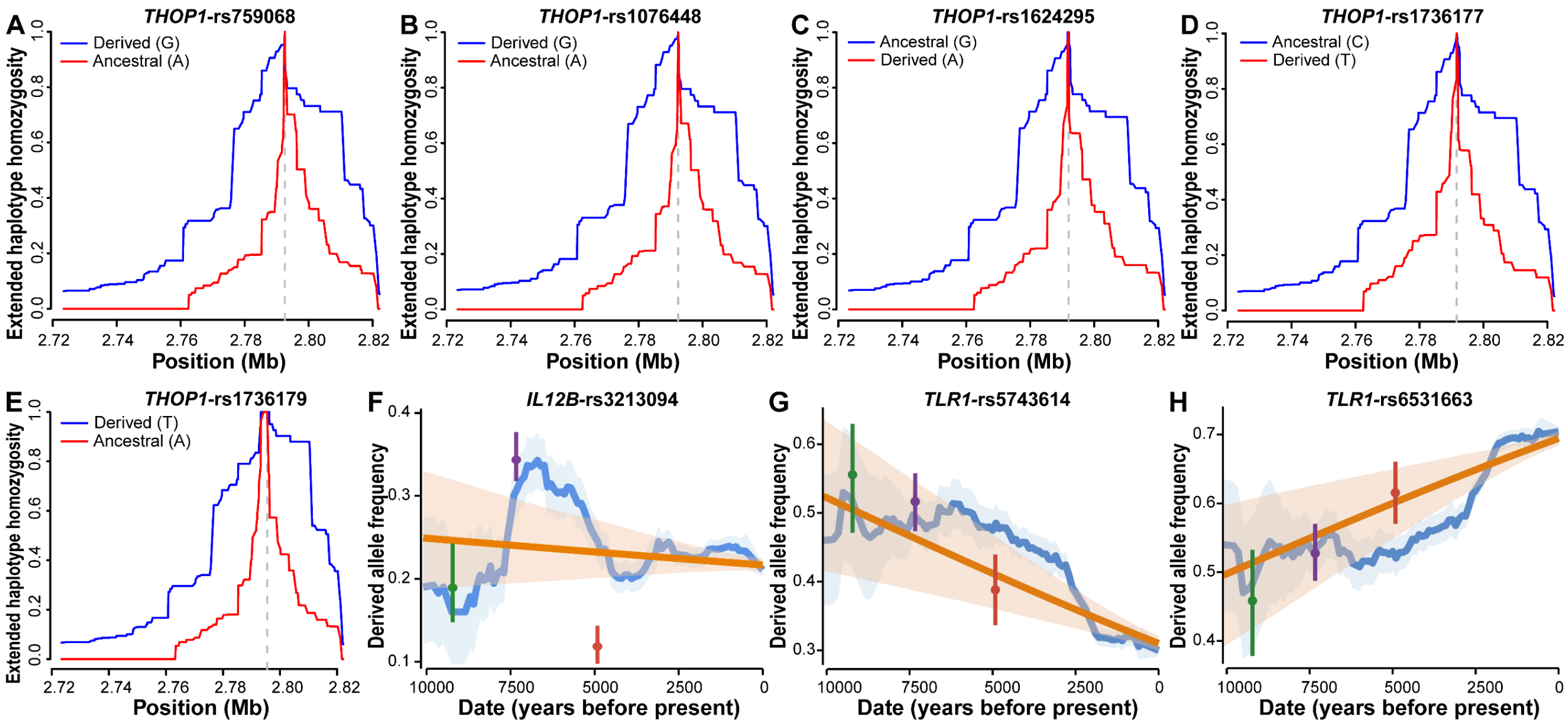

Figure S29. EHH curves of *THOP1* variants and DAF trajectories of variants in the *IL12B* and *TLR1* genes. (**A–E**) The EHH curves of rs759068 (**A**), rs1076448 (**B**), rs1624295 (**C**), rs1736177 (**D**), and rs1736179 (**E**) in the *THOP1* gene. (**F–H**) The DAF trajectory over time for *IL12B*-rs3213094 (**F**), *TLR1*-rs5743614 (**G**), and *TLR1*-rs6531663 (**H**).

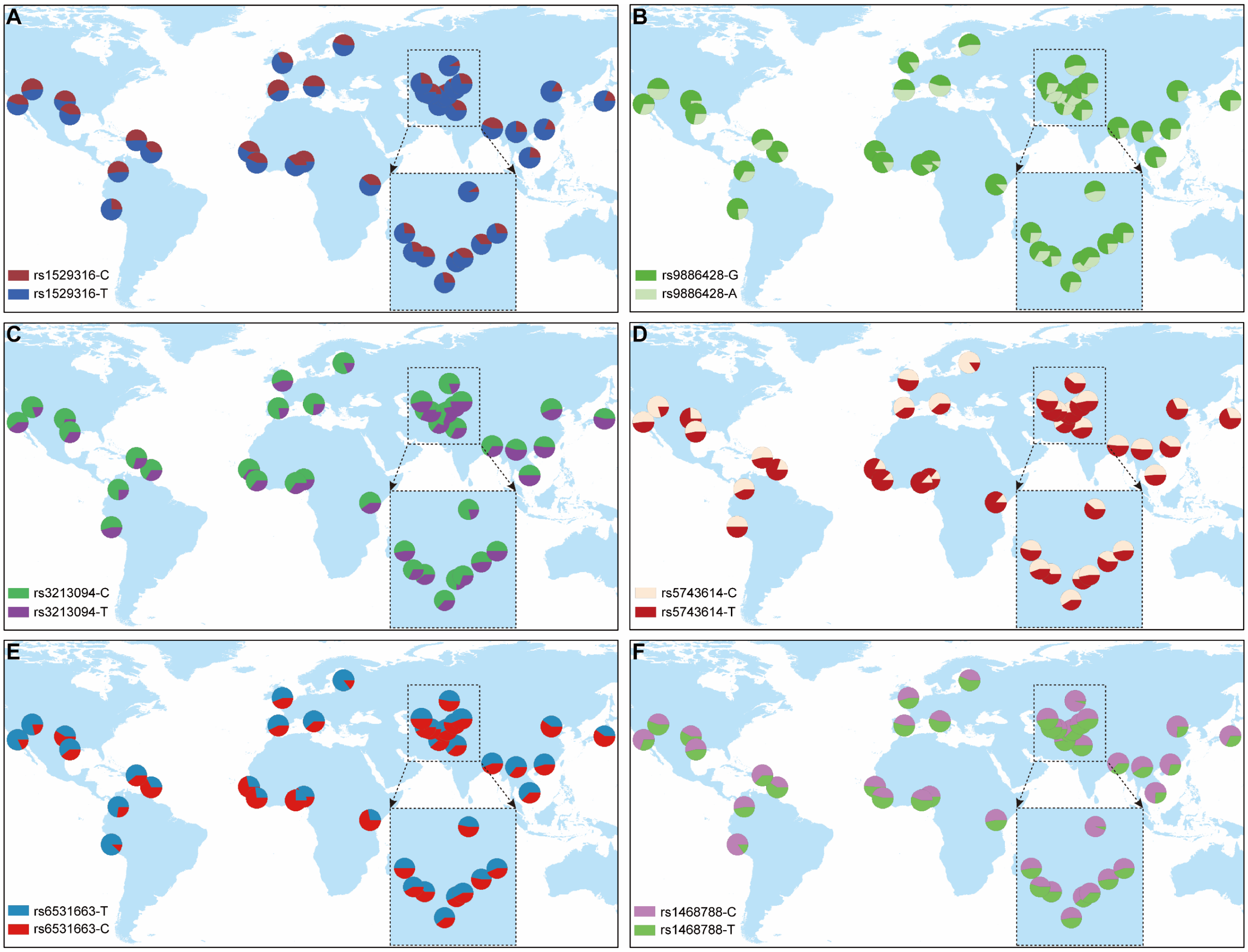

Figure S30. Geographical distribution of allele frequencies of natural selection signals associated with immune-related conditions. The allele frequency distributions of *CSMD1*-rs1529316 (**A**), *SGCZ*-rs9886428 (**B**), *IL12B*-rs3213094 (**C**), *TLR1*-rs5743614 (**D**), *TLR1*-rs6531663 (**E**), and *SLC9A4*-rs1468788 (**F**).

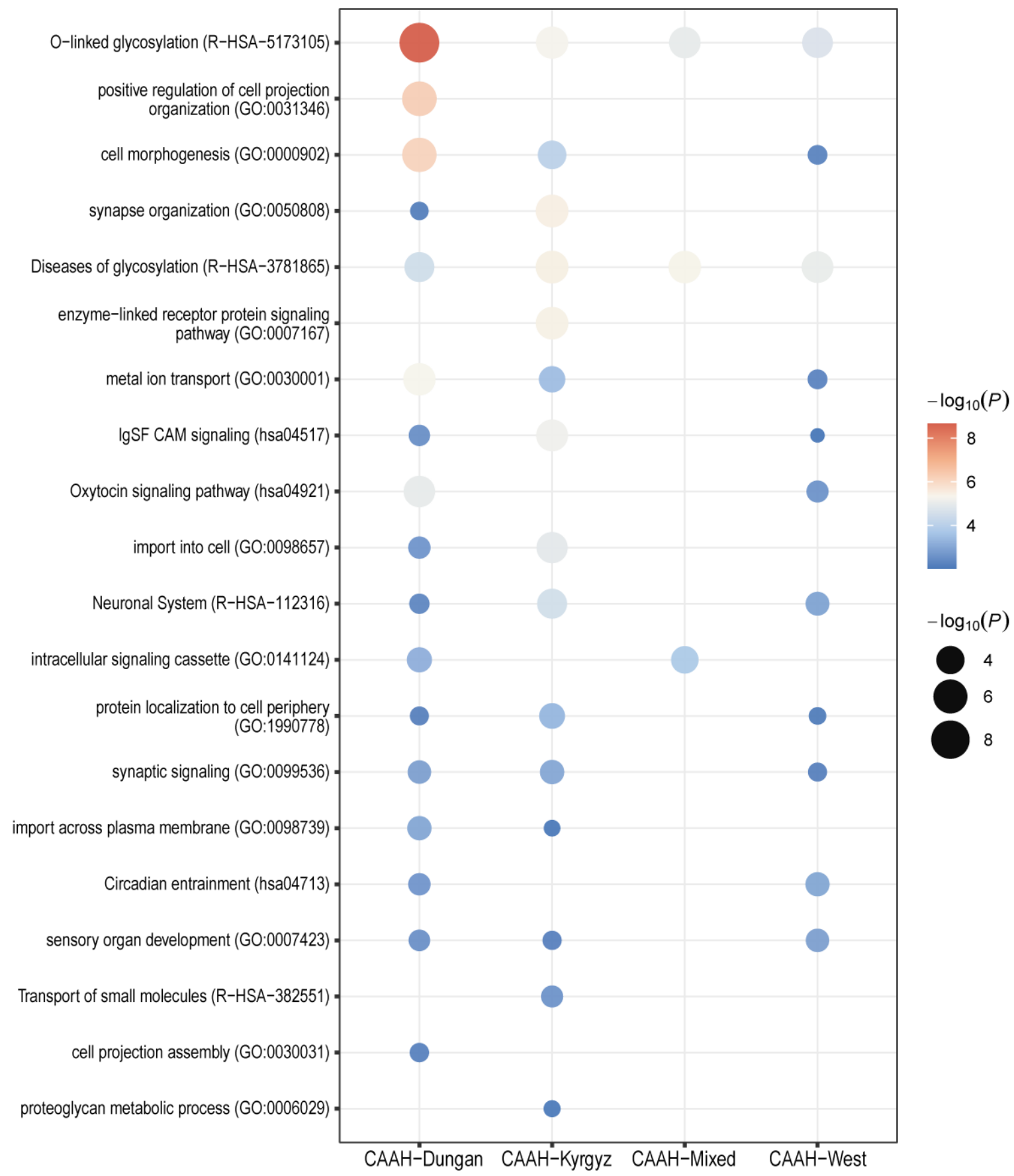

Figure S31. Enrichment analysis of natural selection signals identified using the composite of multiple signals (CMS).

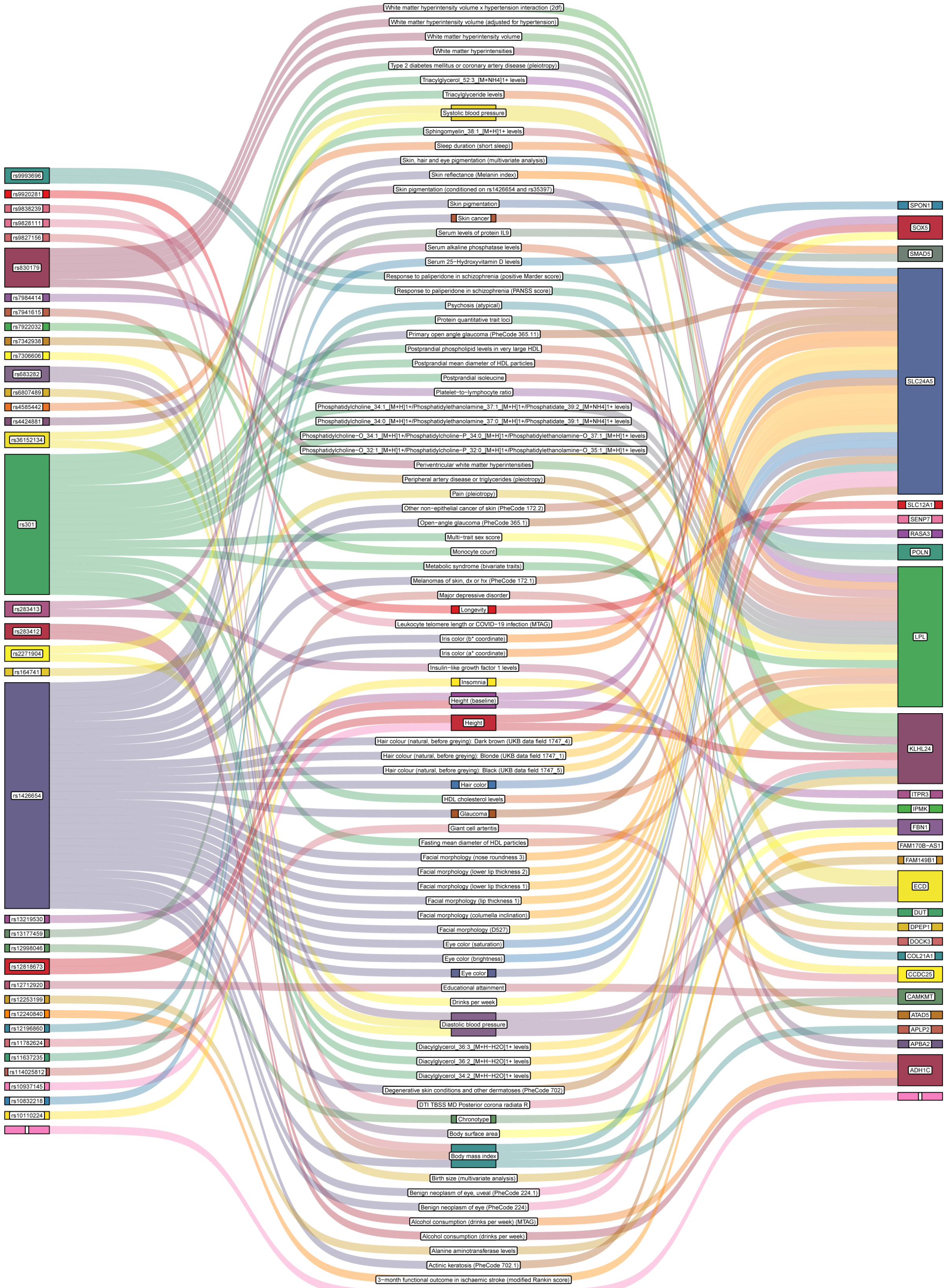

Figure S32. Genome-wide association study (GWAS) annotation of natural selection signals identified by CMS in CAAH-West.

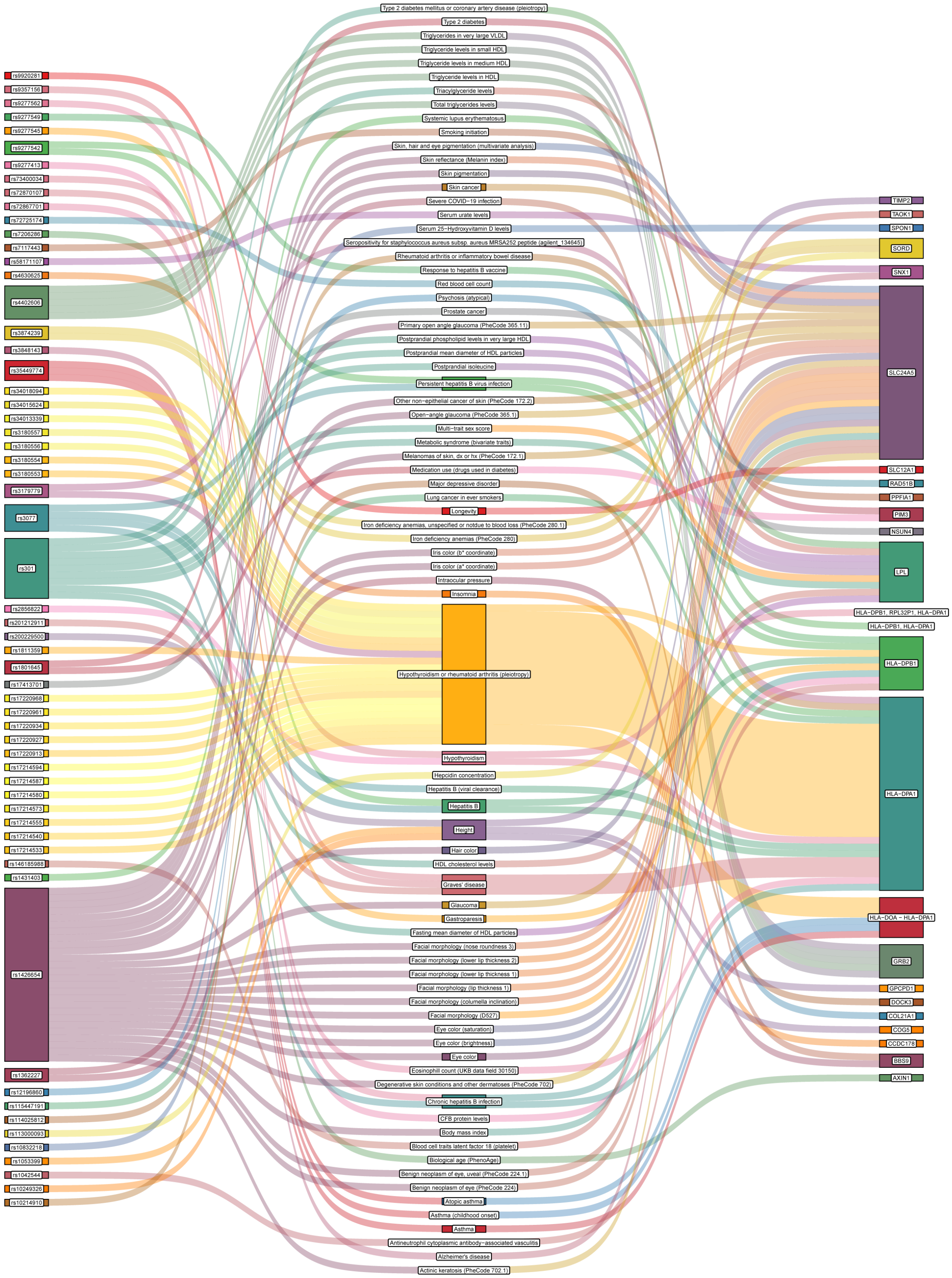

Figure S33. GWAS annotation of natural selection signals identified by CMS in CAAH-Mixed.

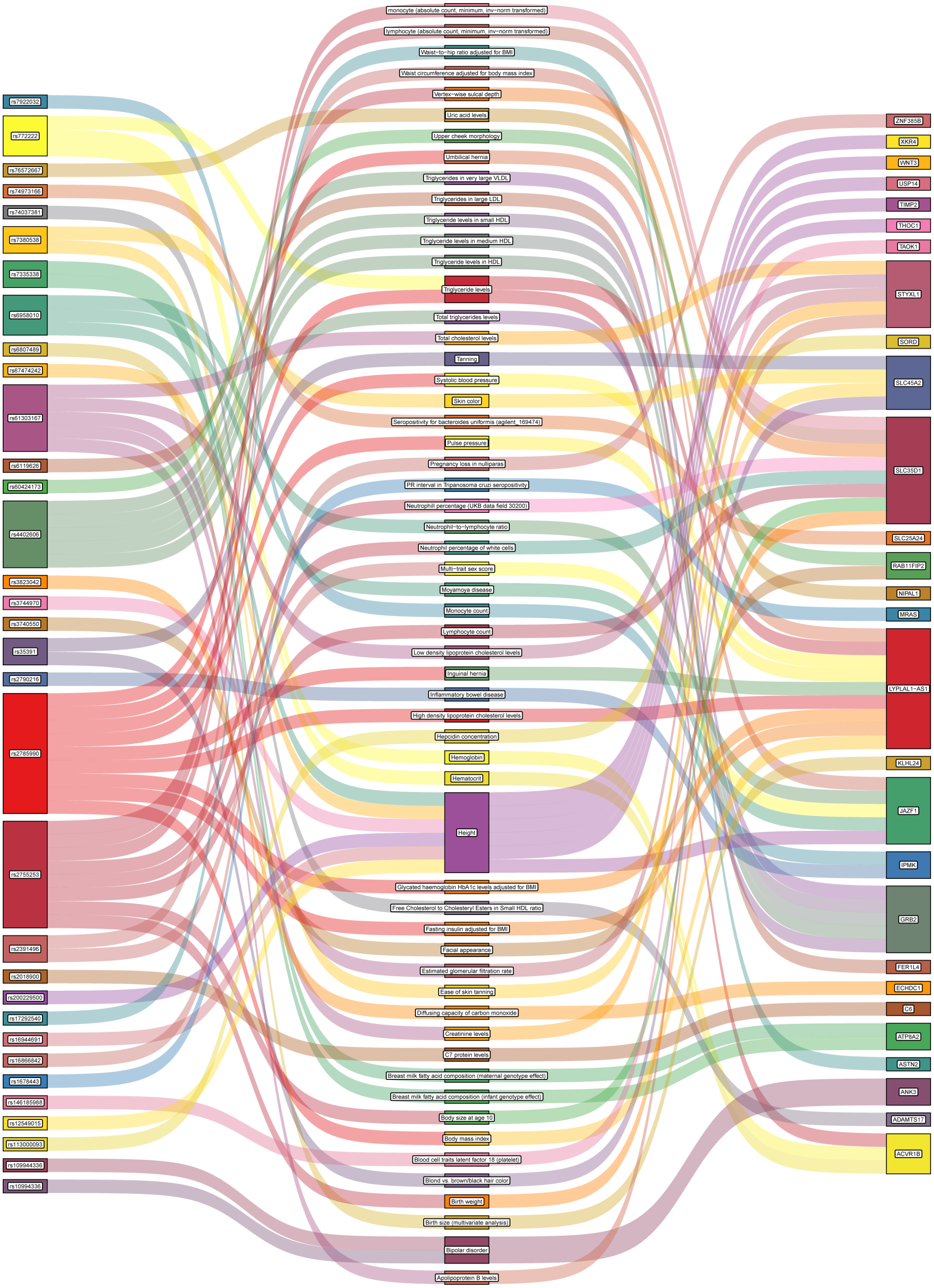

Figure S34. GWAS annotation of natural selection signals identified by CMS in CAAH-Kyrgyz.

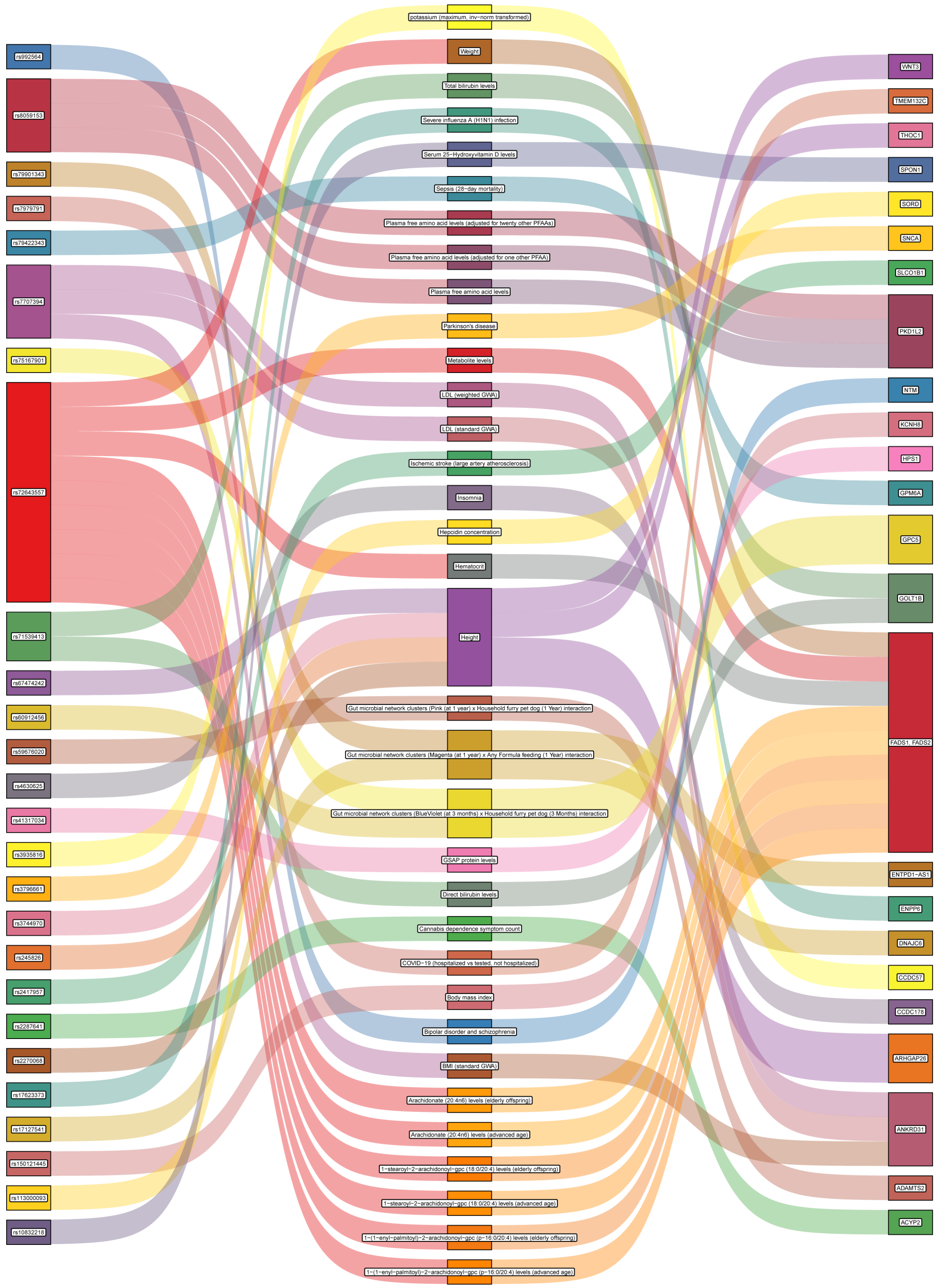

Figure S35. GWAS annotation of natural selection signals identified by CMS in CAAH-Dungan.

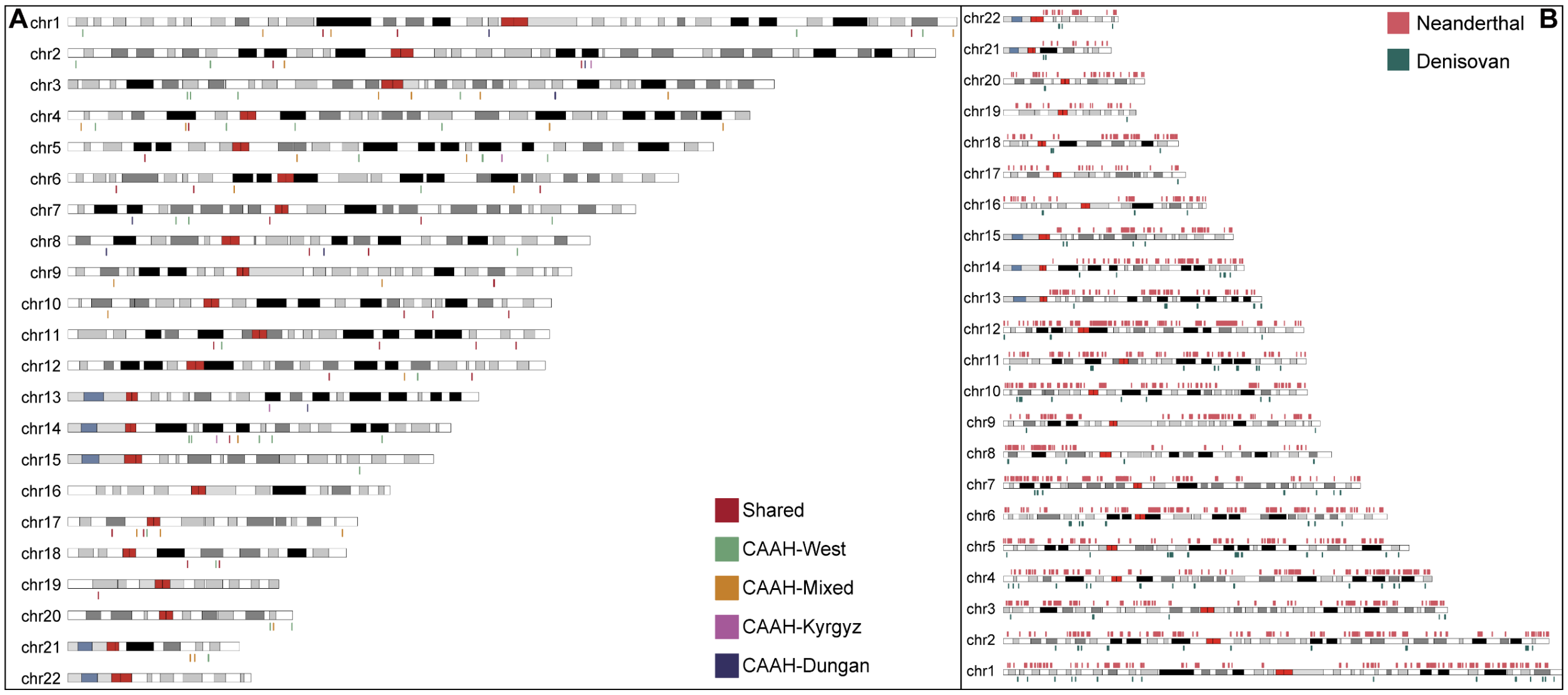

Figure S36. Distribution of Neanderthal- and Denisovan-derived archaic introgression segments (AISs). (**A**) Distribution of Neanderthal-derived AISs unique to CAAH groups and absent from Eurasian reference populations in the 1000 Genomes Project. AISs shared across the four CAAH subgroups are depicted in dark red, whereas those specific to CAAH-West, CAAH-Mixed, CAAH-Kyrgyz, and CAAH-Dungan are represented in dark green, orange, purple, and dark blue, respectively. (**B**) Distribution of high-confidence Neanderthal- and Denisovan-derived AISs across 22 autosomes. High-confidence Neanderthal-derived AISs were identified by intersecting results from IBDmix and Sprime.

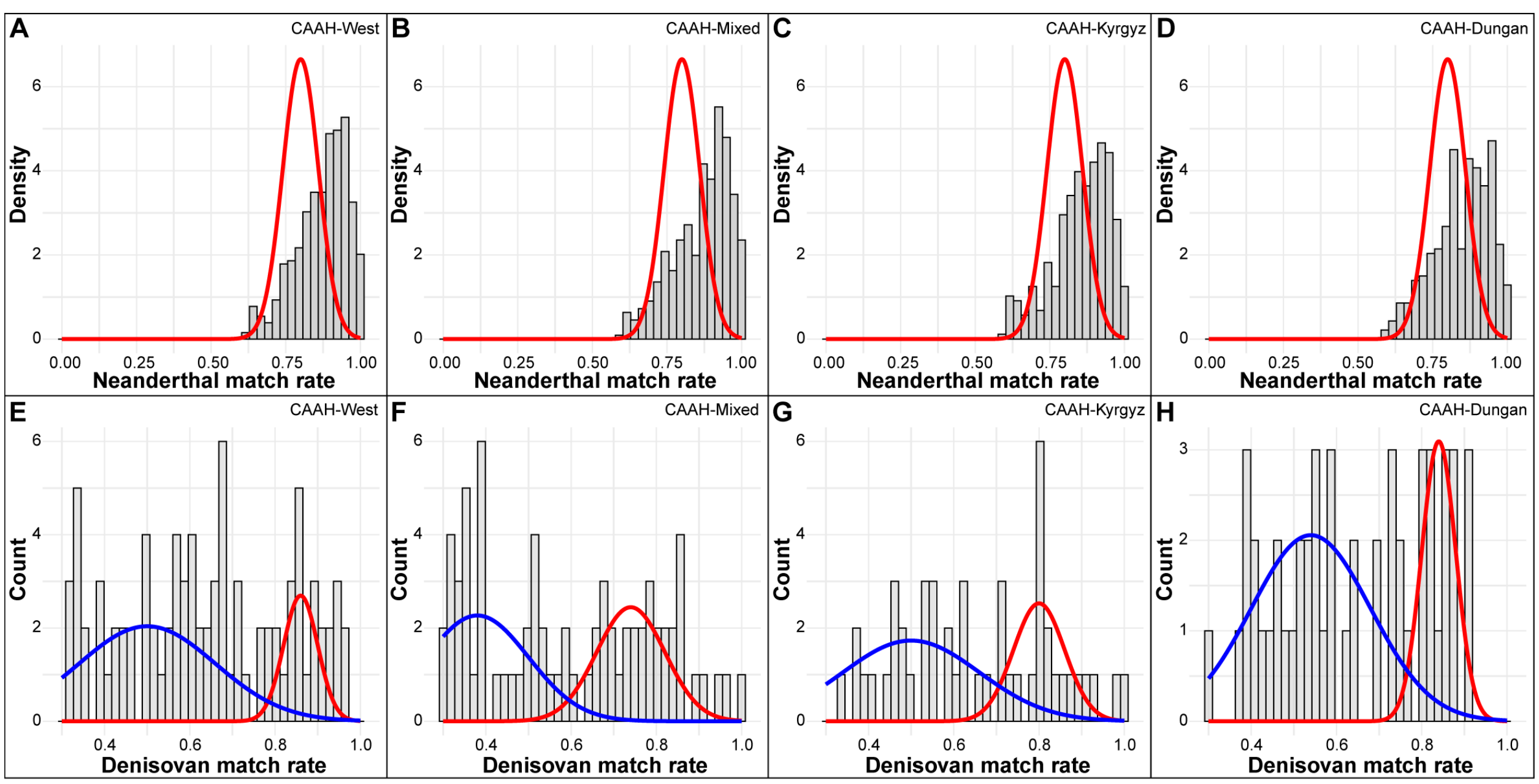

Figure S37. Distributions of Neanderthal and Denisovan match rates. Histogram of Neanderthal match rates for CAAH-West (**A**), CAAH-Mixed (**B**), CAAH-Kyrgyz (**C**), and CAAH-Dungan (**D**). The normal distribution for the best-fitting parameters for the high-affinity Neanderthal component (mu1, sd1) is shown for each. Histogram of Denisovan match rates for CAAH-West (**E**), CAAH-Mixed (**F**), CAAH-Kyrgyz (**G**), and CAAH-Dungan (**H**). The normal distribution for the best-fitting parameters for the high-affinity Denisovan component (mu1, sd1)

and low-affinity Denisovan component (mu2, sd2) is shown for each.

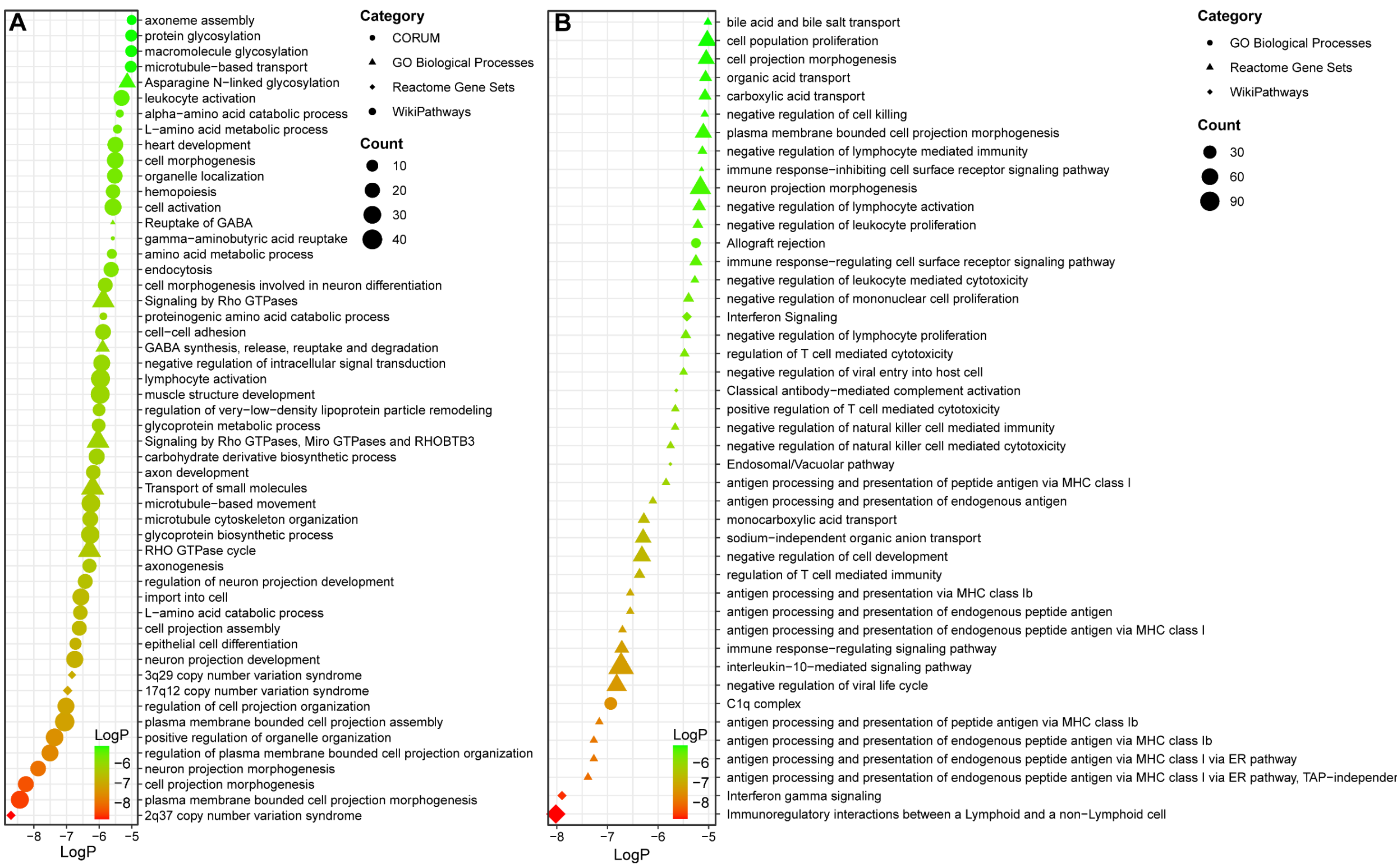

Figure S38. The bubble chart shows biological pathways that have been selected for analysis. The significant biological pathways obtained based on the Neanderthal (**A**) and Denisovan-derived sequences (**B**). Biological pathways with FDR < 0.05 and log10(p-value) < -5 were selected for bubble plot generation.

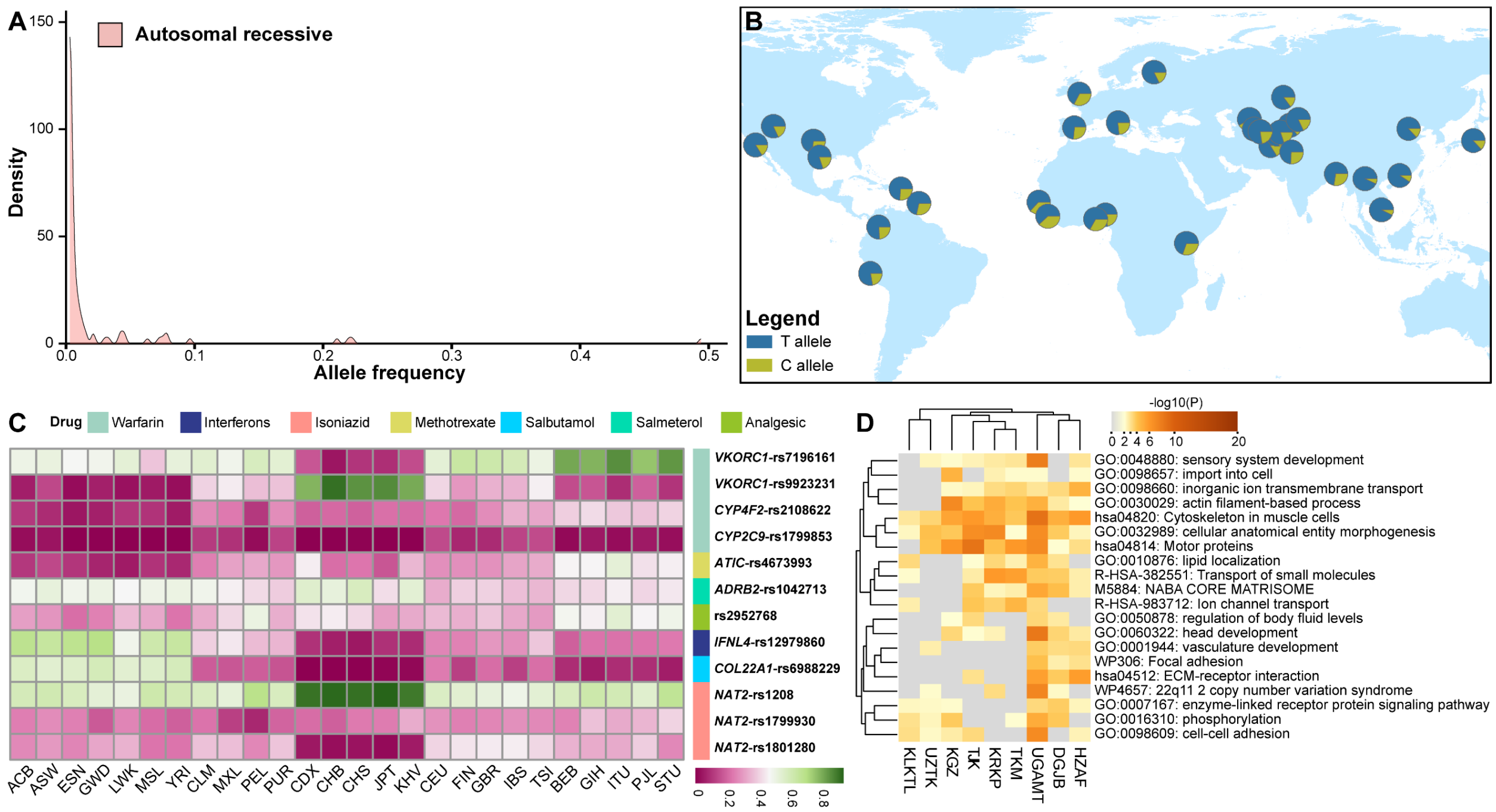

Figure S39. The distribution of medically relevant and pharmacogenomic variants. (**A**) Density plot of frequencies of pathogenic and likely pathogenic ClinVar variants, inherited in an autosomal recessive manner. (**B**) Allele frequency distribution of *SCN5A*-A1673G (rs1805124, H558R) variant. (**C**) Detailed allele frequencies of known pharmacogenomic variants identified in CAAH. ACB: African Caribbean in Barbados, ASW: African Ancestry in Southwest US, ESN: Esan in Nigeria, GWD: Gambian in Western Division-Mandinka, LWK: Luhya in Webuye, Kenya, MSL: Mende in Sierra Leone, YRI: Yoruba in Ibadan, Nigeria, CLM: Colombian in Medellin, Colombia, MXL: Mexican Ancestry in Los Angeles, California, PEL: Peruvian in Lima, Peru, PUR: Puerto Rican in Puerto Rico, CDX: Xishuangbanna Dai Chinese, CHB: Beijing Han Chinese, CHS: Southern Han Chinese, JPT: Tokyo Japanese, KHV: Ho Chi Minh Kinh, CEU: Utah residents with Northern and Western European ancestry, FIN: Finnish in Finland, GBR: British in England and Scotland, IBS: Iberian populations in Spain, TSI: Toscani in Italy, BEB: Bengali in Bangladesh, GIH: Gujarati Indian in Houston, Texas, ITU: Indian Telugu in the UK, PJL: Punjabi in Lahore, Pakistan, STU: Sri Lankan Tamil in the UK. (**D**) Enrichment results of heterozygous protein-truncating variants identified in CAAH.

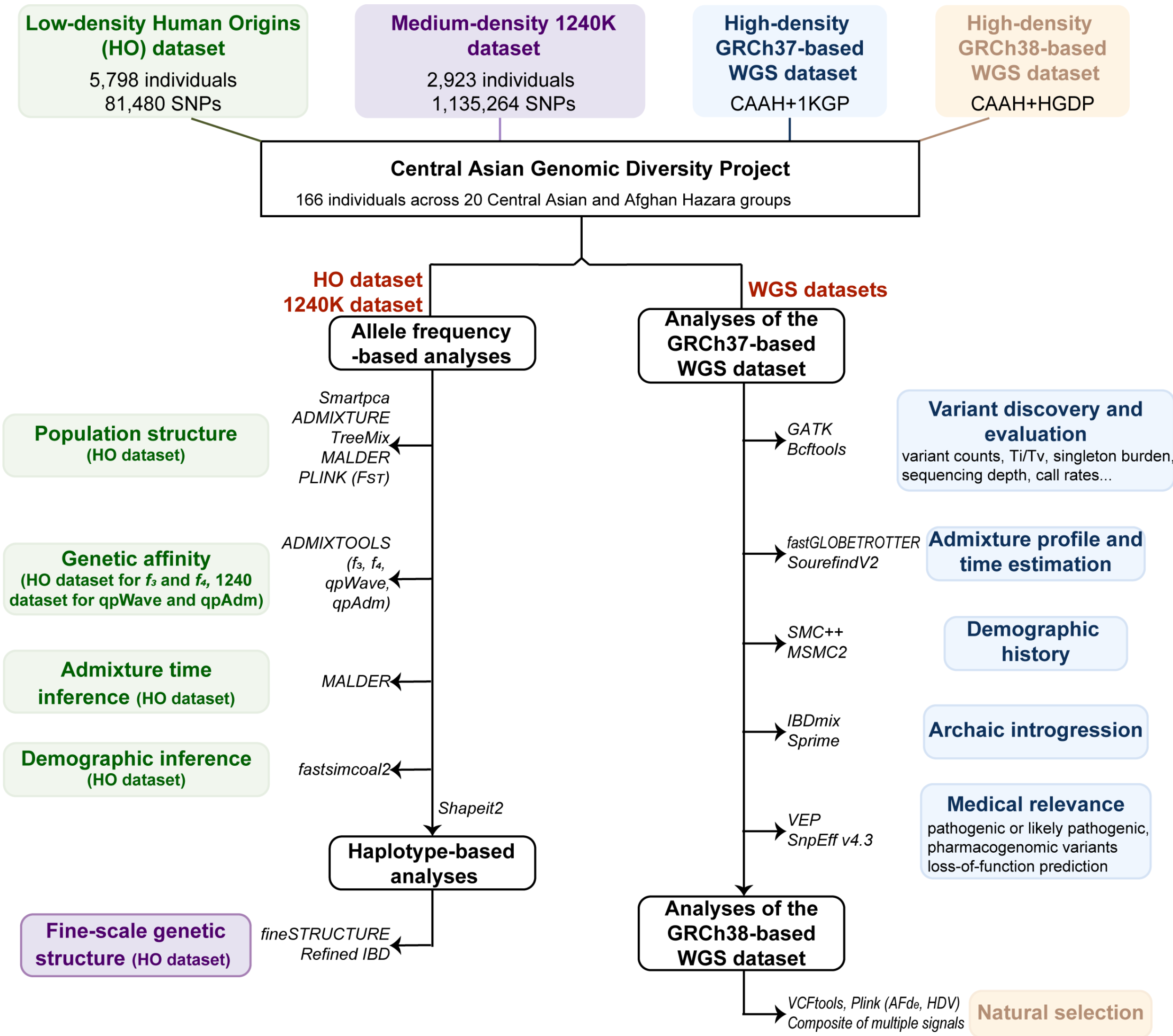

Figure S40. Schematic overview of the analytical framework used in this study. The schematic summarizes the datasets and tools applied throughout the analysis. WGS, whole-genome sequencing; GATK, Genome Analysis Toolkit; VEP, Variant Effect Predictor; HDV: highly differentiated variant.
